# An Investigation into Anion Sensing of the Molecular Aggregate of 4-(Pyrrol-1-yl)pyridine and Its Derivatives

**DOI:** 10.3390/molecules29235692

**Published:** 2024-12-02

**Authors:** Mallory E. Thomas, Lynn D. Schmitt, Alistair J. Lees

**Affiliations:** 1 Department of Chemistry, Binghamton University, Binghamton, NY 13902, USA; 2 Department of Chemistry, SUNY Cortland, Cortland, NY 13045, USA

**Keywords:** anions, chemodosimeter, chemosensor, molecular aggregate, nitrite, selectivity, sensitivity, 4-(pyrrol-1-yl)pyridine, 4-(2,5-dimethyl-pyrrol-1-yl)pyridine, 4-(2,4-dimethyl-pyrrol-1-yl)pyridine

## Abstract

Recently, 4-(pyrrol-1-yl)pyridine has been found to act as a supramolecular chemodosimeter, sensing nitrite ions in an aqueous solution with naked eye detection and a low limit of detection of 0.330 ppm. This work explores the anion-sensing properties of related derivatives, 4-(2,5-dimethyl-pyrrol-1-yl)pyridine and 4-(2,4-dimethyl-pyrrol-1-yl)pyridine, and provides a comparison with the parent compound. These molecules are determined to be effective sensors for nitrite ions with limits of detection of 1.06 ppm and 1.05 ppm, respectively. The high sensitivity and selectivity to nitrite remain even in the presence of competing anions such as SO_3_^2−^, NO_3_^2−^, PO_4_^3−^, SO_4_^2−^, Cl^−^, F^−^, I^−^, Br^−^, AcO^−^, and CN^−^. Analogous to the 4-(pyrrol-1-yl)pyridine system, the sensing mechanism appears to be the result of changes in the supramolecular aggregate system upon the interaction of an anion; this is further explored through dynamic light scattering, the Tyndall effect, and NMR spectroscopy. The two methylated derivative systems reported herein, 4-(2,5-dimethyl-pyrrol-1-yl)pyridine and 4-(2,4-dimethyl-pyrrol-1-yl)pyridine, are shown to affect the size of the supramolecular system and provide further insight into the unique mechanism of action.

## 1. Introduction

The detection of anions is of particular importance due to their ubiquitous nature, various sizes, charges, and overall diverse physiochemical traits. Nitrite, in particular, is commonly used as a preservative in consumer goods, and has its primary use in fertilizers [1,2,3,4,5]. Due to this, the U.S. Environmental Protection Agency (EPA) has set a maximum contaminate level (MCL) of nitrite at 1 ppm [1]. An increased intake of nitrite has been discovered to have possible connections to both colorectal and stomach cancers, and has additional negative health effects such as shortness of breath, blue baby syndrome, and hypoxia [3,6,7,8,9]. This has also caused a set limit of 3 ppm of nitrite in drinking water from the World Health Organization (WHO) [5]. Additionally, from excessive use of fertilizer and its exceedingly important role in the nitrogen cycle, increased concentrations of nitrite in water systems can lead to the eutrophication of waterways that affects both aquatic life and overall water quality [10,11,12,13]. General methods for the detection of nitrite include electrochemical methods [14,15], fluorescence spectroscopy [16,17,18,19], and UV–visible spectroscopy [18], among others. However, many of these methods come with challenges such as a high cost and intricate experimental steps, and are susceptible to interference [20,21,22,23].

The presence of anions in food, agriculture, and environmental media has highlighted the need for quick, low-cost, and highly sensitive means for a qualitative analysis. Hence, there is the need for chemosensors and chemodosimeters. Chemosensors are substances that exhibit a change in their spectroscopic properties when a target analyte is present. This process is described by an intricate balance in interaction between the receptor (chemosensor) and the analyte (anion) where the recognition of the analyte is illustrated by a photophysical, electrochemical, or colorimetric change in the receptor; this interaction is ultimately reversible in nature. On the other hand, if the reaction taking place is irreversible, the system is described as a chemodosimeter [24,25,26].

Recently gaining popularity as chemosensors and chemodosimeters are supramolecular (i.e., aggregate) species. The ability to tune their spectroscopic properties makes them ideal candidates for anion sensing due to the capacity to tailor their function [27,28]. Many supramolecular systems are found in nature such as coenzyme B_12_, chlorophyll, and cytochromes [29]. Here, their presence has been prevalent in cation sensing, commonly detecting cations such as Cr^2+^, Cu^2+^, and Zn^2+^ [30,31,32]. However, large conjugated systems that exhibit π-stacking, such as porphyrins, have previously been employed for their use in anion sensing [33]. Here, manipulations of N-H bonds are commonly exploited to form intermolecular interactions with target anions of interest [25,34,35]. However, other methods of detection through usage of various intermolecular interactions such as π-stacking, H-bonding, and hydrophobic forces can also be utilized for anion-sensing purposes [24,25]. Most notably, aggregate supramolecular systems have been discovered to be an asset to the mechanics of anion sensing.

Aggregates commonly exist in two forms, J-aggregates and H-aggregates [36,37,38,39,40,41]. Within these arrangements, two-dimensional structures are formed. Some of these formations include brickwork, staircase, and ladder [42,43,44]. The intermolecular interaction holding each monomer together is determined by the structure of the compound. Typically, planar, conjugated systems, such as porphyrins or heterocycles, exhibit π-stacking interactions with neighbouring molecules. Previous work published by this group depicts the aggregation ability of 4-(pyrrol-1-yl)pyridine to form π-stacked aggregates in an aqueous solution due to the planar conjugated electron system [45]. The intricate characteristics of aggregated systems can cause enhanced effects in their ability to detect anions of interest.

Here, we describe the properties of a new sensor class based on structural modifications of the previously reported 4-(pyrrol-1-yl)pyridine (PP) molecule [45]. Modifications by the addition of methyl substituents to the pyrrole moiety generate the new derivatives 4-(2,5-dimethyl-pyrrol-1-yl)pyridine (2,5-PP) and 4-(2,4-dimethyl-pyrrol-1-yl)pyridine (2,4-PP) as possible sensors. Previously, NMR spectra illustrated that the preferred site of electrostatic interaction with a nitrite ion was on the alpha position of the pyrrole moiety of PP. An introduction of methyl groups to these positions may enhance or impair the sensitivity or selectivity of this system to nitrite ions. These newly synthesized molecules reveal key insights into the delicate and unique mechanism of action on a supramolecular scale. Through these variations, we depict how sensitivity and selectivity changes to target analytes can be modified, and how supramolecular systems can be altered to provide new possibilities in anion sensing.

## 2. Results and Discussion

### 2.1. Characterization of PP

A full characterization of PP was previously reported by this group [45]. This includes ^1^H, ^13^C, COSY, and HSQC NMR; it also includes a DLS analysis and reports results of the Tyndall effect. These results are referenced throughout this article.

#### 2.1.1. NMR Characterization of 2,5-PP

**Figure 1:**
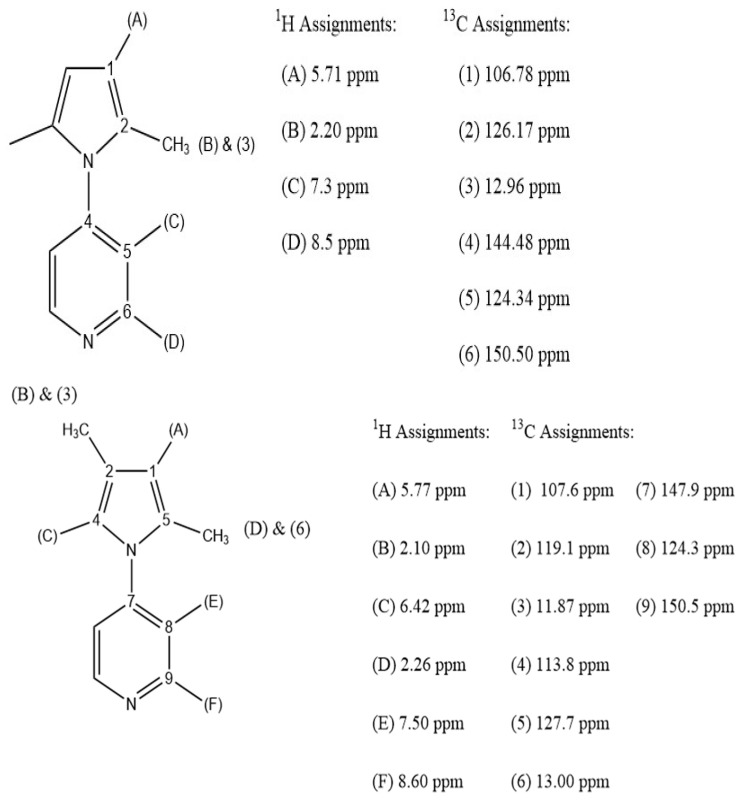
^1^H and ^13^C NMR assignments of 4-(2,5-dimethyl-pyrrol-1-yl)pyridine (**top**) and 4-(2,4-dimethyl-pyrrol-1-yl)pyridine (**bottom**) in CDCl_3_.

The confirmation of the product 2,5-PP (30 mM) via ^1^H NMR in CDCl_3_ yields δ8.50 ppm (dd, *J* = 4.8 Hz, 2H), δ7.30 ppm (dd, *J* = 4.8 Hz, 2H), δ5.71 ppm (d, *J* = 4.0 Hz, 2H), and δ2.20 ppm (s, 6H). Additionally, ^13^C NMR assignments further confirm the creation of 2,5-PP with the following chemical shifts: δ106.78, δ126.17, δ12.96, δ144.48, δ124.34, and δ150.50 ppm. This was found to be similar with previously determined NMR positions accounting for the CDCl_3_ solvent and matched the splitting pattern expected from the starting reagents with pyrrole peaks shifted up-field by approximately 0.2 ppm. ^1^H and ^13^C NMR assignments are depicted on the structure of 2,5-PP and 2,4-PP shown in Figure 1. These spectra and two-dimensional ^1^H NMR (COSY, HSQC) further confirm the synthesis by depicting H-bond interactions between the pyridine hydrogens on the molecule; see Supporting Information.

**Figure 2:**
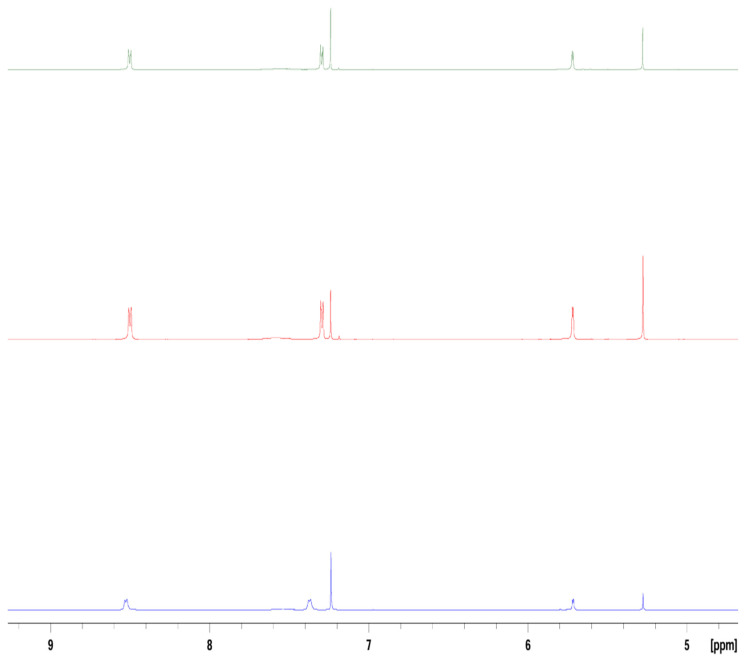
^1^H NMR in the aromatic region of 4-(2,5-dimethy-pyrrol-1-yl)pyridine from top to bottom: 120 mM, 90 mM, and 30 mM in CDCl_3_.

Concentration-dependent NMRs were performed to determine the extent of intermolecular interactions that can contribute to peak shifting and broadening. A 30 mM sample of 2,5-PP yields sharp peaks with clear splitting, and is consistent with the identical concentration in d-DMSO. However, more concentrated samples of 2,5-PP of 90 mM and 120 mM in CDCl_3_ depict slight shifting of pyridine protons up-field; see Figure 2. This up-field shift is consistent with increased electron density coming from the methyl groups on the system. Lower-concentration NMR spectra also exhibit slight broadening in the peaks. Increasing the number of scans can help to elucidate the true peak splitting pattern, and also serves to justify slight differences in splitting. This was consistent with the phenomenon seen with the 4PP system where an increased concentration caused peak broadening and shifting from 30 mM to 120 mM [45].

Solvents such as d-DMSO and D_2_O are known to interrupt intermolecular interactions as opposed to the use of CDCl_3_. Upon the NMR analysis of 30 mM, 90 mM, and 120 mM of 2,5-PP in d-DMSO, all spectrum peaks exhibited clear splitting regardless of the concentration of the sample. Thus, it is inferred that the 2,5-PP system is also an aggregate similar to that of its analogue, PP. The extent of aggregation will be discussed in further sections.

#### 2.1.2. NMR Characterization of 2,4-PP

The confirmation of the product 2,4-PP (30 mM) via ^1^H NMR in CDCl_3_ yields δ8.60 ppm (dd, *J* = 2.94 Hz, 2H), δ7.50 ppm (dd, *J* = 2.98 Hz, 2H), δ5.77 ppm (s, 1H), δ6.42 ppm (s, 1H), and δ2.10 ppm (s, 6H). Additionally, ^13^C NMR assignments further confirm the creation of 2,4-PP with the following chemical shifts: δ107.6, δ119.1, δ13.0, δ147.9, δ124.3, δ127.7, δ113.8, δ11.87, and δ150.5 ppm. ^1^H and ^13^C NMR assignments are assigned on the structure of 2,4-PP in Figure 1. Two-dimensional ^1^H NMR (COSY, HSQC) additionally confirms the synthesis by illustrating H-bond interactions between the pyridine hydrogens on the molecule; see Supporting Information. This is in agreement with that seen in the 2,5-PP system, as it contrasts with the PP system.

**Figure 3:**
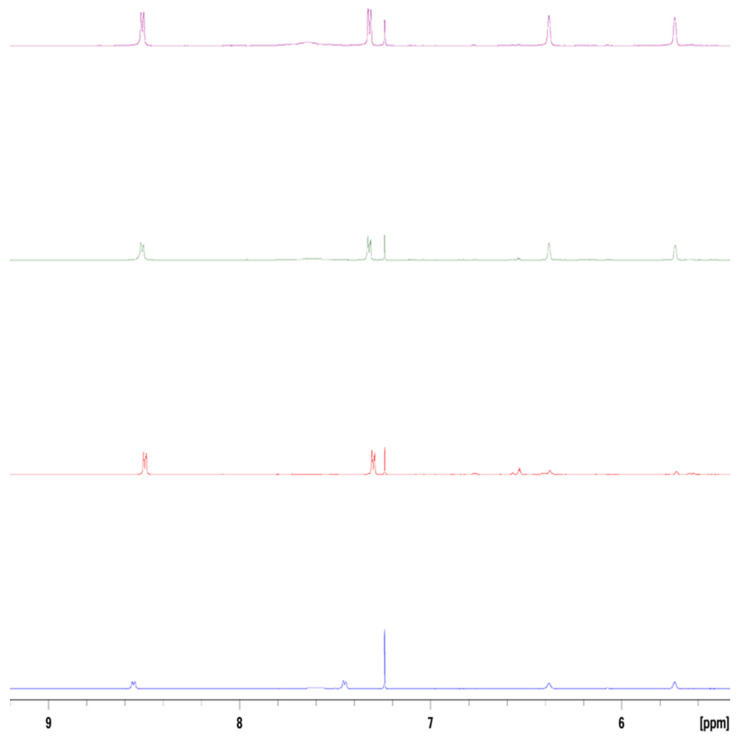
^1^H NMR of 4-(2,4-dimethyl-pyrrol-1-yl)pyridine from top to bottom: 120 mM, 90 mM, 60 mM, and 30 mM in CDCl_3_.

Consistent with both that of the 2,5-PP and PP systems, concentration-dependent NMRs were performed to determine the extent of intermolecular interactions that can contribute to peak shifting and broadening. After increasing the concentration of 2,4-PP from 30 mM to both 90 mM and 120 mM in CDCl_3_, the spectra reveal slight up-field shifts in all protons; see Figure 3. This was previously observed for the 2,5-PP system from the contribution of the methyl substituents in the sensor system, and the broadening was consistent with both 2,5-PP and PP systems.

Upon the NMR analysis of 30 mM, 90 mM, and 120 mM of 2,4-PP in d-DMSO, all NMR spectra showed clear splitting at all concentrations; see Supporting Information. As stated previously, since d-DMSO and D_2_O are solvents known to disrupt intermolecular interactions, it can be concluded that the 2,4-PP system is an aggregate like that of the other two structures, 2,5-PP and PP. The NMR analysis in D_2_O provides a higher baseline in the spectra, resulting from an increased necessity to suppress the water hydrogen peaks. More on the extent of aggregation is covered in future sections.

### 2.2. UV–Visible Spectroscopy of 2,5-PP and 2,4-PP, with Comparison to PP

**Figure 4:**
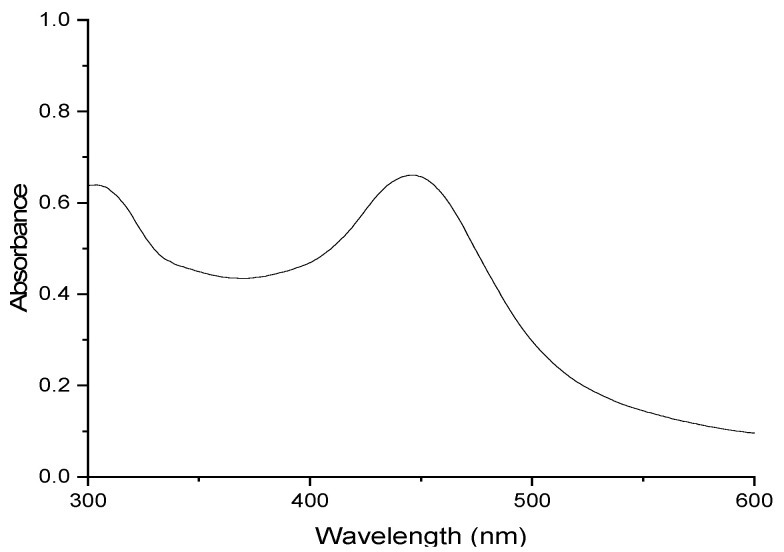
Absorbance spectrum of 2.20 × 10^−3^ M 4-(2,5-dimethyl-pyrrol-1-yl)pyridine (2,5-PP) in pure water.

Aqueous solutions of 2,5-PP from the residual oil appear yellow-orange in colour and have a broad band maximum at 446 nm. This varies from that of the PP system by 17 nm with the latter having an absorbance maximum at 463 nm. The absorbance spectrum in pure water is illustrated in Figure 4, and was corrected for the solvent. No fluorescence was exhibited in the solution.

Dilutions of aqueous 2,5-PP depict a molar absorptivity of 317 M^−^^1^cm^−^^1^. Subsequently, solvent dependence was carried out using diethyl ether, chloroform, acetone, methanol, dimethyl sulfoxide, and water. Here, in all solvents except water, the absorbance was much lower and broader in comparison. Consistent with that of the PP system having a molar absorptivity of 110 M^−^^1^cm^−^^1^, the low molar absorptivity is unusual for the average monomer electronic transition; this again illustrates a highly unusual electronic transition, alluding to potential aggregation.

**Figure 5:**
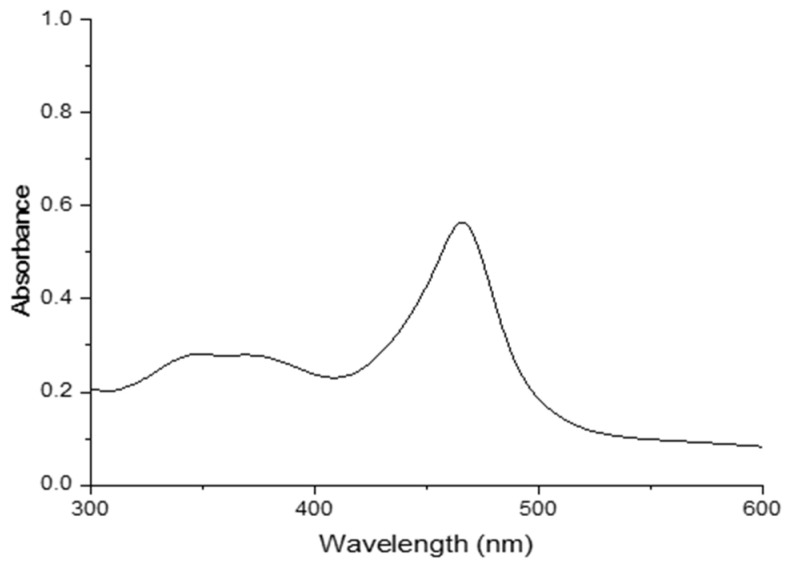
Absorbance spectrum of 3.60 × 10^−3^ M aqueous 4-(2,4-dimethyl-pyrrol-1-yl)pyridine (2,4-PP) in pure water.

On the other hand, the 2,4-PP oil, once dissolved in water, appears yellow in colour and depicts a visible band absorbance maximum at 466 nm. This varies from the band maxima previously determined for 2,5-PP by 20 nm and that of PP by only 3 nm. Here, all three systems show similar absorbance spectra and colour in aqueous solutions. The absorbance spectrum of aqueous 2,4-PP is depicted in Figure 5. The solution also did not exhibit fluorescence.

Dilutions of aqueous 2,4-PP illustrate a molar absorptivity of 167 M^−^^1^cm^−^^1^. This is consistent with the magnitude seen for molar absorptivity of 2,5-PP and PP. Solvent dependence was carried out, with the identical solvents to that of both 2,5-PP and PP, and revealed a bathochromic shift with increasing solvent polarity. Similarly, across all three systems, the UV–visible spectra exhibit an absorbance tail at long wavelengths: this is indicative of molecular aggregation and light scattering. It was also observed that the absorbance maxima at 446 nm (2,5-PP) and 466 nm (2,4-PP) were unshifted at concentrations as low as 1 × 10^−^^5^ M as observed using a 10 cm pathlength cell.

### 2.3. Nitrite-Sensing Capabilities of 2,5-PP and 2,4-PP Systems

#### 2.3.1. 2,5-PP Solutions

**Figure 6:**
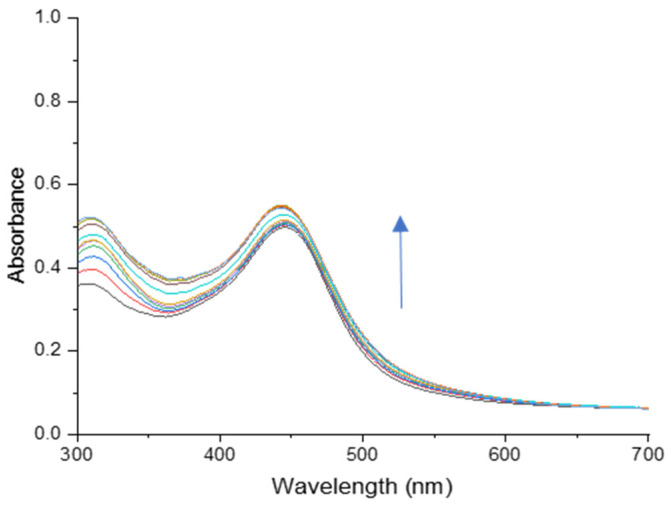
Absorbance spectra of a titration between 1.56 × 10^−3^ M 4-(2,5-dimethyl-pyrrol-1-yl)pyridine (2,5-PP) (aq) and 10.00 µL additions of 0.01 M sodium nitrite (aq) in pure water. Absorbance increase upon successive additions is indicated by the arrow.

The anion-sensing capabilities of 2,5-PP were explored analogous to those of the PP system. Upon the addition of aqueous sodium nitrite, the system had a lack of colorimetric change, and an increase in absorbance in the ultraviolet region. This was unusual as the PP system had shown a distinct colorimetric change from yellow to pink upon the addition of nitrite. In the case of 2,5-PP, the absorbance at 309 nm continued to increase with each addition of nitrite. Figure 6 illustrates a titration monitored by UV–visible spectroscopy between aqueous 2,5-PP and NaNO_2_ (aq.).

The yellow-orange sensor absorbance band at 446 nm depicts a slight increase in absorbance, but it does not result in a naked eye colour change. More importantly, the increase in 309 nm with each addition of nitrite is more prominent and is employed for LOD calculations to provide a more accurate result. The lack of the bathochromic shift seen with the PP system to nitrite alludes to a different mechanism of interaction between 2,5-PP and nitrite anions.

**Figure 7:**
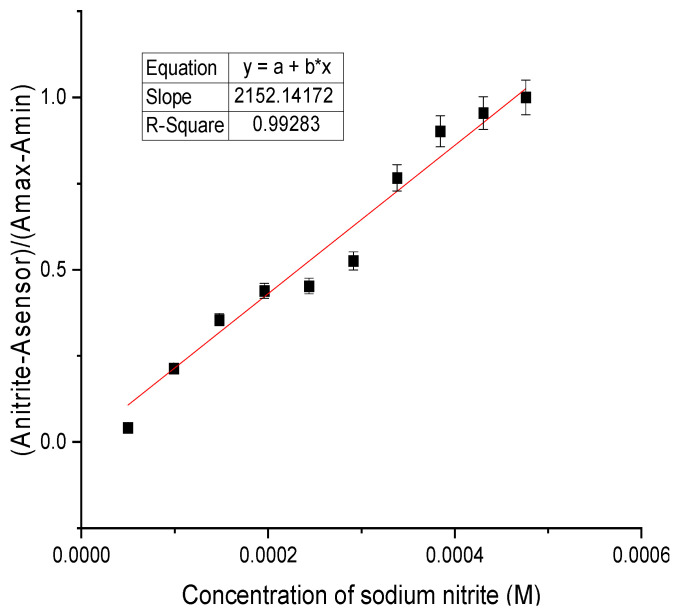
Plot for limit of detection of 1.56 × 10^−3^ M aqueous 4-(2,5-dimethyl-pyrrol-1-yl)pyridine (2,5-PP) with 10.00 µL additions of 0.01 M aqueous sodium nitrite.

Each nitrite addition was found to be irreversible over time, and confirmed that the 2,5-PP system is acting as a chemodosimeter. From these results, an LOD was determined for this system via a plot of the absorbance changes at 309 nm versus added sodium nitrite concentration; see Figure 7. The LOD was calculated using Equation (1), as indicated below. Here, S is the slope of the line generated, and σ is the standard deviation of the blank [46].

LOD = (3 σ/S)(1)

A value of 1.06 ppm (1.54 × 10^−^^5^ M) was subsequently obtained for the change in 2,5-PP upon the addition of aqueous sodium nitrite. LOD measurements for all trials were typically reproducible with error within 4%. Revealing a high sensitivity of the sensor to nitrite ions, this was found to be consistent and unaffected by the initial sensor concentration. These results display that by blocking both the 2 and 5 alpha positions on the pyrrole moiety of the sensor, the sensitivity decreases significantly and the mechanism through which it functions is altered. The sensitivity of 2,5-PP to nitrite was determined to be three times less sensitive than that of PP (0.330 ppm) [45].

#### 2.3.2. 2,4-PP Solutions

**Figure 8:**
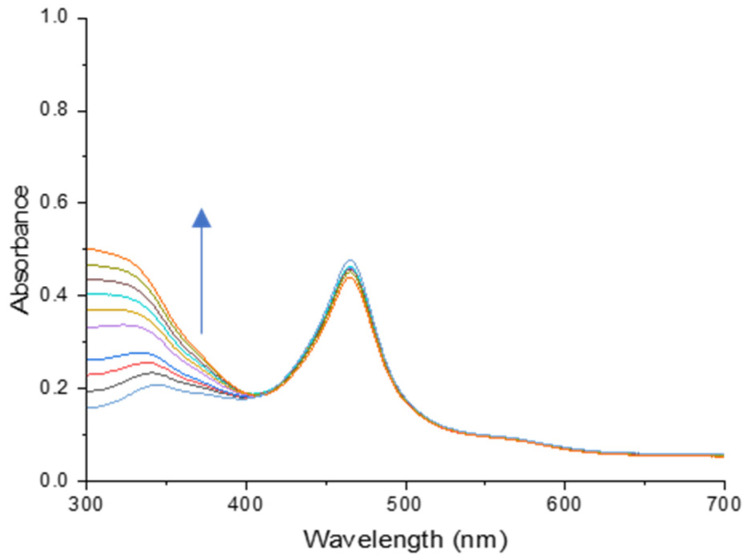
Absorbance spectra of a titration between 1.46 × 10^−3^ M aqueous 4-(2,4-dimethyl-pyrrol-1-yl)pyridine (2,4-PP) and 10.00 μL additions of 0.01 M aqueous sodium nitrite. Absorbance increase is indicated by the arrow upon successive additions of nitrite.

Anion testing utilizing 2,4-PP was performed in a similar fashion to the previous 2,5-PP and PP systems. After the addition of aqueous sodium nitrite (0.01 M), 2,4-PP did not exhibit a colorimetric change, but the absorbance at 341 nm increased with each successive addition. This was similar to that observed from 2,5-PP, illustrating an increase in absorbance in the ultraviolet region, and is shown in Figure 8.

**Figure 9:**
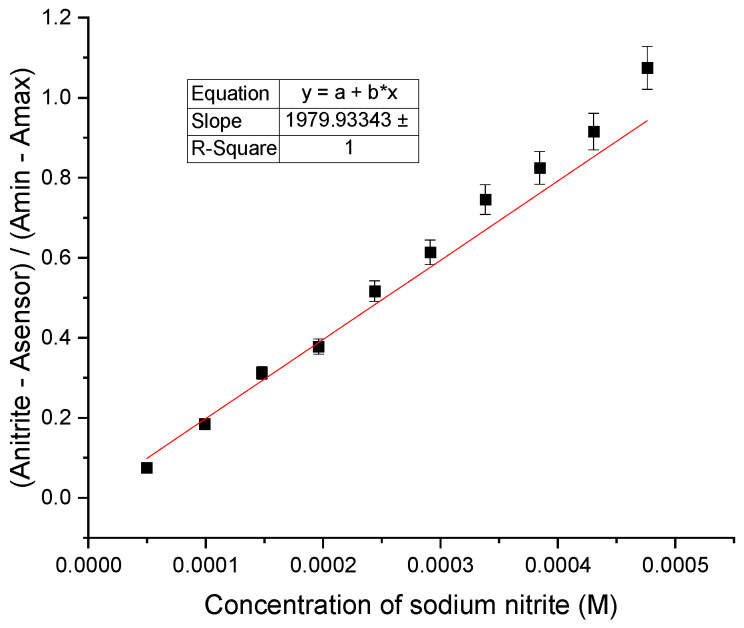
Limit of detection determination of 1.46 × 10^−3^ M 4-(2,4-dimethyl-pyrrol-1-yl)pyridine (2,4-PP) from plot of absorbance changes at 341 nm upon successive addition of aqueous sodium nitrite in pure water.

Here, the addition of nitrite is again shown to be irreversible over time, consistent with that of both the PP system and the 2,5-PP system. This indicated that all three systems maintain their ability to act as a chemodosimeter towards nitrite regardless of the structural modifications of the methyl substituents. From there, an LOD was determined for the 2,4-PP system using a plot of absorbance changes at 341 nm and sodium nitrite concentration; see Figure 9. The LOD was calculated using Equation (1), as previously stated.

A value of 1.05 ppm (1.52 × 10^−^^5^ M) was obtained for the change in 2,4-PP with the addition of aqueous sodium nitrite (0.01 M). This was consistent with errors typically around 5%. This result is almost identical to that of the 2,5-PP system, falling within the range of error obtained for 2,5-PP to nitrite, and was still three times less sensitive than the PP system. Here, the sensitivity proves to be unaffected by the location of the two methyl substituents where either both preferential alpha locations of interaction are sterically hindered, or where only one location is affected. These results were shown to be unaffected by initial starting sensor concentration. While the sensitivity to nitrite ions in the 2,4-PP system did not change significantly from that of the 2,5-PP system, the mechanism through which it functions may be different due to the increase at varying absorbance maxima for the two systems.

### 2.4. Competitive Anion Studies of 2,5-PP and 2,4-PP

#### 2.4.1. 2,5-PP

The results obtained for 2,5-PP were determined to be highly reproducible within a working pH range of 1–7. A phosphate buffer was employed to maintain the pH at both 5.0 and 6.7, and this did not result in any changes in the sensor’s ability to detect nitrite ions. Carbonate anions were not employed in the competitive anion study due to their ability to neutralize the pH of the working matrix. At basic pH (>7), the sensor did not show any interaction with nitrite ions, and shows the spectroscopic degradation of the system. Aqueous solutions of 2,5-PP were also shown to be stable at room temperature for 24 h. With these reaction parameters, the sensor was reproducible with LOD values falling in the range as stated previously for all acidic (pH < 7) conditions. Furthermore, the oil samples of 2,5-PP from the extraction process were stable upwards of 3 months upon storage at 277 K. All of these characteristics were concordant with that exhibited by the PP system.

**Figure 10:**
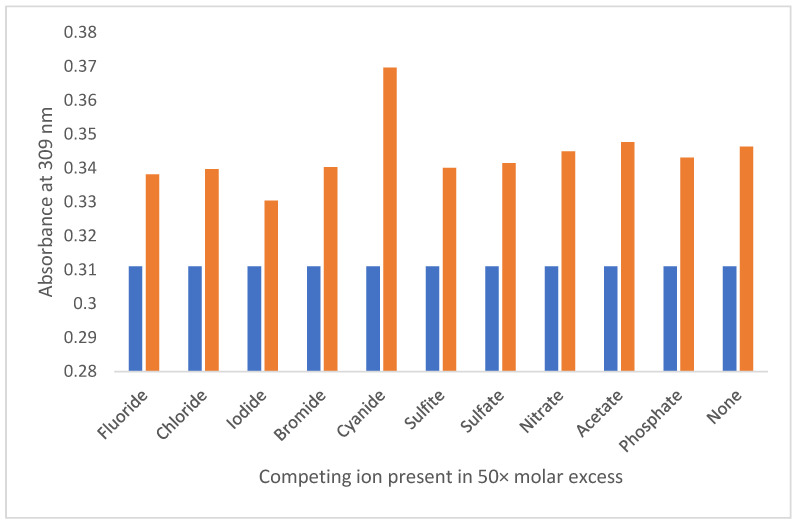
Absorbance at 309 nm of 9.81 × 10^-4^ M 4-(2,5-dimethyl-pyrrol-1-yl)pyridine (2,5-PP) sensor solution (aq) (R, blue) and sensor solution with addition of 10.00 µL of sodium nitrite (aq) (0.01 M) and competing anion (0.50 M) (L, orange) in pure water.

The selectivity of this system was investigated with a competitive anion study, akin to the method used for the PP system after the analysis upon each anion individually. Other anions apart from nitrite showed no spectroscopic changes when studied individually. Competing anions of sulfite, nitrate, phosphate, sulphate, chloride, fluoride, iodide, bromide, acetate, and cyanide were used in fifty molar equivalents to nitrite. After an addition of 10.00 µL of each 1:50 aqueous solution, none displayed any affinity or competitiveness towards 2,5-PP. Additionally, in all cases, an increase in absorbance at 309 nm upon the addition of nitrite was unaffected by the competing anion present; see Figure 10.

Therefore, the 2,5-PP system was found to be both sensitive and selective to nitrite ions in the aqueous solution, identical to that of the original PP system [45]. The nitrite concentration in the solution (4.98 × 10^−^^5^ M) is much lower than that of the sensor (1.90 × 10^−^^2^ M). This indicates that the interaction here is indicative of deviations from a normal 1:1 or 1:2 stoichiometry. With the lack of the formation of a new absorbance peak, seen in the 4PP system with a colour change to pink upon nitrite addition, and lower extent of scattering at long wavelengths, the aggregate size and mechanism of function for 2,5-PP were studied. These results are discussed further, vide infra.

#### 2.4.2. 2,4-PP

In agreement with both the PP system and the 2,5-PP system, pH stability of the 2,4-PP system was from 1 to 7, with basic pH conditions unable to interact with nitrite anions, and also depicting structural degradation. A phosphate buffer was employed to maintain the pH at both 5.0 and 6.7, and this did not result in any changes in the sensor’s ability to detect nitrite ions. In addition, the 2,4-PP system was stable at room temperature for 24 h. Lastly, similar with the previous results, the extracted oil of 2,4-PP was stable for upwards of 3 months when stored at 277 K.

**Figure 11:**
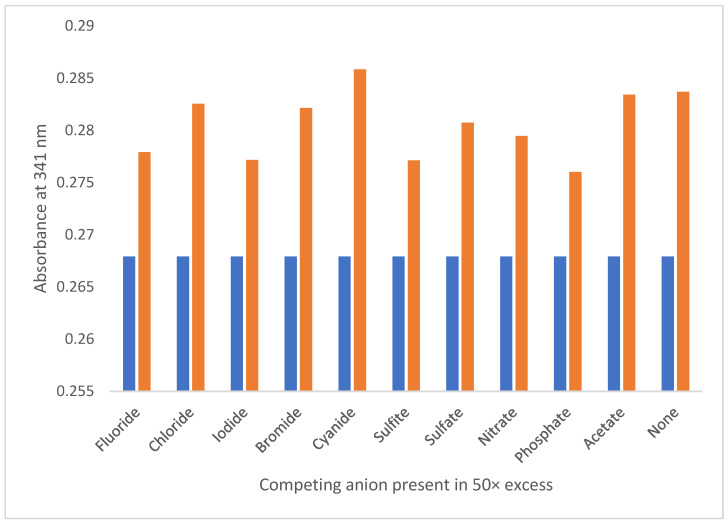
Absorbance at 341 nm of 1.59 × 10^−3^ M 4-(2,4-dimethyl-pyrrol-1-yl)pyridine (2,4-PP) sensor solution (aq) (R, blue) and sensor solution with addition of 10.00 µL of aqueous sodium nitrite (0.01 M) and competing anion (0.50 M) (L, orange) in pure water.

The selectivity of the 2,4-PP sensor was also investigated with a competitive anion study, as mentioned for the 2,5-PP system. Competing anions were again employed in 1:50 molar equivalents to nitrite. After the addition of each 1:50 solution (10.00 µL), none of the competing anions exhibited any effect on 2,4-PP. In addition, an increase in absorbance at 341 nm upon the addition of nitrite was shown in all cases for solutions in acidic conditions (pH < 7). This is shown in Figure 11.

Overall, the 2,4-PP system was found to be highly sensitive and selective to nitrite ions; this is concordant with both the 2,5-PP system and the original PP system. The significant spectroscopic change upon a singular addition of nitrite (5.00 × 10^−^^5^ M) to the starting 2,4-PP sensor (1.46 × 10^−^^3^ M) is again inferring the presence of a supramolecular system that does not follow a 1:1 or 1:2 stoichiometry. Based off the analysis of the 2,5-PP system, the mechanism of sensing was studied for 2,4-PP, vide infra.

### 2.5. Investigation into the Supramolecular Method of Sensing

#### The Tyndall Effect of 2,5-PP, 2,4-PP, and PP Solutions

**Figure 12:**
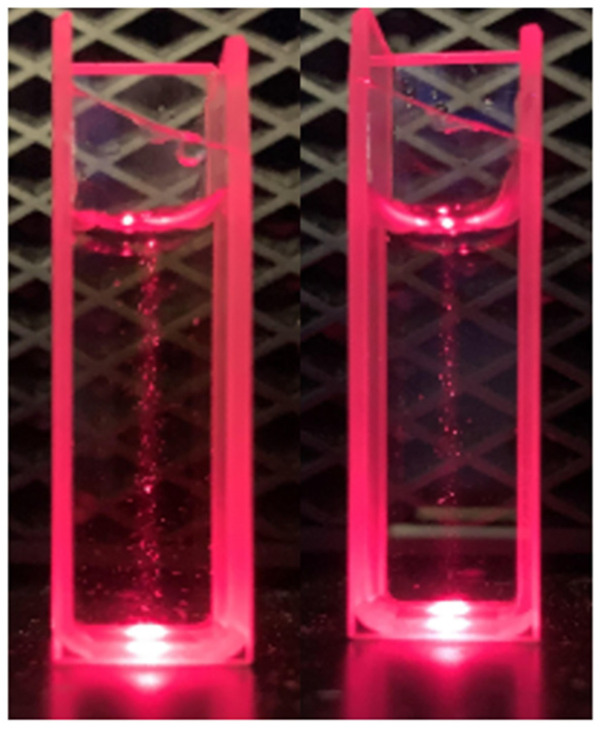
Tyndall effect of solutions of aqueous 4-(2,5-dimethyl-pyrrol-1-yl)pyridine (2,5-PP) at (**right**) 2.81 × 10^−3^ M and at (**left**) 7.29 × 10^−3^ M.

The Tyndall effect was employed for aqueous solutions of 2,5-PP; see Figure 12. Scattering was observed with solutions as dilute as 1.25 × 10^−^^5^ M.

In addition, DLS studies at a range of concentrations of 2,5-PP reveal an overall smaller aggregate, with a smaller range of aggregate size compared to the PP system [45]. The PP system revealed a range of aggregate size from 142 to 363 nm [45]. A monomer of 2,5-PP is roughly 1.08 nm in length, utilizing average bond distances. Using the average distance of π-stacking between aggregates (3.55 Å), a solution of 2,5-PP contains approximately 738–831 monomers; the PP system had a range of 400–1200 monomers [45].

Due to the presence of an aggregate in the solution, the NMR characterization of the final product was problematic. However, the comparison of the ^1^H NMR of a solution of 2,5-PP with and without the addition of nitrite in d-DMSO revealed no structural changes in the molecule. The ^1^H NMR spectra of 2,5-PP with and without the addition of nitrite can be seen in the Supporting Information. In addition, the COSY NMR spectrum revealed no changes in the coupling of protons in the molecule; see Supporting Information. On the other hand, the COSY spectrum from PP revealed a loss in coupling of the pyrrole protons, indicating that the nitrite ion is interacting at the pyrrole moiety [45]. It is again hypothesized that the nitrite is not covalently binding to the pyrrole moiety, but is exhibiting an electrostatic interaction with the pyrrole ring of the 2,5-PP system. Here, in comparison to that of the PP system, the LOD was expected to decrease due to the structural changes at the alpha positions of the pyrrole moiety. However, the beta positions on the pyrrole ring would be the only available location for the nitrite to interact on the pyrrole moiety; this is also affected by steric hinderance of the nearby methyl groups. This further supports the lack of structural changes in both the ^1^H and COSY NMR spectra, and is in accordance with the PP system.

**Figure 13:**
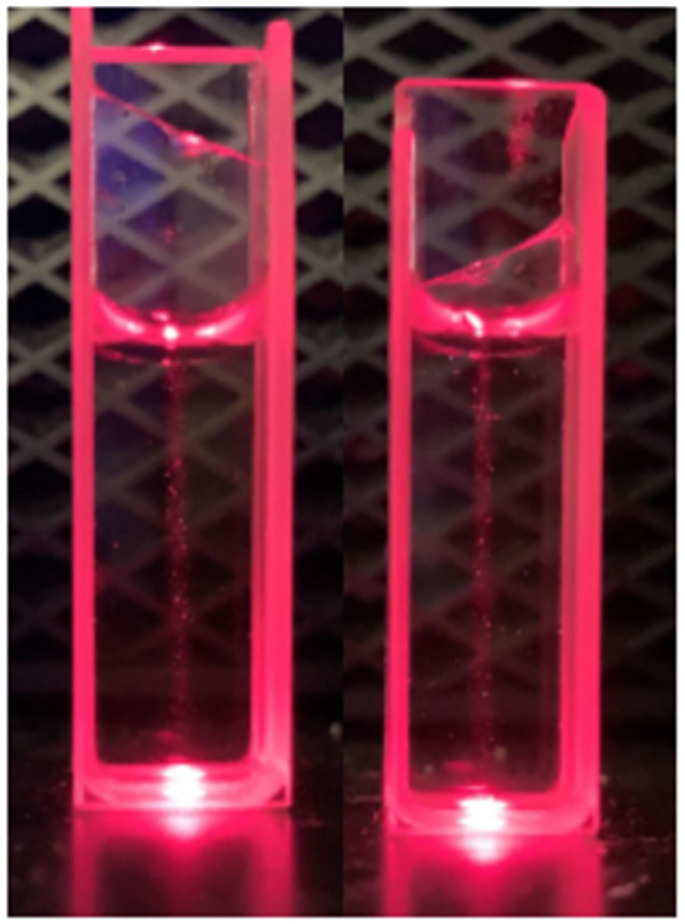
Tyndall effect of 2.13 × 10^−3^ M solution of aqueous 4-(2,4-dimethyl-pyrrol-1-yl)pyridine (2,4-PP) (**left**) and 5.62 × 10^−4^ M aqueous 4-(2,4-dimethyl-pyrrol-1-yl)pyridine (**right**) both with light scattering present.

Comparable with the above systems, a similar Tyndall effect for 2,4-PP was observed, again indicating the molecular aggregation of the system; see Figure 13. Solutions as dilute as 1.25 × 10^−^^5^ M were shown to exhibit an extent of scattering.

### 2.6. DLS Analysis of 2,5-PP, 2,4-PP, and PP Systems

Akin to that of 2,5-PP and PP, DLS studies were also performed for 2,4-PP over various concentrations to determine aggregate size. More similar to the PP system, 2,4-PP showed increasing aggregate size with increasing concentration. Here, a similar range to the PP system was observed compared to 2,5-PP. Table 1 illustrates a comparison in the DLS data of all three samples. Using the same mathematical assumptions as the 2,5-PP system, a solution of 2,4-PP consists of approximately 538–989 monomers. This varies from the PP system with a range of 400–1200 monomers [45].

As previously noted, obtaining an NMR analysis on large supramolecular systems is challenging. However, comparing ^1^H NMR with and without nitrite in d-DMSO, no changes in the structure of the sensor were observed. In addition, the two-dimensional COSY NMR spectrum illustrated no deviations in proton coupling throughout the molecule. These spectra are depicted in the Supporting Information. In contrast, the COSY spectrum from PP revealed a loss in coupling of the pyrrole protons, indicating that the nitrite ion is interacting at the pyrrole moiety, as previously mentioned [45]. These results are consistent with that of the 2,5-PP system towards nitrite, and are again indicative of the strong electrostatic interaction of nitrite ions with these small-molecule-aggregate-based systems.

While the LOD for the 2,4-PP system was hypothesized to be both higher than the 2,5-PP system and lower than that of PP, the LOD was almost identical to the 2,5-PP sensor. Here, as one preferential alpha position is still open to nitrite, and the fact that the 2,5-PP system illustrates how the beta position can also be used in sensing, this is in line with the lack of structural changes in the 2,4-PP NMR spectra of the sensor–nitrite product. Table 2 summarizes the LODs obtained for all three systems.

### 2.7. The Influence of the Methyl Substituents on the Mechanism of Sensing

The presence of the moderately electron-donating methyl substituents to various positions on the pyrrole ring of the original PP system has proven to affect the sensitivity of the sensor to its analyte of interest. This manipulation on the pyrrole moiety causes an increase in electron density in the aromatic ring, and thus raises the highest occupied molecular orbital (HOMO) energy level. This effect then causes an overall increase in the energy gap between the HOMO and the lowest unoccupied molecular orbital (LUMO). Both computational and experimental studies note that the positive electrostatic potential on the π conjugated system is depleted by resonance in anion sensors that contain π electron-donating substituents; this is then conversely strengthened with π electron-withdrawing groups [47]. Consequently, better anion sensors can be created with reducing the π electron density entering the conjugated aromatic rings.

The increased electron density entering the ring raises the nucleophilic character of the pyrrole ring, and thus increased repulsion relative to that of the negatively charged ion. In addition to increased electron density, the presence of methyl substituents at both alpha positions, as seen in 2,5-PP, block the preferred “active site” for nitrite to interact with the pyrrole moiety of the sensor. It is naturally suggested that by blocking one of the preferential positions for interaction, the limit of detection would decrease from that of PP. This was indeed observed in the LOD value, decreasing by a factor of 3 from the unsubstituted system (0.330 ppm) to the 2,5-PP system (1.06 ppm).

On the other hand, in the 2,4-PP system, one of the preferential sites of interaction is blocked by a methyl group, leaving the 5-position open for electrostatic interaction with nitrite. However, the previous 2,5-PP system illustrated that both the beta positions (3 and 4) can interact with nitrite ions when the alpha positions are blocked. One could hypothesize that the LOD should be between that of PP and 2,5-PP. However, this was not the case with the experimental data, revealing an LOD almost identical to that of the 2,5-PP system (1.05 ppm). This result reveals that the methyl substituents affect the sensor’s sensitivity to nitrite, but that the location of them, whether blocking both preferential positions or not, does not change the LOD substantially. Therefore, the electrostatic interaction of nitrite to these sensor systems must be delocalized over the entirety of the pyrrole ring, and is not limited to a specific position on the pyrrole moiety.

These results highlight the unique characteristics of these small-molecule-aggregate-based systems. Here, the electrostatic interaction with nitrite to PP, 2,5-PP, and 2,4-PP indicates that the interaction is not site-specific, but does show slight deviations in the sensitivity. It also provides significant insight into the complexity of the conformation of all their aggregates in a solution. The size of the aggregates was shown to vary between all three systems as well as how many monomers are contained within the supramolecular structure. Interestingly, through these manipulations, it is revealed that even slight structural changes upon the addition of methyl groups can illustrate vast differences in their spectroscopic properties. While all solutions are yellow in an aqueous solution, upon the addition of nitrite, all three systems show various spectroscopic changes after interaction with the anion. For instance, both methylated sensors, 2,5-PP and 2,4-PP, do not exhibit colorimetric change, like that of PP. Instead, both show spectroscopic changes in the ultraviolet region of the electromagnetic spectrum, but still vary by 32 nm. This work highlights the intricate characteristics and properties of aggregate-based sensors to be used for anion sensing. Current work is underway to determine the long-term stability of these molecular aggregates.

### 2.8. Comparisons to Previously Employed Systems

The three systems reported herein provide new insight into anion sensing via molecular aggregation. Current EPA methods widely employed utilize methods of a nitrite analysis that are susceptible to interferences, have long wait times, and use hazardous equipment. However, methods to detect nitrite have reached detection limits as low as 1.2 ppb as per recent reviews from Wang et al. and Singh et al. [48,49]. All three systems, PP, 2,5-PP, and 2,4-PP, provide detection limits within the standard employed by the WHO and EPA. With a portable spectrometer, or lab-on-a-chip device, these systems are non-hazardous and can be easily integrated into in-field testing or sampling of contaminated waterways. To better commercialize and employ these sensors in the field, experimental studies are ongoing to further improve the sensitivity of these systems. Here, manipulation into the supramolecular aggregate conformation can provide valuable insight into the sensitivity of the aggregate due to a decrease in π-stacking between monomers, or by the potential structural manipulation of the pyridine moiety with electron-withdrawing and/or -donating groups. The three pyrrole–pyridine-based sensors reported illustrate the potential for non-hazardous, small-molecule anion sensors to replace current methods of analyses in the future.

## 3. Materials and Methods

### 3.1. Reagents

All materials were purchased commercially and used as received unless specified. The following materials were used: methylene chloride (Fisher (Waltham, MA USA), 99%), 2,4-dimethylpyrrole (Fisher, 99%), 2,5-dimethylpyrrole (Fisher, 99%), 4-chloropyridine hydrochloride (Sigma, 99%), hydrochloric acid (Fisher, 98%), sodium carbonate (Fisher, 99%), anhydrous sodium sulphate (Fisher, 99%), sodium sulfite (Fisher, 99%), sodium bisulfite (Fisher, 99%), sodium nitrate (Fisher, 99%), sodium nitrite (Fisher, 99%), sodium phosphate (Fisher, 99%), sodium chloride (Fisher, 99%), sodium fluoride (Fisher, 99%), sodium bromide (Fisher, 99%), potassium iodide (Fisher, 99%), chloroform (Fisher, 99%), acetonitrile (Fisher, 99%), acetone (Fisher, 99%), diethyl ether (Fisher, 99%), 0.01 M Pi phosphate buffer, and hexane (Fisher, 99%). Spectroscopy grade 18 MΩ water was used as a solvent unless otherwise specified.

### 3.2. Syntheses of 4-(Pyrrol-1-yl)pyridine and Derivatives

#### 3.2.1. Synthesis of 4-(Pyrrol-1-yl)pyridine (PP)

The synthesis of 4-(pyrrol-1-yl)pyridine was performed as previously described in detail [45]. All ^1^H, ^13^C NMR, and two-dimensional NMR (COSY, HSQC), followed the procedures previously reported [45].

#### 3.2.2. Synthesis of 4-(2,5-Dimethyl-pyrrol-1-yl)pyridine (2,5-PP)

Synthesis of 4-(2,5-dimethyl-pyrrol-1-yl)pyridine (2,5-PP) was completed using the photochemical procedure of 4-(pyrrol-1-yl)pyridine, with modifications [45]. The synthesis was carried out utilizing a 1:70 ratio of 4-chloropyridine hydrochloride (2.50 × 10^−^^4^ mol) and 2,5-dimethylpyrrole (0.0175 mol). In a 250 mL quartz round-bottom flask, 4-chloropyridine hydrochloride (0.0375 g) was dissolved in methylene chloride (100 mL). The resulting solution was stirred under nitrogen for 1 h. The 2,5-dimethylpyrrole (1.78 mL) was added, and the mixture was irradiated for six minutes under constant stirring and bubbling of nitrogen. The resulting solution appeared as orange in colour and continued to deepen throughout irradiation. The reaction was monitored via UV–visible spectroscopy until completion. The 1:70 ratio is a far contrast to our previous 4-(pyrrol-1-yl)pyridine system, employing a ratio of 1:32, but was found to be the most favourable. Here, excess pyrrole is required to avoid the formation of bipyridine and gave higher purity of the final product with excess reactant more readily removed. Upon reacting, a new visible band absorbance maximum appeared at 446 nm. The extraction process was identical to that of the 4-(pyrrol-1-yl)pyridine system, and is summarized as follows: a 30 mL 10% hydrochloric acid wash, the neutralization of the acid layer with sodium carbonate, and 30 mL dichloromethane was dried with solid anhydrous sodium sulphate, and concentrated to dryness to yield a red-brown oil with a yield up to 30%. The oil was stored at 277 K for longevity. The extraction process was employed to remove excess starting reagents and potential byproduct from the polymerization of 2,5-dimethylpyrrole.

#### 3.2.3. Synthesis of 4-(2,4-Dimethyl-pyrrol-1-yl)pyridine (2,4-PP)

The synthesis of 4-(2,4-dimethyl-pyrrol-1-yl)pyridine (2,4-PP) was carried out in an analogous procedure to that of 2,5-PP with modifications. A ratio of 1:3 was employed of 4-chloropyridine hydrochloride (2.50 × 10^−^^4^ mol) and 2,4-dimethylpyrrole (7.50 × 10^−^^4^ mol). Here, the irradiation time was 10 min, and the solution appeared as red-orange in colour. The reaction was again monitored via UV–visible spectroscopy until completion where a new visible band absorbance maximum appeared at 466 nm. The extraction process was analogous to the above compounds with the exception of additional hydrochloric acid wash volume to a total of 50 mL. The resulting brown oil (approximately 40% yield) was also stored at 277 K.

#### 3.2.4. Mass Spectroscopy and IR Spectroscopy of 2,5-PP and 2,4-PP

IR spectra of both products revealed all expected functional groups, and the mass spectra revealed the parent ions at m/z of 171 for both 4-(2,5-dimethyl-pyrrol-1-yl)pyridine and 4-(2,4-dimethyl-pyrrol-1-yl)pyridine. Analyses of both oil samples stored for upwards of 3 months revealed no structural degradation. This was measured via both NMR and UV–visible spectroscopy. No fluorescence was exhibited in either of the aqueous solutions of 2,5-PP or 2,4-PP.

### 3.3. Instrumentation and Procedures

All spectroscopic data were recorded at 293 K. UV–visible spectra were taken on a Shimadzu UV–visible spectrometer in spectroscopy grade 18 MΩ water, unless otherwise stated. Spectra were recorded in a 1.00 cm pathlength quartz cuvette and baselines were set with 18 MΩ water as a reference, unless indicated otherwise. A longer-pathlength cell, 10 cm, was employed to measure concentrations as low as 1 × 10^−^^5^ M. All aqueous solutions had a low pH (<3) upon dissolution: a drop of hydrochloric acid was added to facilitate dissolution. Freshly prepared solutions were used in all cases to avoid long-term degradation effects. Solutions were deoxygenated by nitrogen purging for one hour as a precaution, but showed no discernable differences from aerated solutions. All NMR data were recorded on a Bruker NEO 400 MHz spectrometer (Bremen, Germany) in 99.99% pure CDCl_3_, or 99% pure d-DMSO in high-precision 525-pp NMR tubes. Spectra obtained in CDCl_3_ and d-DMSO yielded clearer baselines and avoided distortions in spectra due to the suppression of water protons. Dynamic light scattering (DLS) data were obtained using a Malvern Zetasizer Lab, using 18 MΩ water as a solvent at 293 K in Malvern Zetasizer nano-series plastic cuvettes. The volumes used matched the minimum volume of the instrument, approximately 1 mL. The refractive index used was 1.59. Ten duplicates were obtained with an equilibration time of 20 s, using a side scatter angle of detection. Fluorescence spectroscopy was performed on a Horiba Jobin FluoroMax 3 fluorescence spectrometer (Edison, NJ, USA) with excitation wavelengths between 340 and 470 nm. Fluorescence cuvettes were employed, utilizing a 1 cm pathlength. FT-IR spectra were recorded using a Shimadzu FT-IR instrument (Portland, OR, USA), using a zinc selenide ATR crystal. LC-MS data were obtained on a Shimadzu LC-MS-2020 fitted with a quadrupole. The solutions used were 5.69 × 10^−^^4^ M and filtered through syringe filters after dissolution in LC-MS-grade water. A C18 column with an eluent of water (0.1% formic acid) and acetonitrile (0.1% formic acid) were both used and results were analyzed in the positive channel.

#### 3.3.1. Dynamic Light Scattering

Approximately 1 mL aqueous samples of 4-(2,5-dimethyl-pyrrol-1-yl)pyridine and 4-(2,4-dimethyl-pyrrol-1-yl)pyridine in pure water were inserted into a plastic cuvette, matching the required volume minimum of the instrument. All samples were filtered prior to analyses. Ten trials were recorded per sample, employing a refractive index of 1.59, equilibration time of 20 s, and side scatter angle of detection.

#### 3.3.2. Tyndall Effect

Approximately 4 mL aqueous samples of 2,5-PP and 2,4-PP were irradiated with light from a helium laser in a dark room to determine if light scattering was present. All samples were filtered prior to analyses.

### 3.4. Titrations with 1.00 × 10^−2^ M Sodium Nitrite (aq.)

#### 3.4.1. PP Solutions

Anion titrations with sodium nitrite were previously reported [45]. These methods were consistent with the other two molecules reported, vide infra.

##### 3.4.2. 2,5-PP Solutions

All aqueous solutions of 2,5-PP were recorded upon immediate dissolution in pure water. UV–visible spectra were recorded over the range of 200–800 nm. A sample of 1.00 × 10^−^^2^ M aqueous sodium nitrite was prepared in a 100.00 mL volumetric flask. Titrations of 10.00 µL (1.00 × 10^−^^2^ M) sodium nitrite were injected into 2.00 mL aqueous 2,5-PP via a micropipette. Solutions were prepared in acidic pH, generally with pH ranging from 2 to 4. A 0.01 M Pi phosphate buffer was also employed to investigate solutions in the pH range between 4 and 6. The resulting spectra were monitored via UV–visible spectroscopy upon the addition of nitrite; no colorimetric change was observed. Plots for the limit of detection determinations (LODs) used the starting absorbance at 309 nm of the sensor (A_sensor_), absorbance upon each successive addition of nitrite at 309 nm (A_nitrite_), maximum absorbance at 309 nm after all additions of nitrite (A_max_), and minimum absorbance at 309 nm (A_min_). Errors for the limit of detection values were obtained from the error in the slopes of measurements using the LINEST function across ten trials. Results are reported in both M and ppm units.

#### 3.4.3. 2,4-PP Solutions

All anion titrations to 2,4-PP solutions were consistent with those reported for 2,5-PP. No colorimetric change upon the addition of nitrite was observed. Solutions were prepared in acidic pH, generally with pH ranging from 2 to 4. A 0.01 M Pi phosphate buffer was also employed to investigate solutions in the pH range between 4 and 6. Plots for the LOD used the starting absorbance at 341 nm of the sensor (A_sensor_), absorbance upon each addition of nitrite at 341 nm (A_nitrite_), maximum absorbance at 341 nm after all additions of nitrite (A_max_), and minimum absorbance at 341 nm (A_min_). Errors were obtained from the errors in the slopes of ten trials using the LINEST function. Results are reported in both M and ppm units.

### 3.5. Competitive Anion Titrations

#### 3.5.1. 2,5-PP Solutions

Solutions of 2,5-PP of various concentrations were made in pure water. Competitive anion solutions were made in 1:50 ratios of 1.00 × 10^−^^2^ M nitrite to 0.50 M competing anion in 100.00 mL volumetric flasks using their sodium or potassium salts. Typically, 9.81 × 10^−^^4^ M solutions of 2,5-PP were titrated with four 1.00 µL injections for a total of four additions. Spectra upon each addition were monitored via UV–visible spectroscopy.

#### 3.5.2. 2,4-PP Solutions

Competitive anion titrations of 2,4-PP followed the procedure previously stated for the 2,5-PP system; see The Tyndall Effect of 2,5-PP, 2,4-PP, and PP Solutions Section.

## 4. Conclusions

To summarize, we have discovered and investigated a new chemodosimeter class for the electrostatic detection of nitrite with two structural modifications from our previous 4-(pyrrol-1-yl)pyridine sensor. By manipulating the original base structure of PP to produce the 2,5-PP and 2,4-PP derivatives, the sensitivity of the sensor to nitrite significantly changes. Here, the 2,5-PP sensor yields a limit of detection of 1.06 ppm to nitrite; similarly, the 2,4-PP system exhibits an LOD to nitrite of 1.05 ppm. Both systems are roughly three times less sensitive to nitrite than the original PP sensor (0.330 ppm) [45]. The presence of the electron-donating methyl groups on the pyrrole ring appear to cause slight repulsion to the nitrite ion due to the increased electron density of the aromatic ring. However, the position of these methyl groups on the ring does not show significant influence on the sensitivity to nitrite ions. They are also shown to have no effect on the selectivity of the sensor to nitrite ions, with all three systems remaining highly selective to nitrite even with fifty times more competing anions in the solution. In addition, the presence of these methyl substituents illustrates changes in aggregate size to that of the PP system. These methyl groups and their electron-donating capabilities could subsequently stabilize their supramolecular structure and size via the increased electron density of the π system, increasing the interaction of the π-stacking ability of the monomers to form the aggregate. With both the 2,5-PP and 2,4-PP systems showing LODs of roughly 1 ppm, these would meet the maximum standard of nitrite from the WHO and EPA (3.00 ppm and 1.00 ppm). The intricacies of these systems highlight the complexity of supramolecular aggregate species that undergo structural modifications to affect the sensitivity and mechanism of their anion-sensing capabilities.

## Figures and Tables

**Table 1:** Compiled DLS data of PP, 2,5-PP, and 2,4-PP.

Molecule	Concentration (M)	Absorbance at Absorbance Maximum	Size (nm)
PP ^a^	7.20 × 10^−3^	0.792	363
	6.50 × 10^−3^	0.715	249
	2.11 × 10^−3^	0.232	142
2,5-PP ^b^	2.81 × 10^−3^	0.890	295
	1.92 × 10^−3^	0.610	282
	7.29 × 10^−4^	0.232	262
2,4-PP ^c^	4.67 × 10^−3^	0.780	351
	3.29 × 10^−3^	0.550	296
	1.26 × 10^−3^	0.210	191

a: absorbance maximum at 463 nm. Data from Thomas et al. [45]; b: absorbance maximum at 446 nm; c: absorbance maximum at 466 nm.

**Table 2:** Limit of detection values for PP, 2,5-PP, and 2,4-PP systems towards nitrite ion.

Molecule	LOD (ppm)	Error	Absorbance Maximum Used for Calculation (nm)
PP ^a^	0.330	±6%	509
2,5-PP	1.06	±5%	309
2,4-PP	1.05	±4%	341

a: data reported previously; see [45].

## Data Availability

Additional raw data can be obtained through contact with the corresponding author.

## Supplementary Materials

The following supporting information can be downloaded at: https://www.mdpi.com/article/10.3390/molecules29235692/s1, Figure S1: ^1^H NMR of 4-(2,5-dimethyl-pyrrol-1-yl)pyridine (30 mM) in CDCl_3_. (A) Full spectrum. (B) Aromatic region; Figure S2: ^13^C NMR of 4-(2,5-dimethyl-pyrrol-1-yl)pyridine in CDCl_3_; Figure S3: COSY NMR of 4-(2,5-dimethyl-pyrrol-1-yl)pyridine in CDCl3; Figure S4: HSQC NMR of 4-(2,5-dimethyl-pyrrol-1-yl)pyridine in CDCl_3_; Figure S5: ^1^H NMR of 90 mM 4-(2,5-dimethyl-pyrrol-1-yl)pyridine in CDCl_3_. (A) Full spectrum. (B) Aromatic region; Figure S6: ^1^H NMR of 120 mM 4-(2,5-dimethyl-pyrrol-1-yl)pyridine in CDCl_3_. (A) Full spectrum. (B) Aromatic region; Figure S7: Comparison of ^1^H NMR of 30 mM (blue), 90 mM (red), and 120 mM (green) of 4-(2,5-dimethyl-pyrrol-1-yl)pyridine in CDCl_3_; Figure S8: ^1^H NMR of 4-(2,5-dimethyl-pyrrol-1-yl)pyridine (30 mM) in d-DMSO. (A) Full spectrum. (B) Aromatic region; Figure S9: ^1^H NMR of 4-(2,5-dimethyl-pyrrol-1-yl)pyridine (90 mM) in d-DMSO. (A) Full spectrum. (B) Aromatic region; Figure S10: ^1^H NMR of 4-(2,5-dimethyl-pyrrol-1-yl)pyridine (120 mM) in d-DMSO. (A) Full spectrum. (B) Aromatic region; Figure S11: Comparison of ^1^H NMR of 30 mM (blue) 4-(2,5-dimethyl-pyrrol-1-yl)pyridine in CDCl_3_ and 120 mM in d-DMSO in aromatic region; Figure S12: ^1^H NMR of 4-(2,5-dimethyl-pyrrol-1-yl)pyridine with sodium nitrite in d-DMSO/H_2_O. (A) Full spectrum. (B) Aromatic region; Figure S13: COSY NMR of 4-(2,5-dimethyl-pyrrol-1-yl)pyridine with sodium nitrite in d-DMSO/H_2_O; Figure S14: ^1^H NMR of 4-(2,4-dimethyl-pyrrol-1-yl)pyridine (30 mM) in CDCl_3_. (A) Full spectrum. (B) Aromatic region; Figure S15: ^13^C NMR of 4-(2,4-dimethyl-pyrrol-1-yl)pyridine in CDCl_3_; Figure S16: COSY NMR of 4-(2,4-dimethyl-pyrrol-1-yl)pyridine (30 mM) in CDCl_3_. (A) Full spectrum. (B) Aromatic region; Figure S17: HSQC NMR of 4-(2,4-dimethyl-pyrrol-1-yl)pyridine (30 mM) in CDCl_3_; Figure S18: ^1^H NMR of 4-(2,4-dimethyl-pyrrol-1-yl)pyridine (90 mM) in CDCl_3_. (A) Full spectrum. (B) Aromatic region; Figure S19: ^1^H NMR of 4-(2,4-dimethyl-pyrrol-1-yl)pyridine (120 mM) in CDCl_3_. (A) Full spectrum. (B) Aromatic region; Figure S20: Comparison of 30 mM (blue), 90 mM (red), and 120 mM green ^1^H NMR spectra of 4-(2,4-dimethyl-pyrrol-1-yl)pyridine in CDCl_3_; Figure S21: ^1^H NMR of 4-(2,4-dimethyl-pyrrol-1-yl)pyridine (30 mM) in d-DMSO. (A) Full spectrum. (B) Aromatic region; Figure S22: ^1^H NMR of 4-(2,4-dimethyl-pyrrol-1-yl)pyridine (90 mM) in d-DMSO. (A) Full spectrum. (B) Aromatic region; Figure S23: ^1^H NMR of 4-(2,4-dimethyl-pyrrol-1-yl)pyridine (120 mM) in d-DMSO. (A) Full spectrum. (B) Aromatic region; Figure S24: Comparison of 30 mM 4-(2,4-dimethyl-pyrrol-1-yl)pyridine in CDCl_3_ (blue) and 120 mM 4-(2,4-dimethyl-pyrrol-1-yl)pyridine (red) in d-DMSO in aromatic region; Figure S25: ^1^H NMR of 4-(2,4-dimethyl-pyrrol-1-yl)pyridine with sodium nitrite in d-DMSO. (A) Full spectrum. (B) Aromatic region; Figure S26: COSY NMR of 4-(2,4-dimethyl-pyrrol-1-yl)pyridine with sodium nitrite in d-DMSO.
