# Supplementary material for: An Investigation into Anion Sensing of the Molecular Aggregate of 4-(Pyrrol-1-yl)pyridine and Its Derivatives
Source: Molecules. 2024 Dec 2;29(23):5692. doi: 10.3390/molecules29235692 (PMC11643978; doi:10.3390/molecules29235692)

A class of two methyl substituted 4(pyrrol-1-yl)pyridine based chemodosimeters for detection of nitrite ion in aqueous solution

Mallory E Thomas, Lynn D Schmitt, and Alistair J Lees

Supporting Information

S1: ^{1}H NMR of 4-(2,5-dimethyl-pyrrol-1-yl)pyridine (30 mM) in $CDCl_3$. (A) Full spectrum (B) Aromatic region

(A)

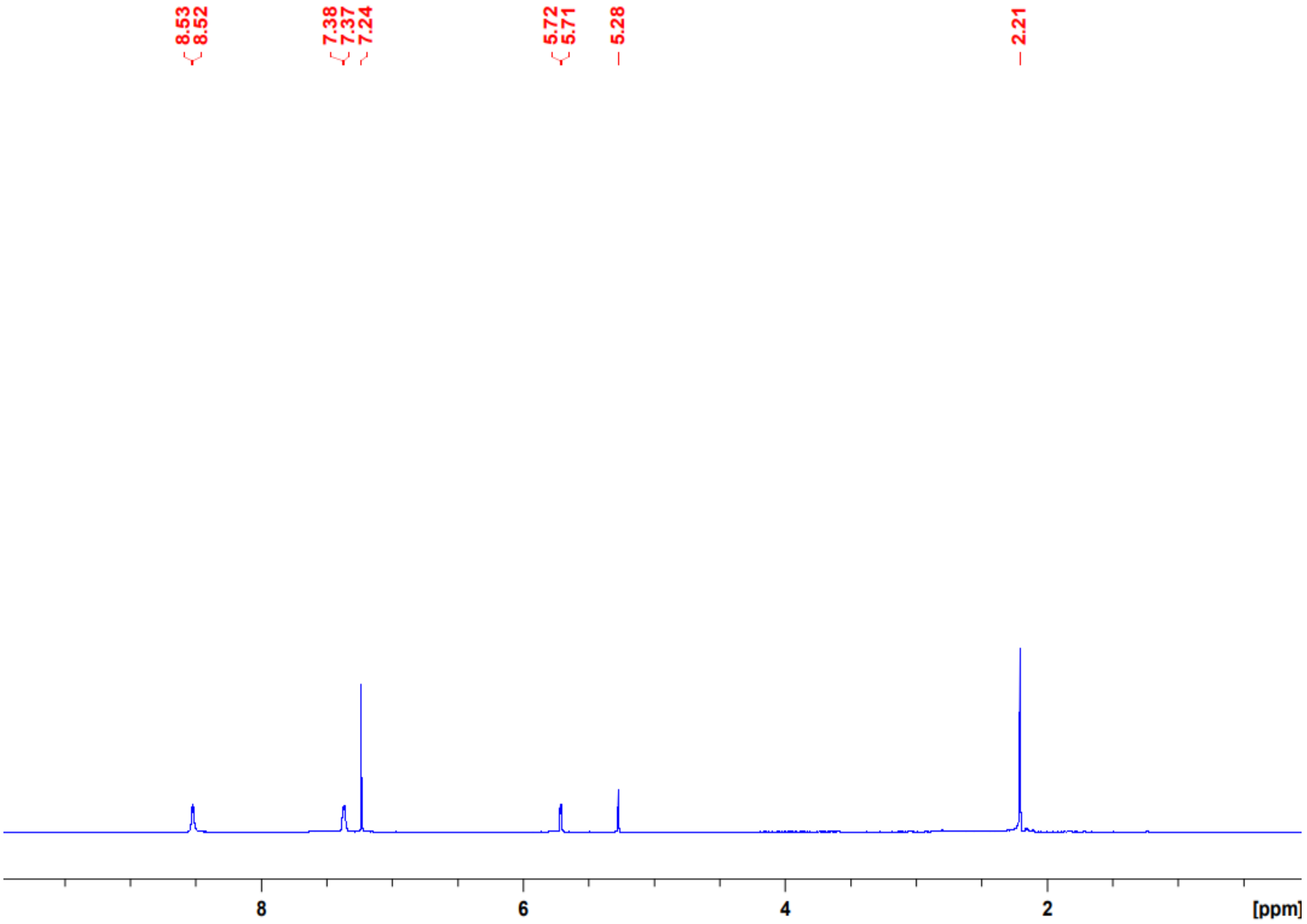

(B) Aromatic region

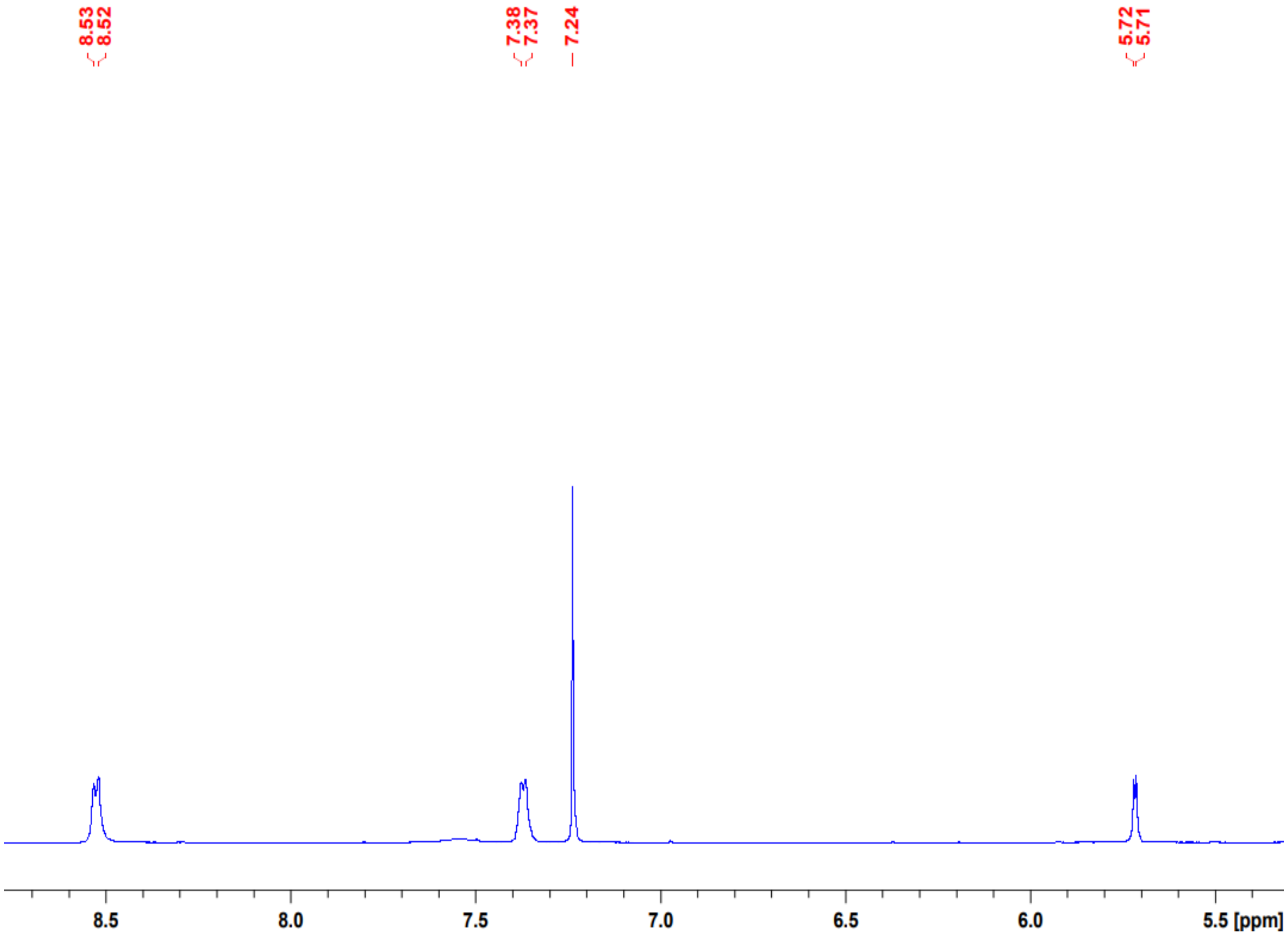

S2: ^{13}C NMR of 4-(2,5-dimethyl-pyrrol-1-yl)pyridine in $CDCl_3$. Peaks at 206.96 and 30.94 ppm are indicative of excess acetone solvent used for cleaning, and do not affect the spectrum of the molecule.

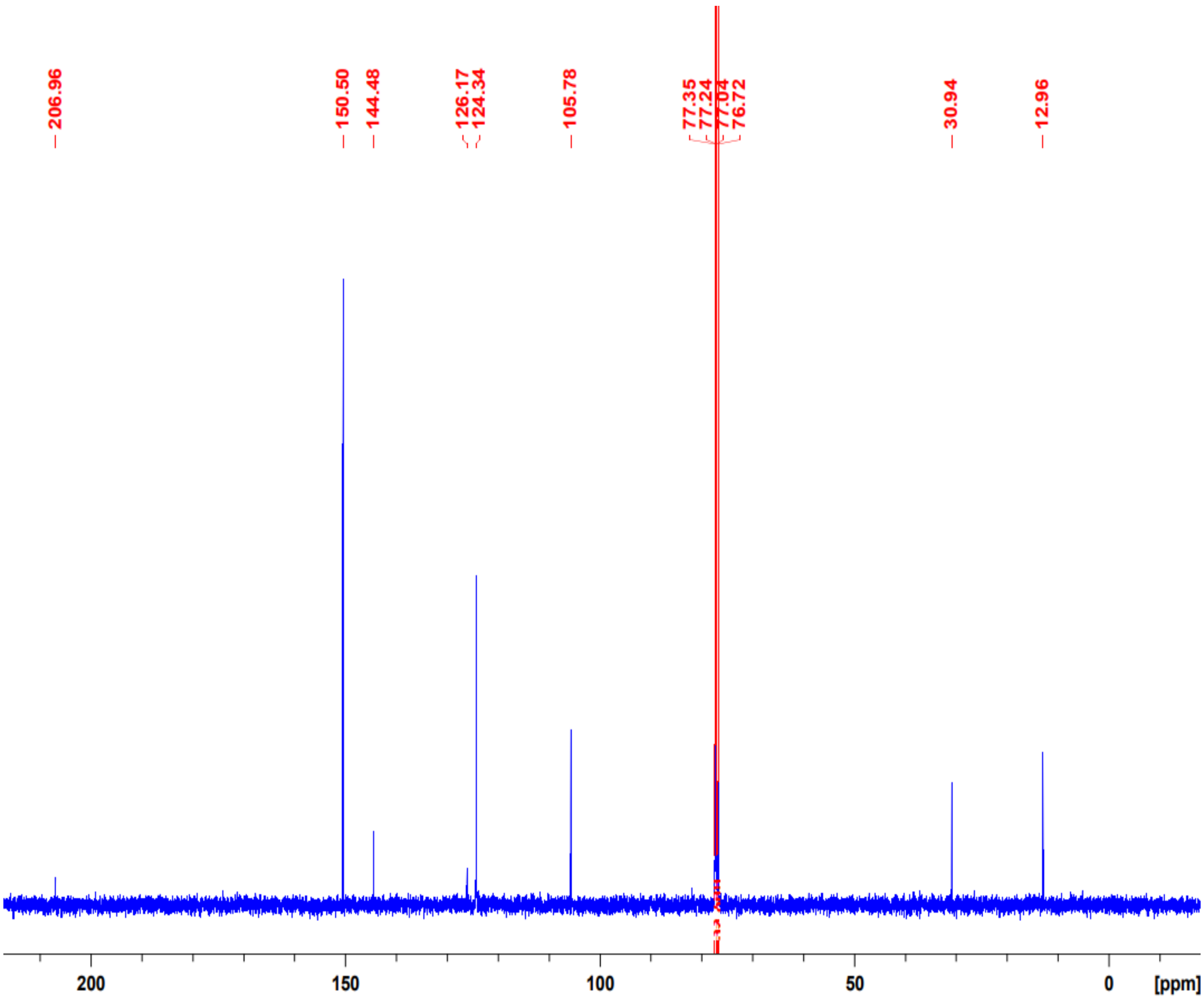

S3: COSY NMR of 4-(2,5-dimethyl-pyrrol-1-yl)pyridine in $CDCl_3$.

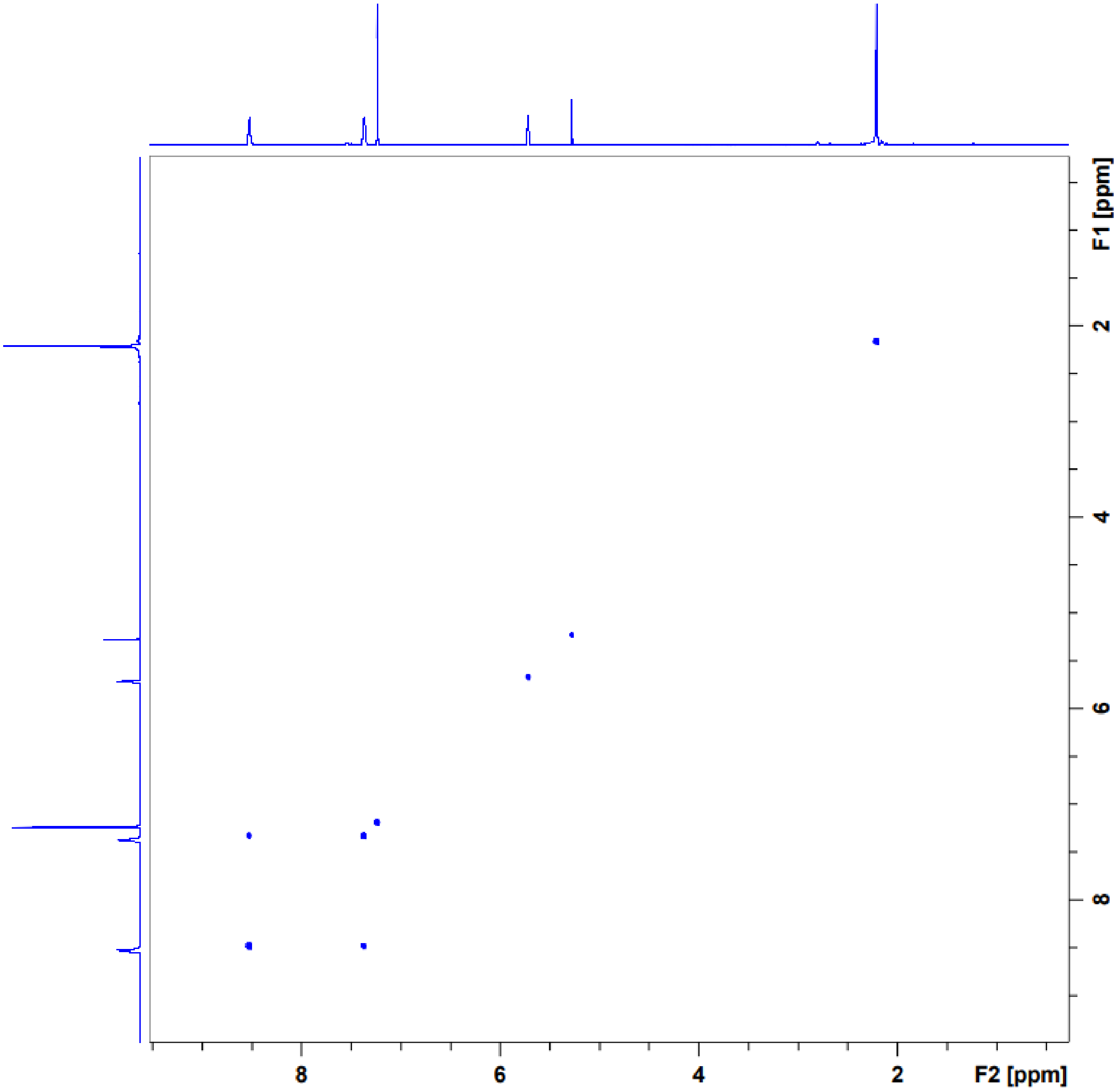

S4: HSQC NMR of 4-(2,5-dimethyl-pyrrol-1-yl)pyridine in $CDCl_3$.

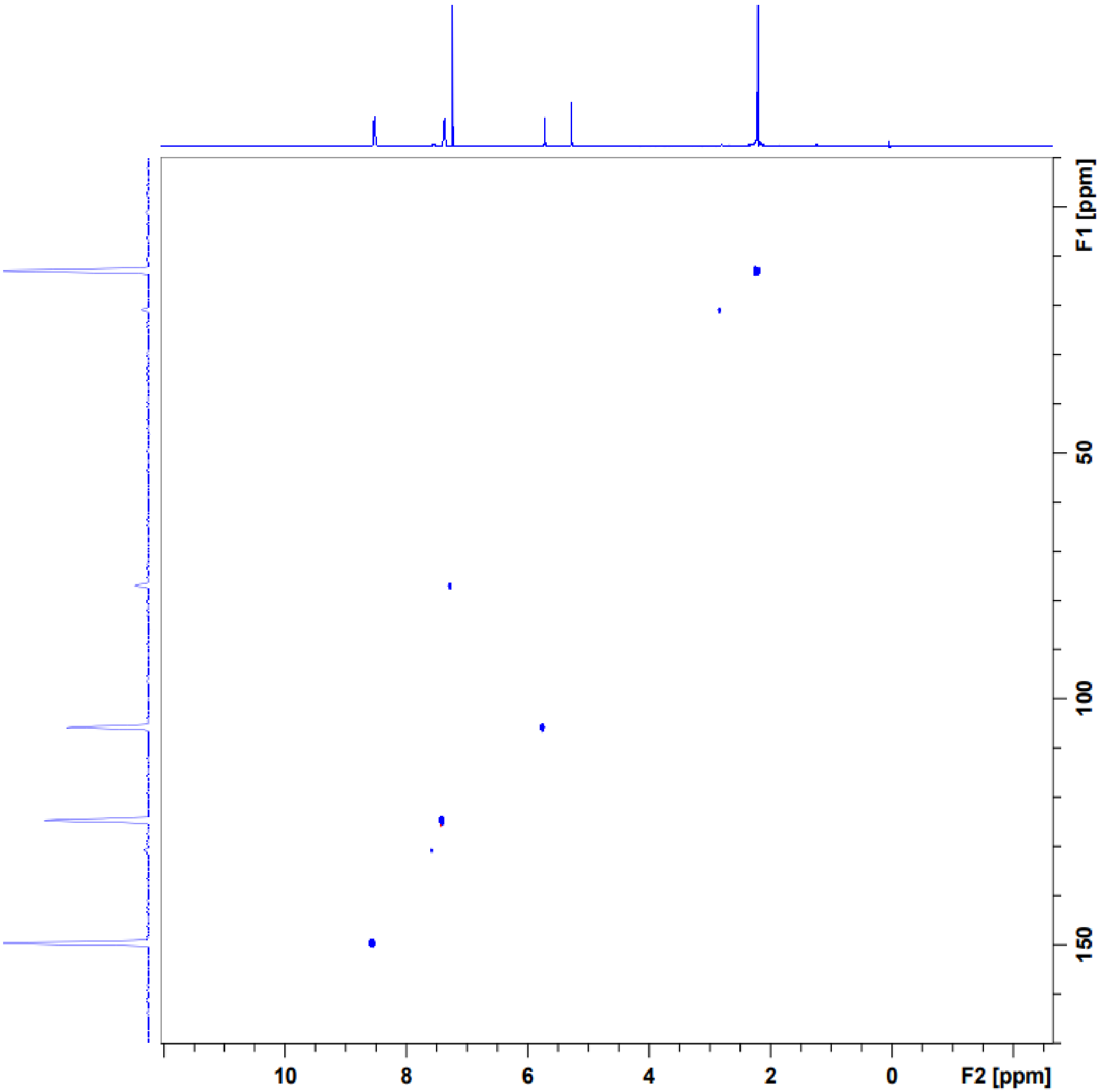

S5: ^{1}H NMR of 90 mM 4-(2,5-dimethyl-pyrrol-1-yl)pyridine in $CDCl_3$. (A) Full spectrum (B) Aromatic region. Peak at 5.28 ppm is indicative of excess dichloromethane solvent.

(A)

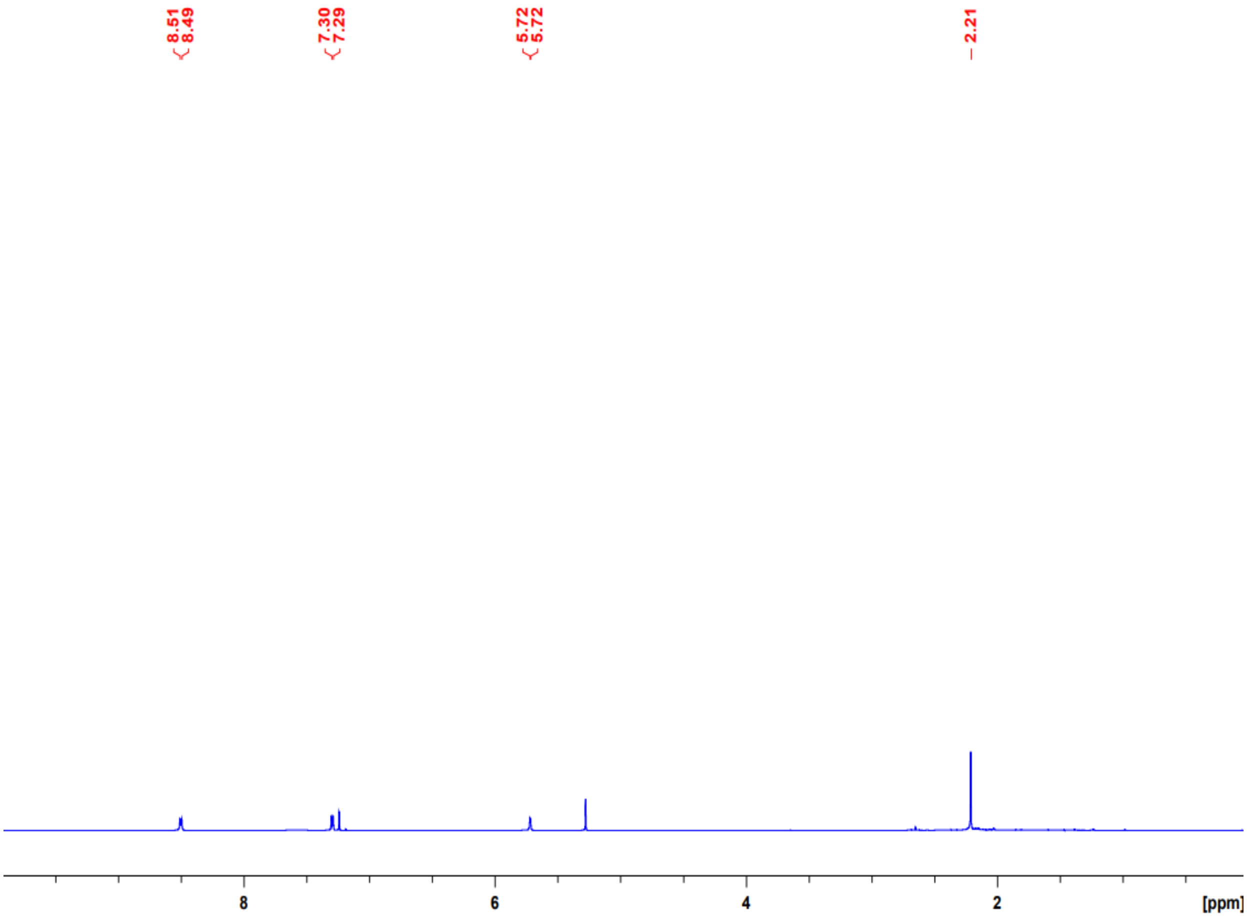

(B)

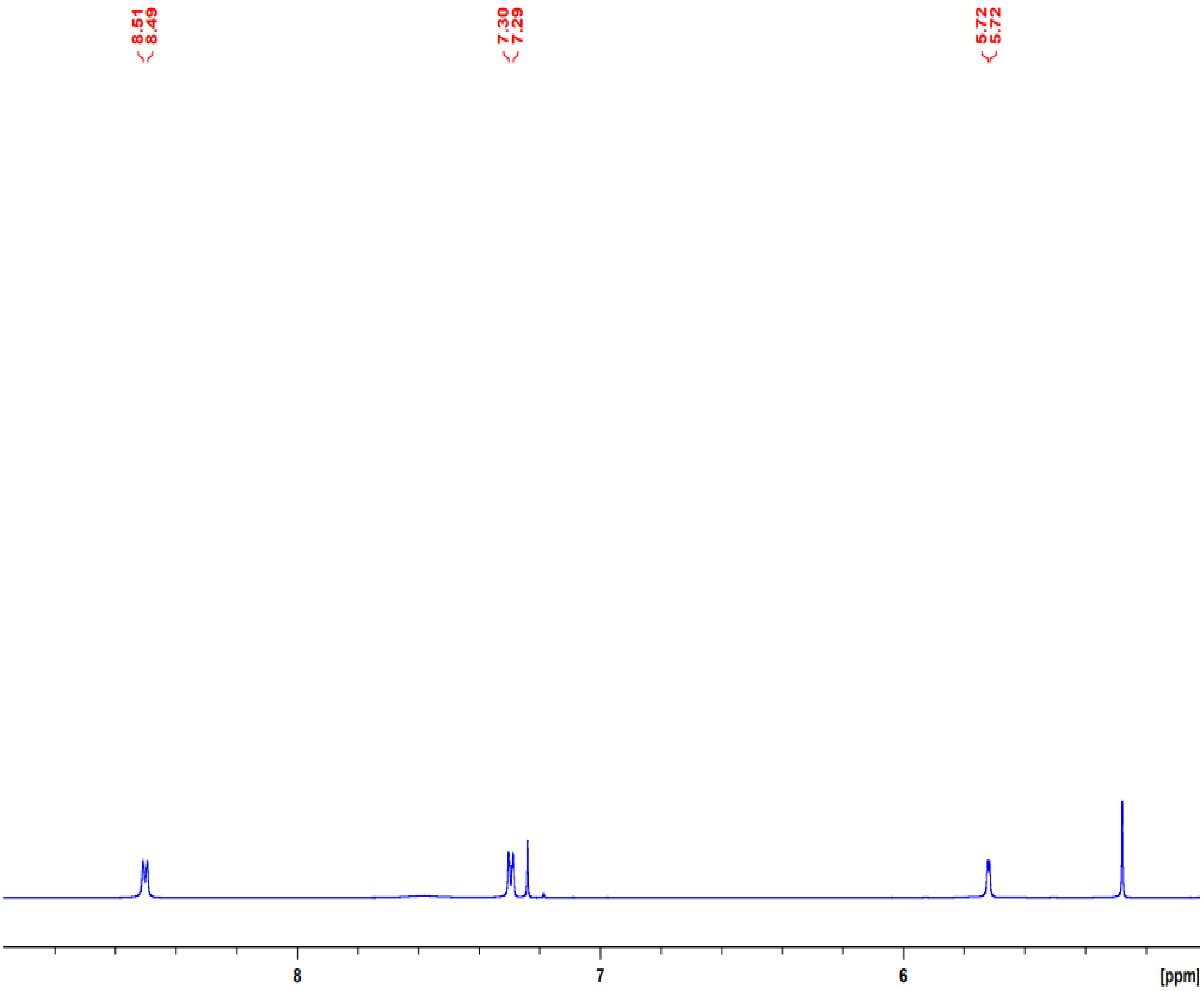

S6: ^{1}H NMR of 120 mM 4-(2,5-dimethyl-pyrrol-1-yl)pyridine in $CDCl_3$. (A) Full spectrum (B) Aromatic region. Peak at 5.28 ppm is indicative of excess dichloromethane solvent.

(A)

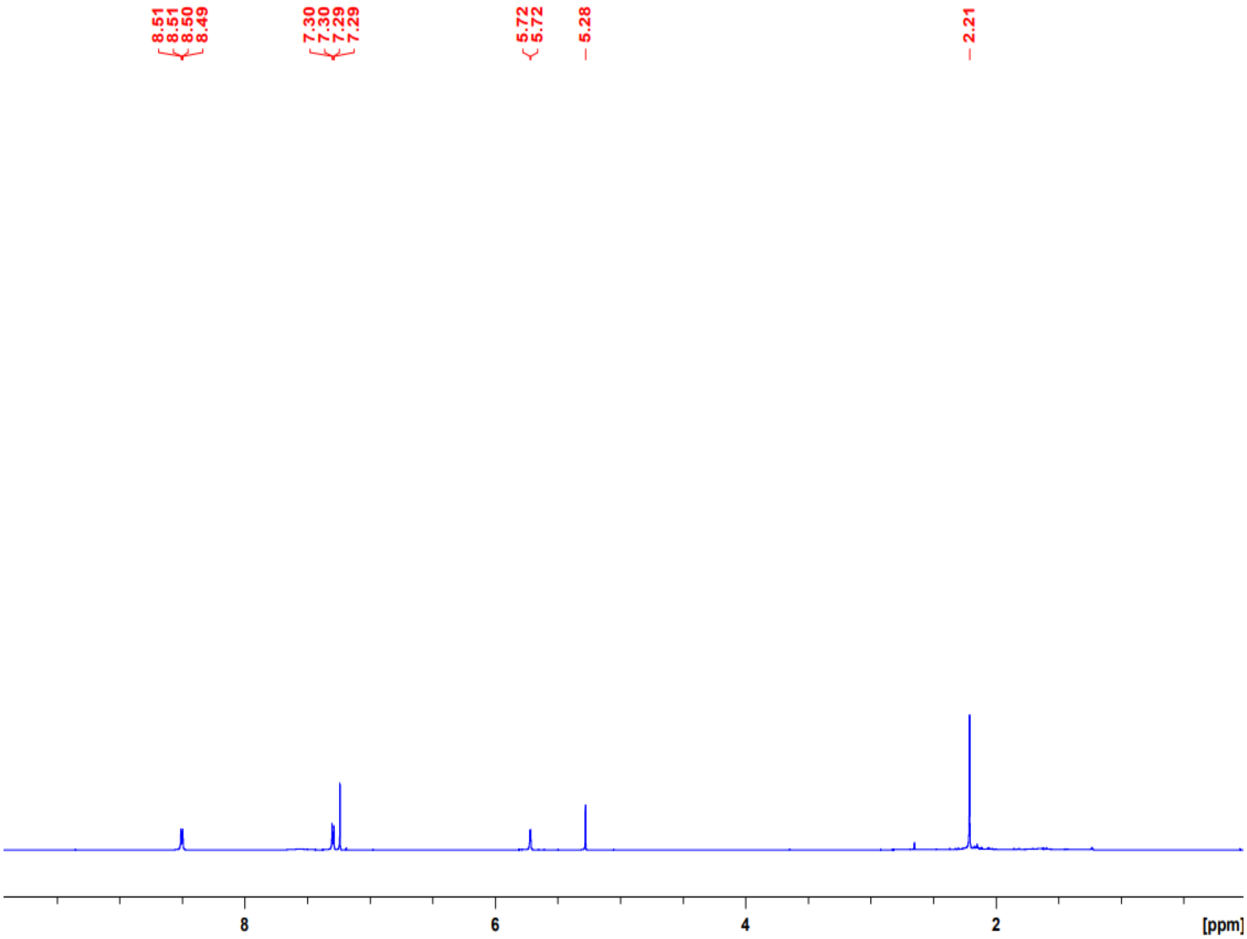

(B)

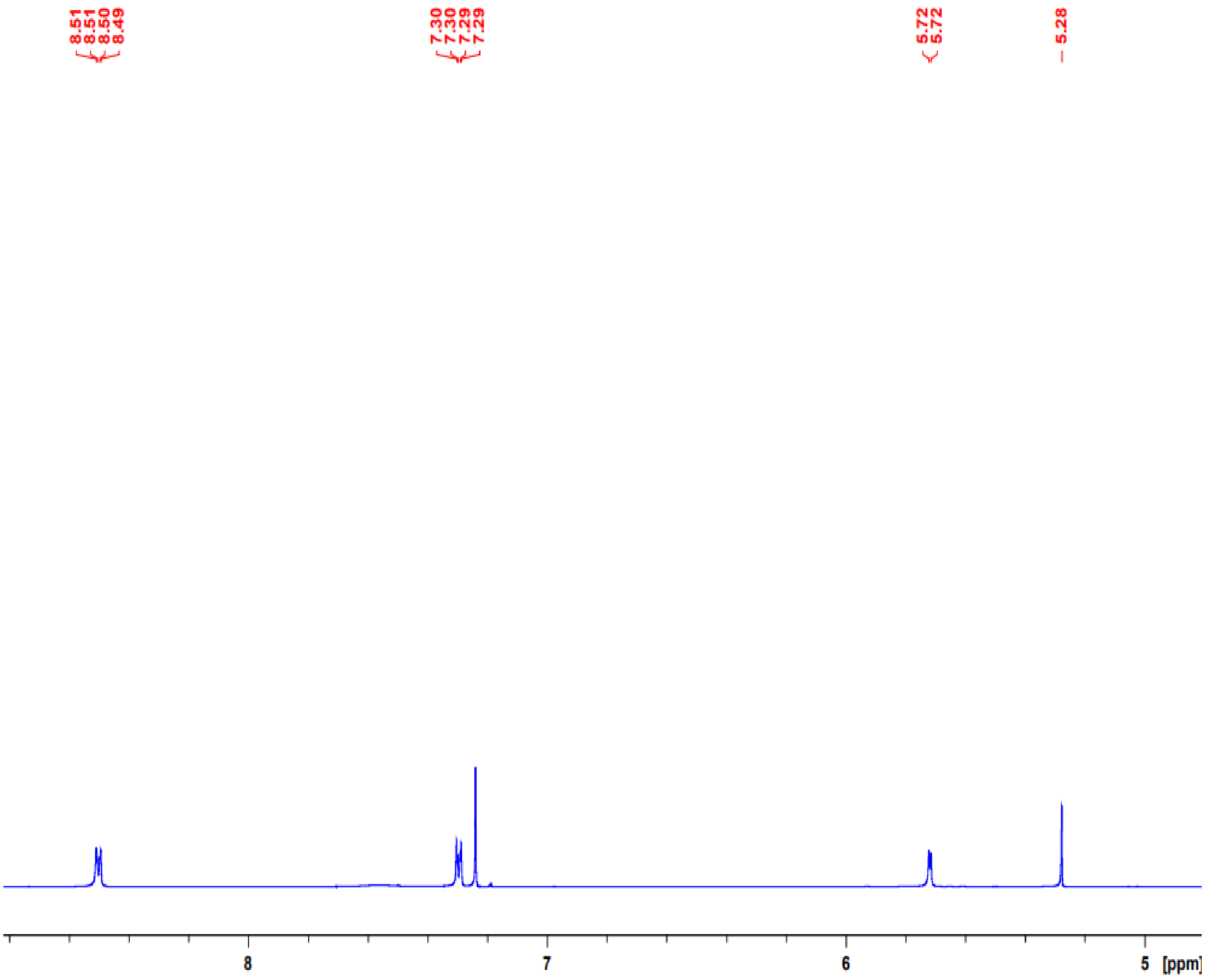

S7: Comparison of ^{1}H NMR of 30 mM (blue) 90 mM (red) and 120 mM (green) of 4-(2,5-dimethyl-pyrrol-1-yl)pyridine in $CDCl_3$.

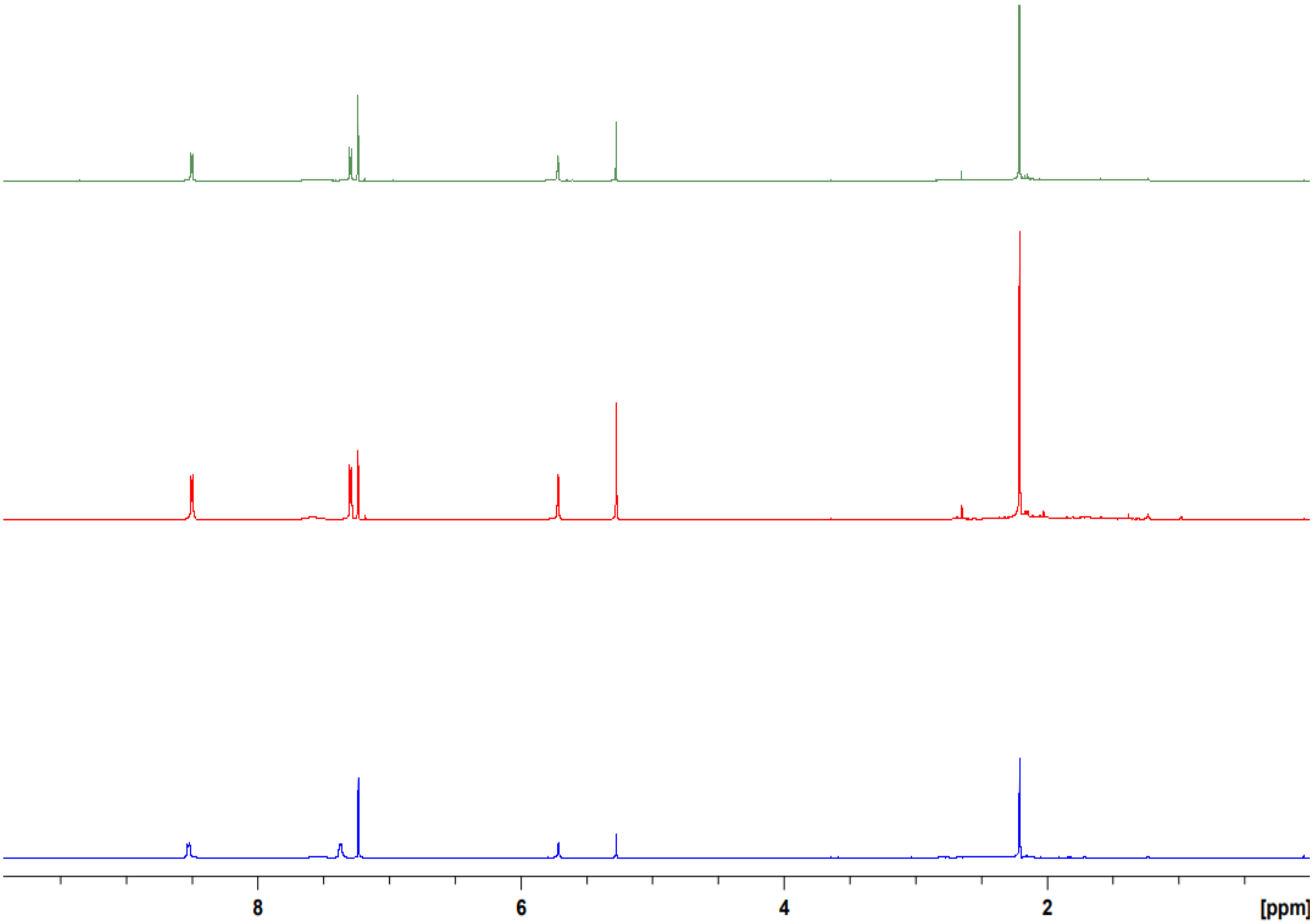

S8: ^{1}H NMR of 4-(2,5-dimethyl-pyrrol-1-yl)pyridine (30 mM) in d-DMSO. The peak at 5.75 ppm is indicative of leftover dichloromethane solvent and 3.32 ppm is indicative of water. Both trace molecules do not affect the spectrum of the sensor. (A) Full spectrum (B) Aromatic region

(A)

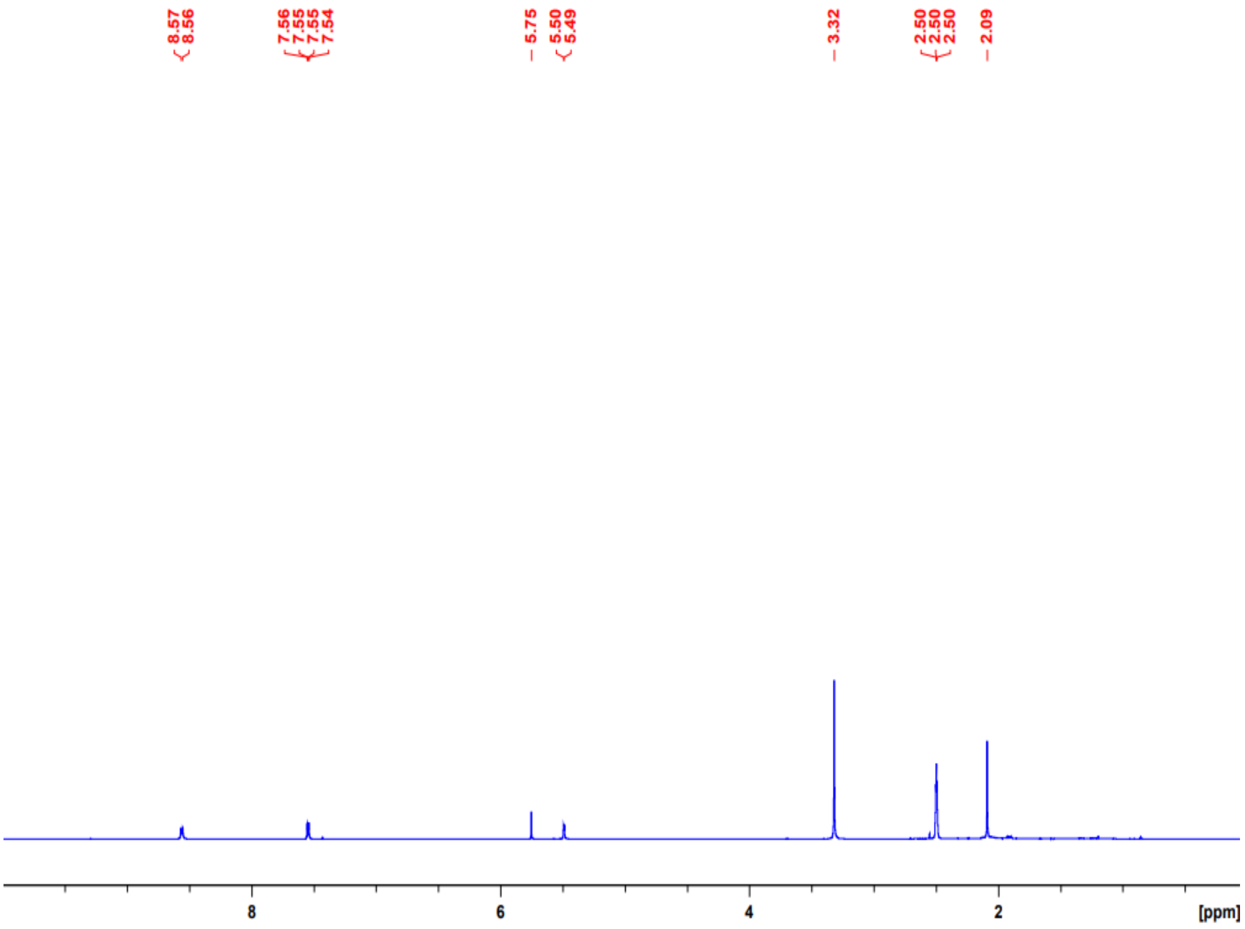

(B)

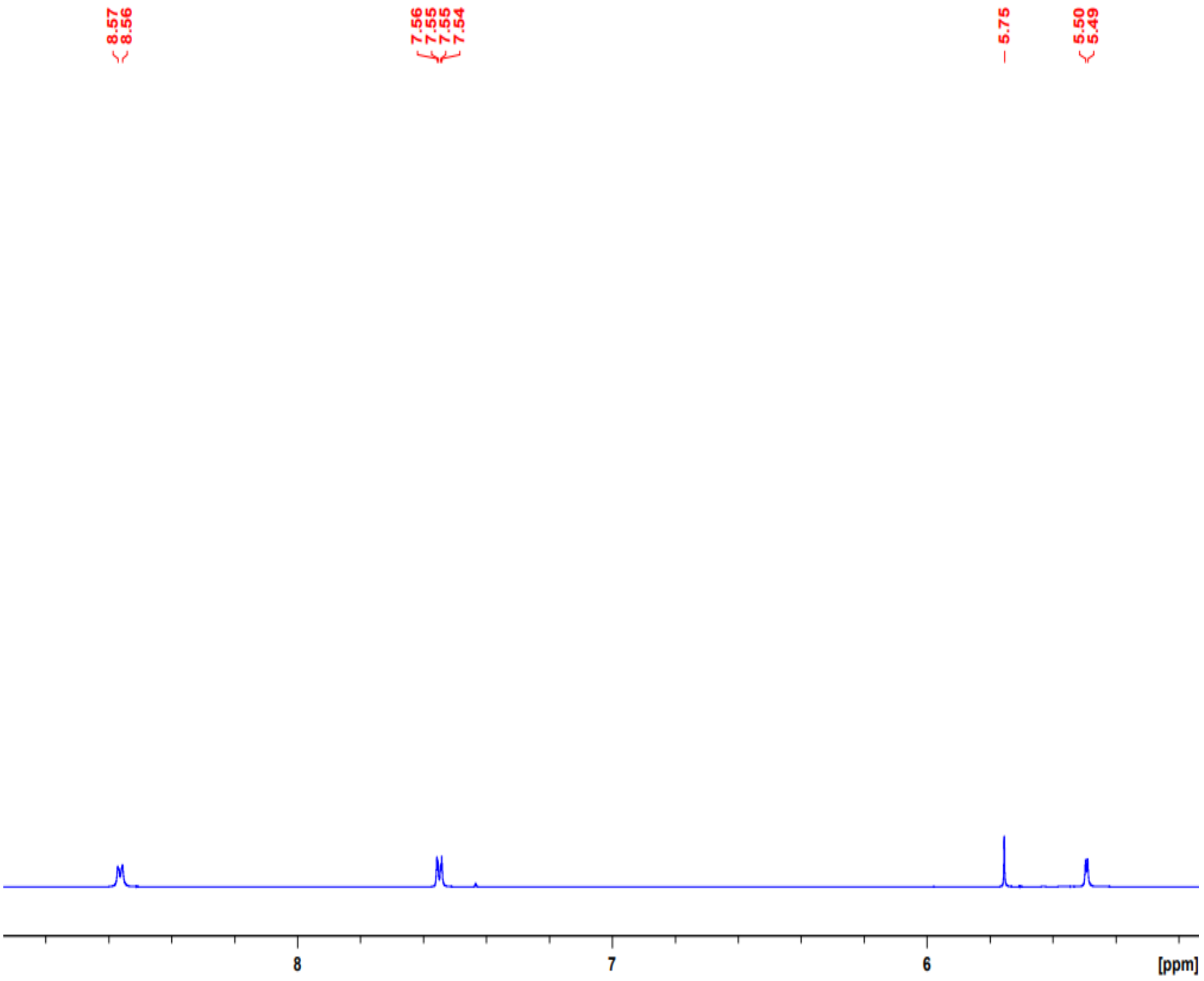

S9: ^1H NMR of 4-(2,5-dimethyl-pyrrol-1-yl)pyridine (90 mM) in d-DMSO. The peak at 5.75 ppm is indicative of leftover dichloromethane solvent and 3.32 ppm is indicative of water. Both trace molecules do not affect the spectrum of the molecule. (A) Full spectrum (B) Aromatic region

(A)

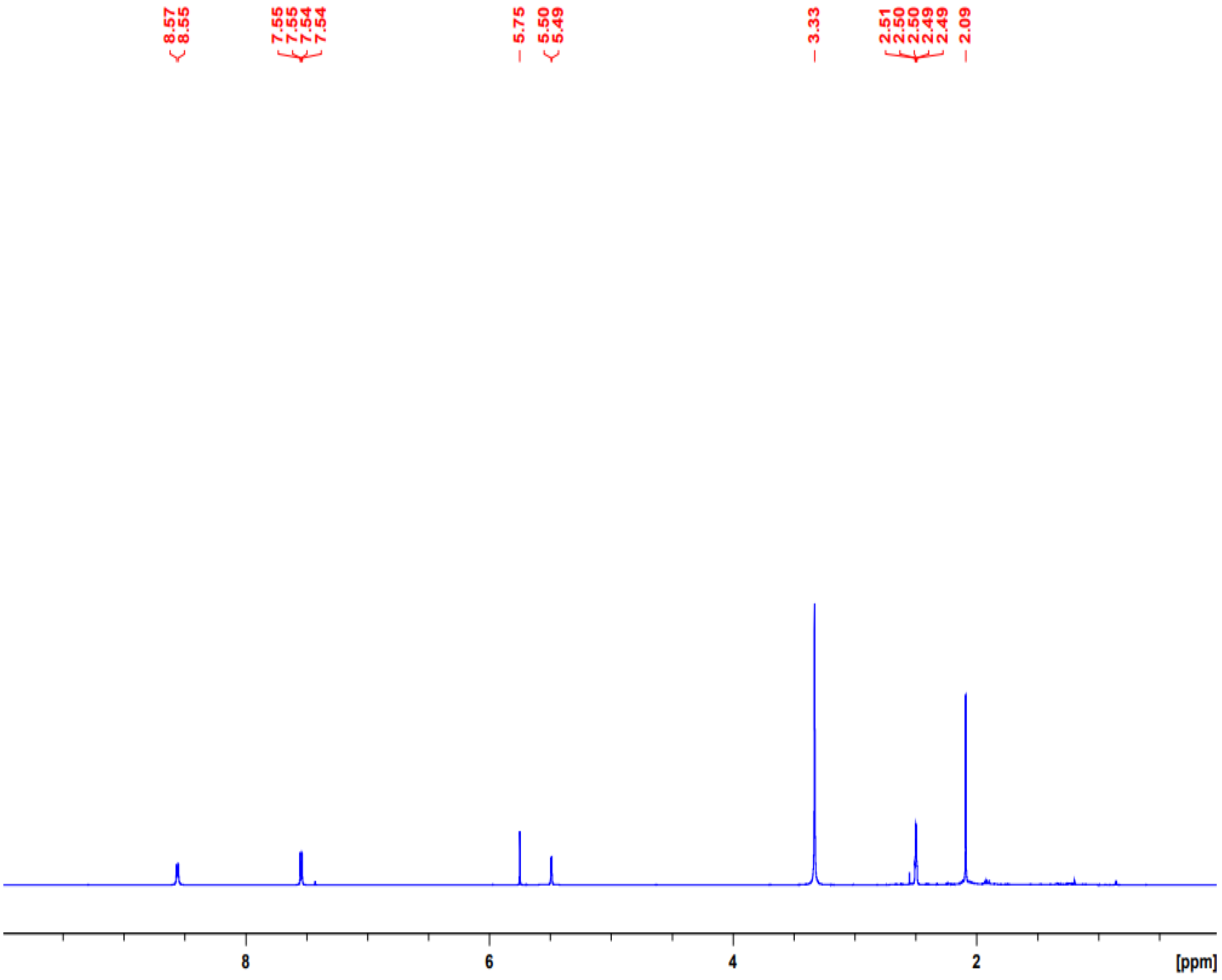

(B)

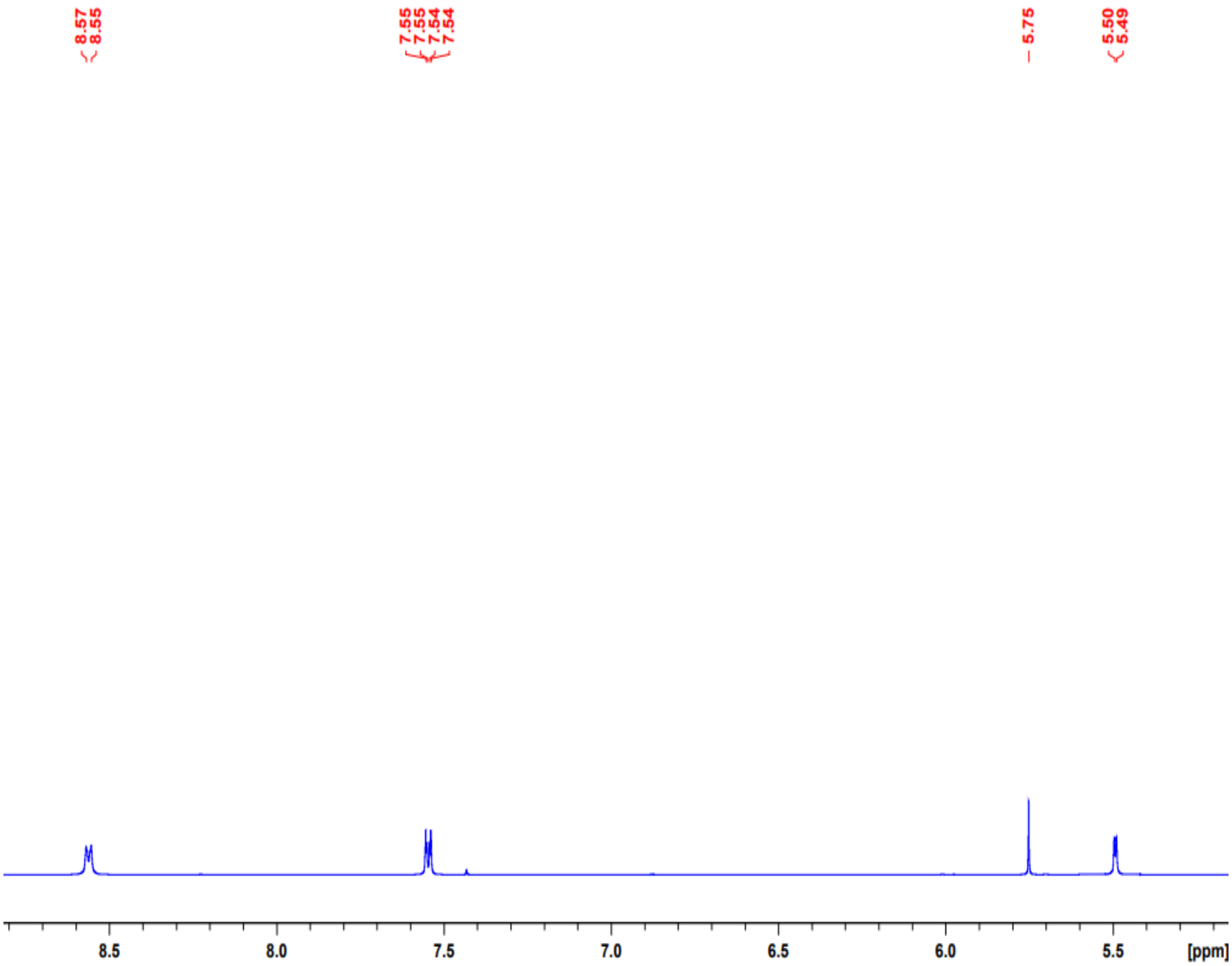

S10: ^{1}H NMR of 4-(2,5-dimethyl-pyrrol-1-yl)pyridine (120 mM) in d-DMSO. The peak at 5.75 ppm is indicative of leftover dichloromethane solvent and 3.32 ppm is indicative of water. Both trace molecules do not affect the spectrum of the molecule. (A) Full spectrum (B) Aromatic region

(A)

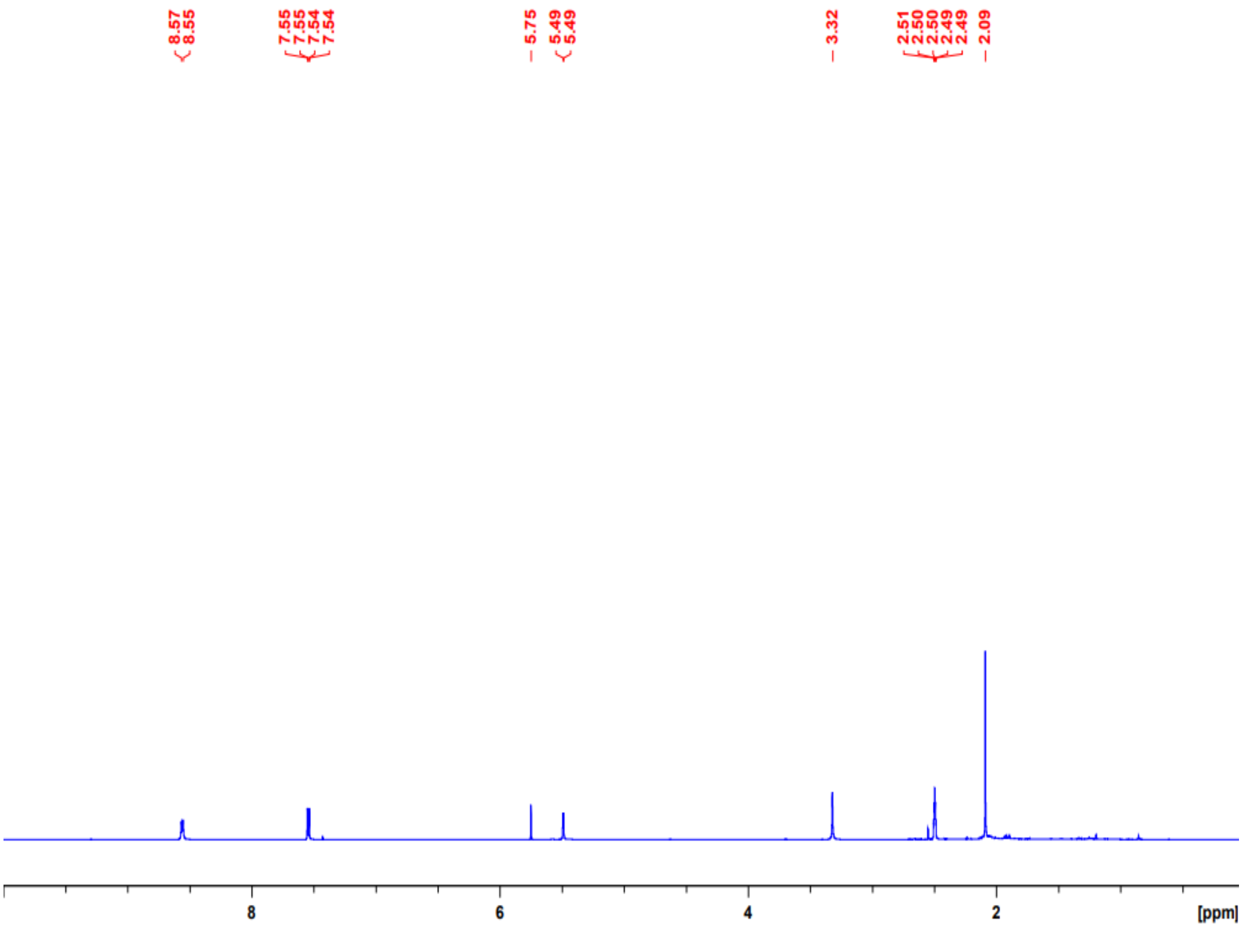

(B)

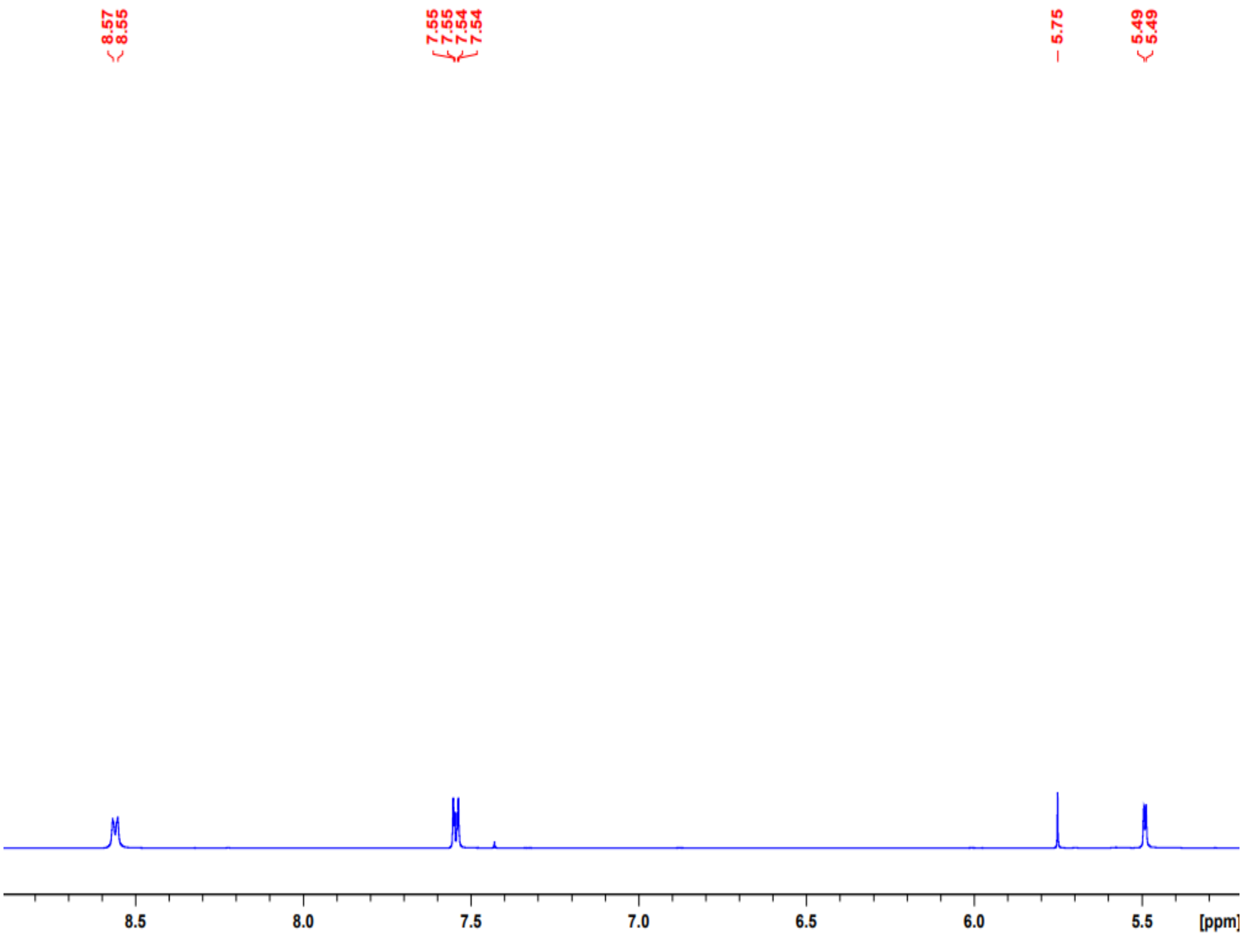

S11: Comparison of ^{1}H NMR of 30 mM (blue) 4-(2,5-dimethyl-pyrrol-1-yl)pyridine in $CDCl_3$ and 120 mM 4-(2,5-dimethyl-pyrrol-1-yl)pyridine in d-DMSO (red) in the aromatic region.

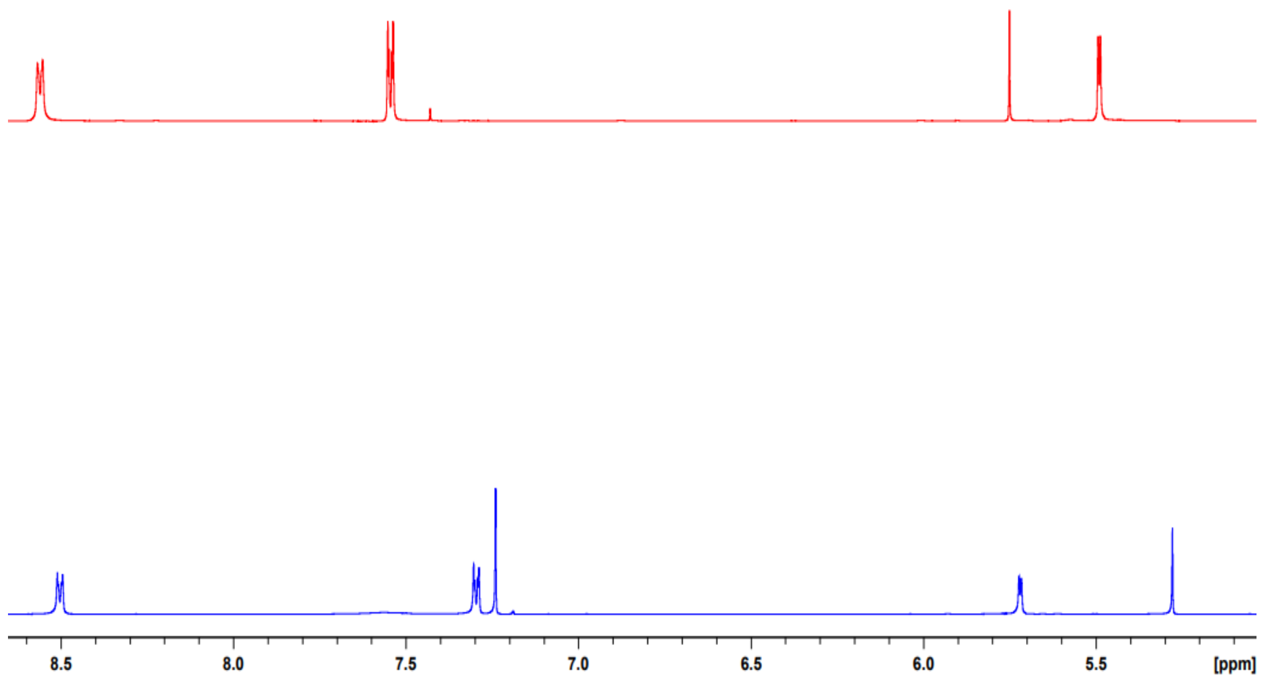

S12: ^{1}H NMR of 4-(2,5-dimethyl-pyrrol-1-yl)pyridine with sodium nitrite in d-DMSO/H_2O. The peak at 5.75 ppm is indicative of leftover dichloromethane, and does not affect the spectrum of the molecule. (A) Full spectrum (B) Aromatic region

(A)

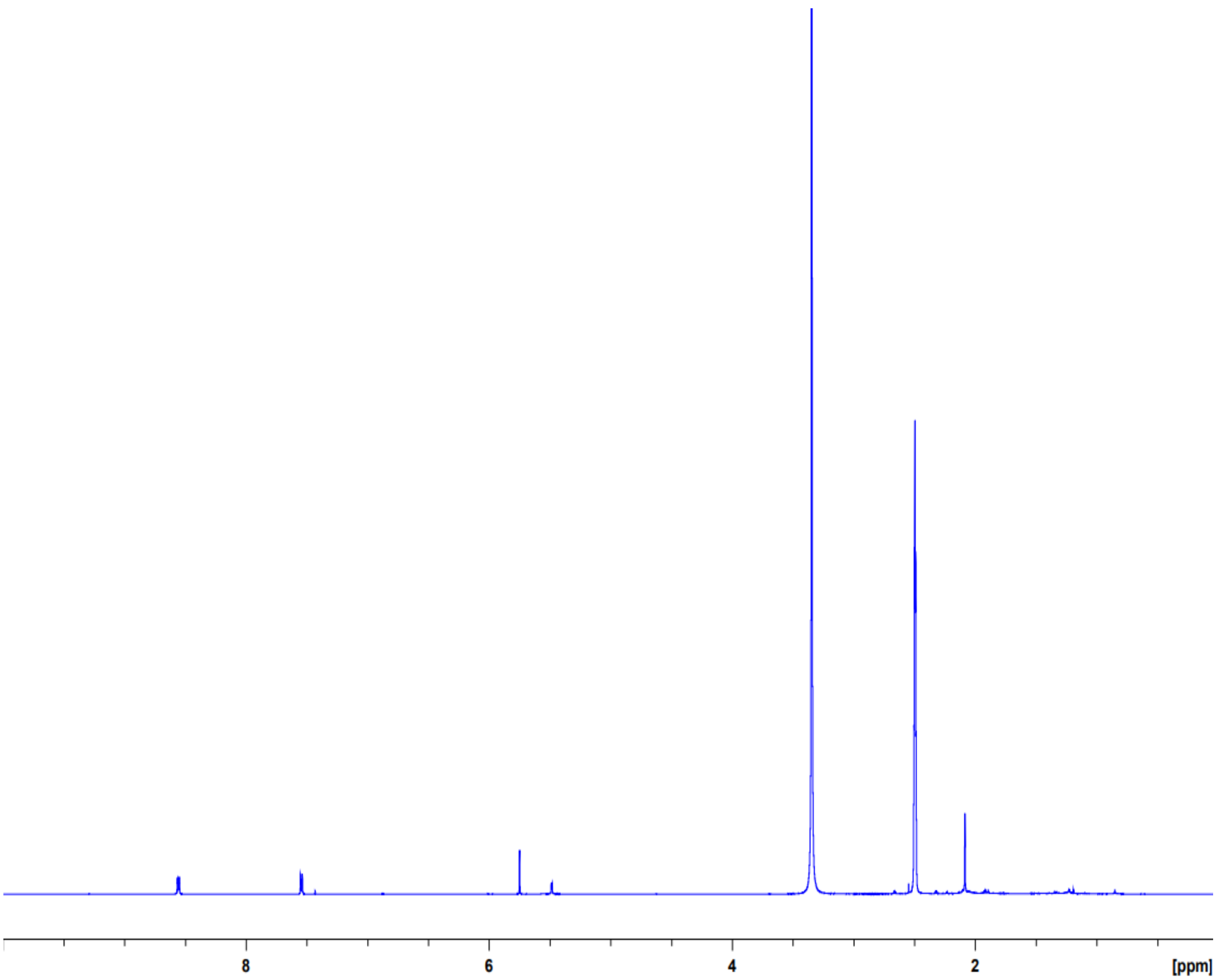

(B)

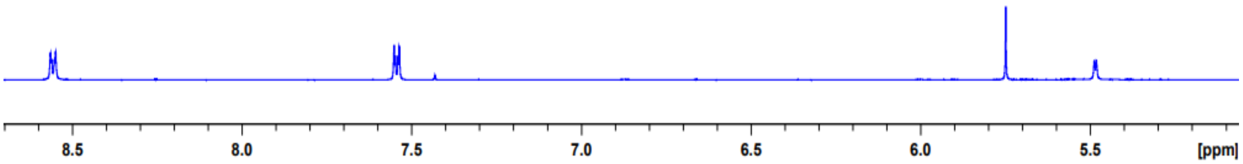

S13: COSY NMR of 4-(2,5-dimethyl-pyrrol-1-yl)pyridine with sodium nitrite in d-DMSO/H_2O.

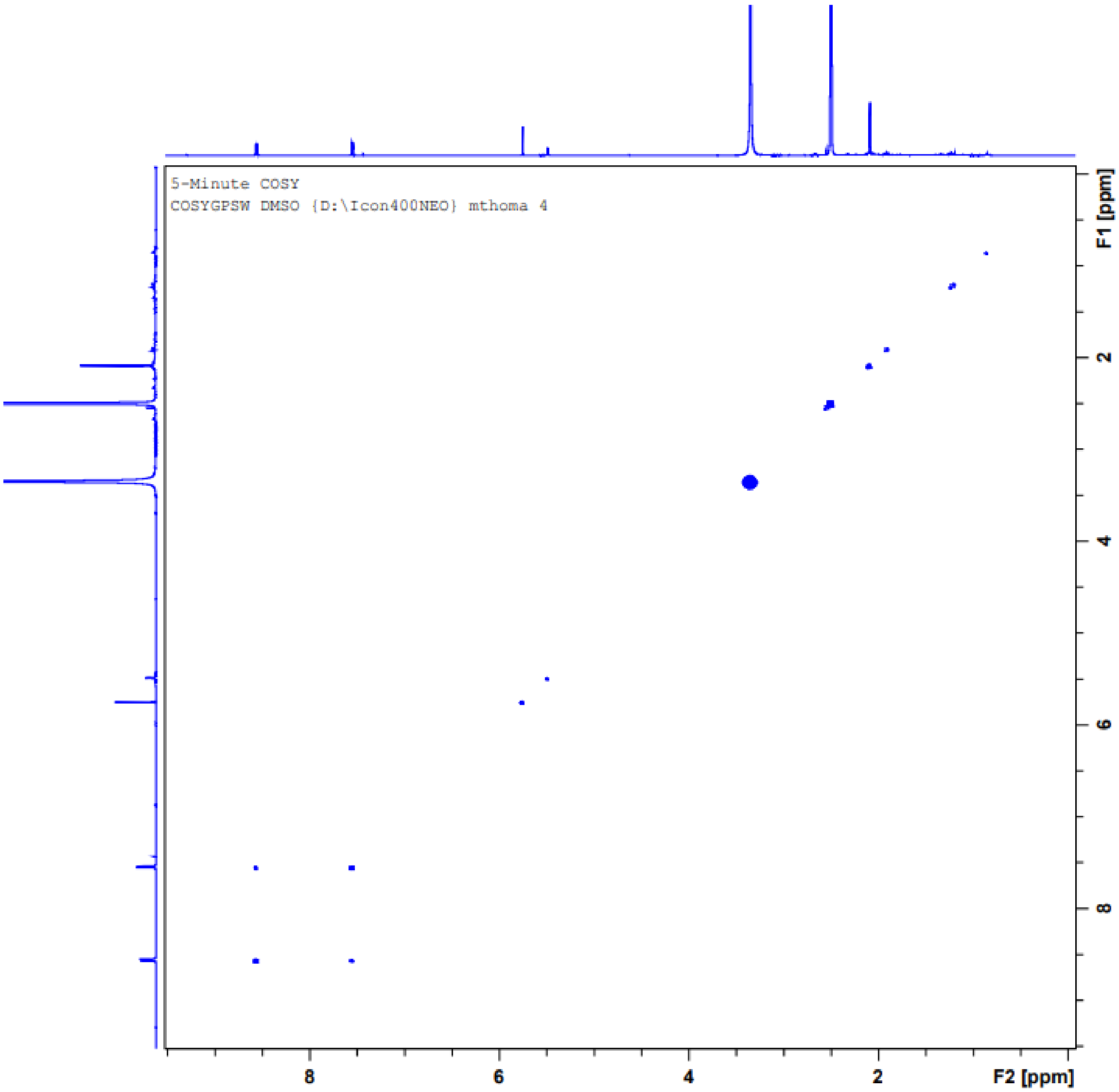

S14: ^1H NMR of 4-(2,4-dimethyl-pyrrol-1-yl)pyridine (30 mM) in $CDCl_3$. (A) Full spectrum (B) Aromatic region

(A)

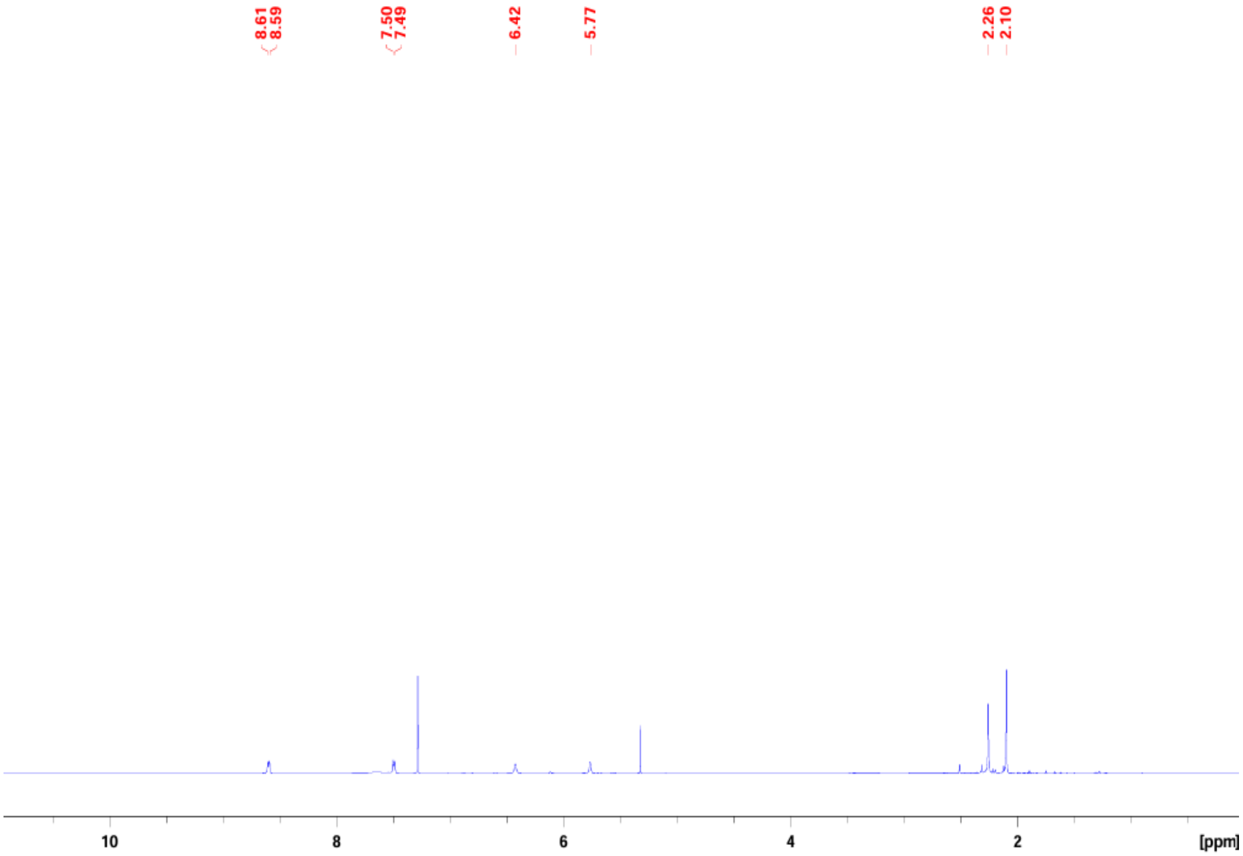

(B)

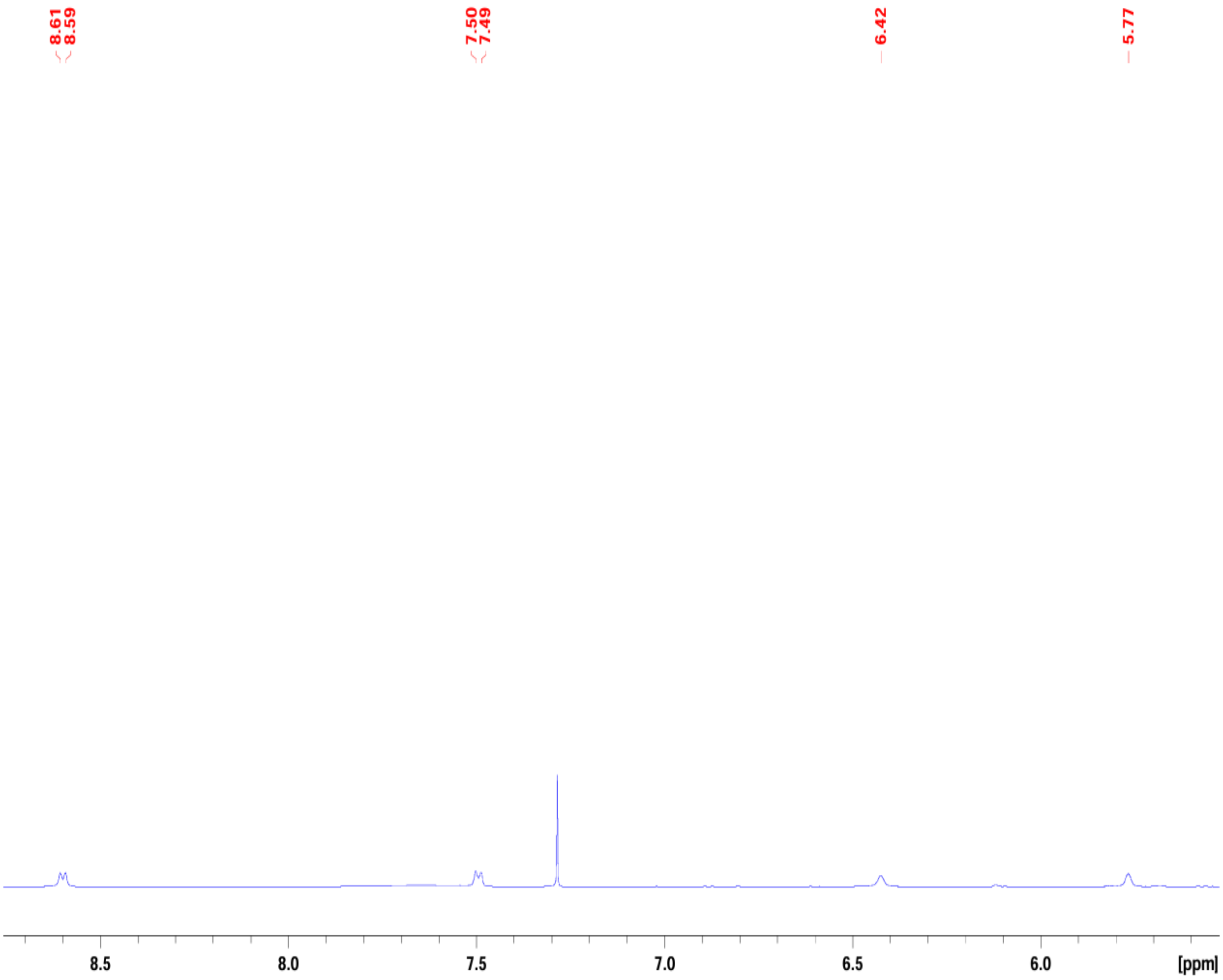

S15: ^{13}C NMR of 4-(2,4-dimethyl-pyrrol-1-yl)pyridine in $CDCl_3$.

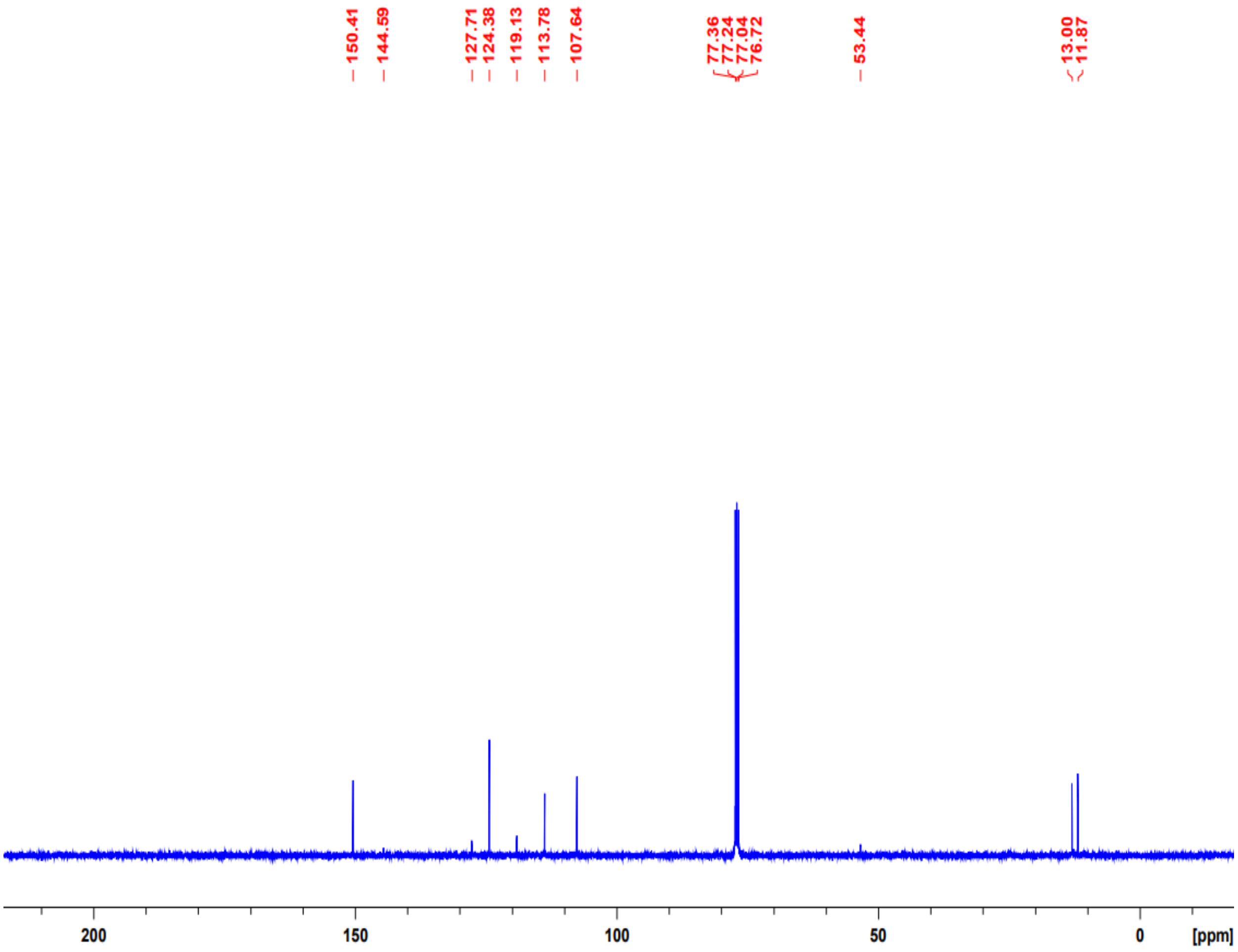

S16: COSY NMR of 4-(2,4-dimethyl-pyrrol-1-yl)pyridine (30 mM) in $CDCl_3$. (A) Full spectrum (B) Aromatic region

(A)

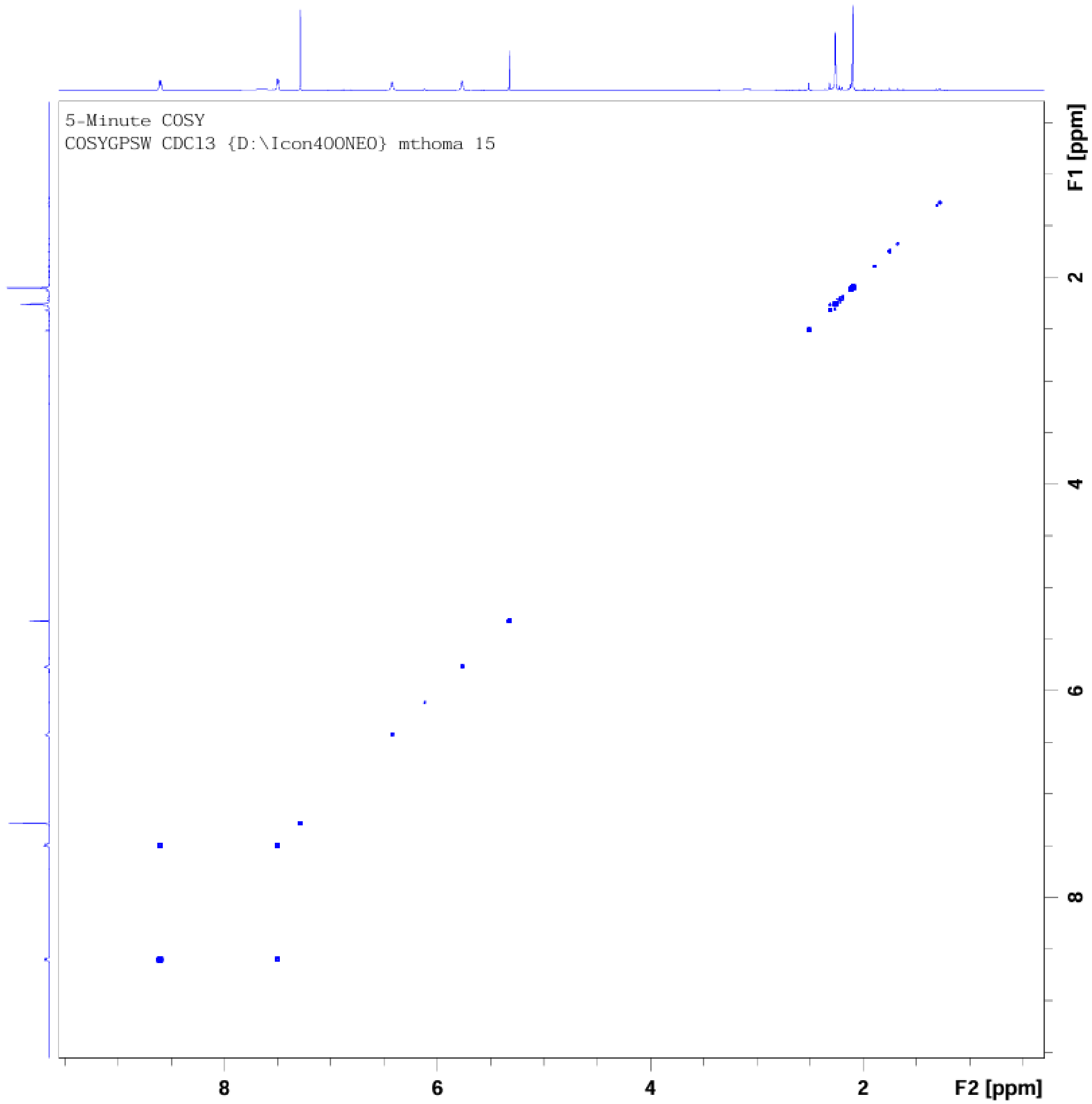

(B)

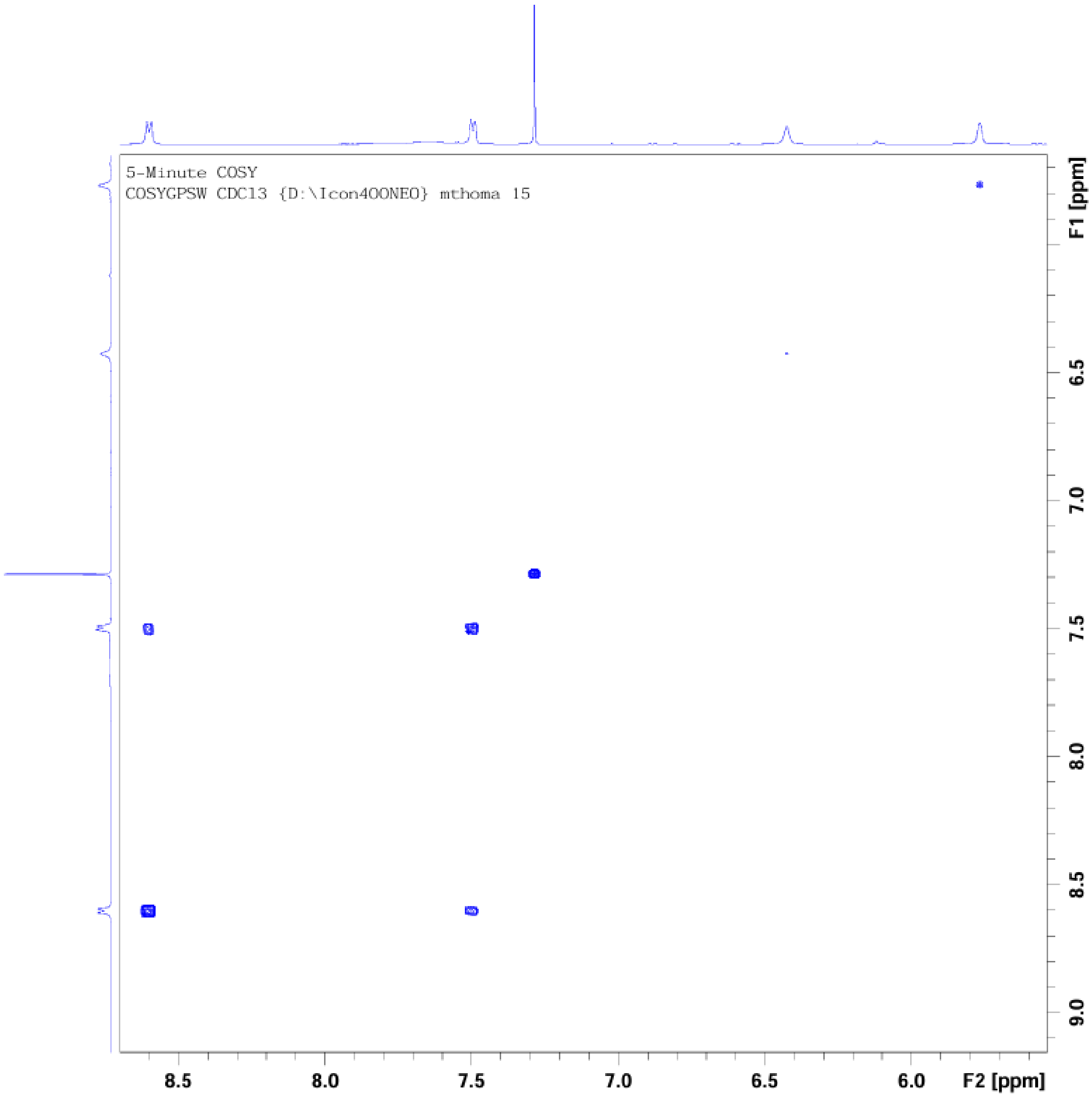
5-Minute COSY
COSYGPSW CDCl3 {D:\Icon400NEO} mthoma 15
F1 [ppm]
6.5
7.0
7.5
8.0
8.5
9.0
8.5
8.0
7.5
7.0
6.5
6.0
F2 [ppm]

S17: HSQC NMR of 4-(2,4-dimethyl-pyrrol-1-yl)pyridine (30 mM) in $CDCl_3$.

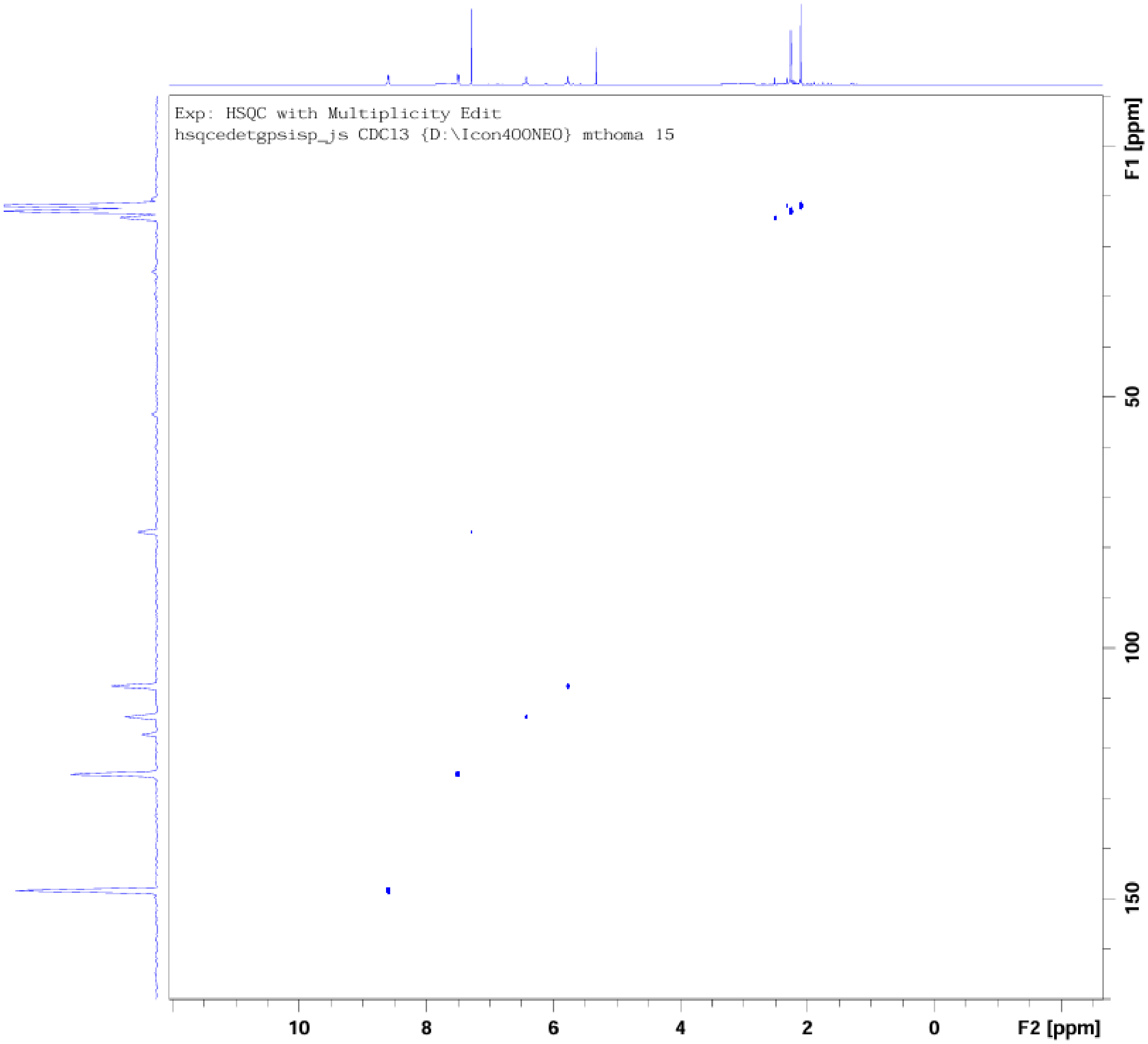

S18: ^{1}H NMR of 4-(2,4-dimethyl-pyrrol-1-yl)pyridine (90 mM) in $CDCl_3$. (A) Full spectrum (B) Aromatic region

(A)

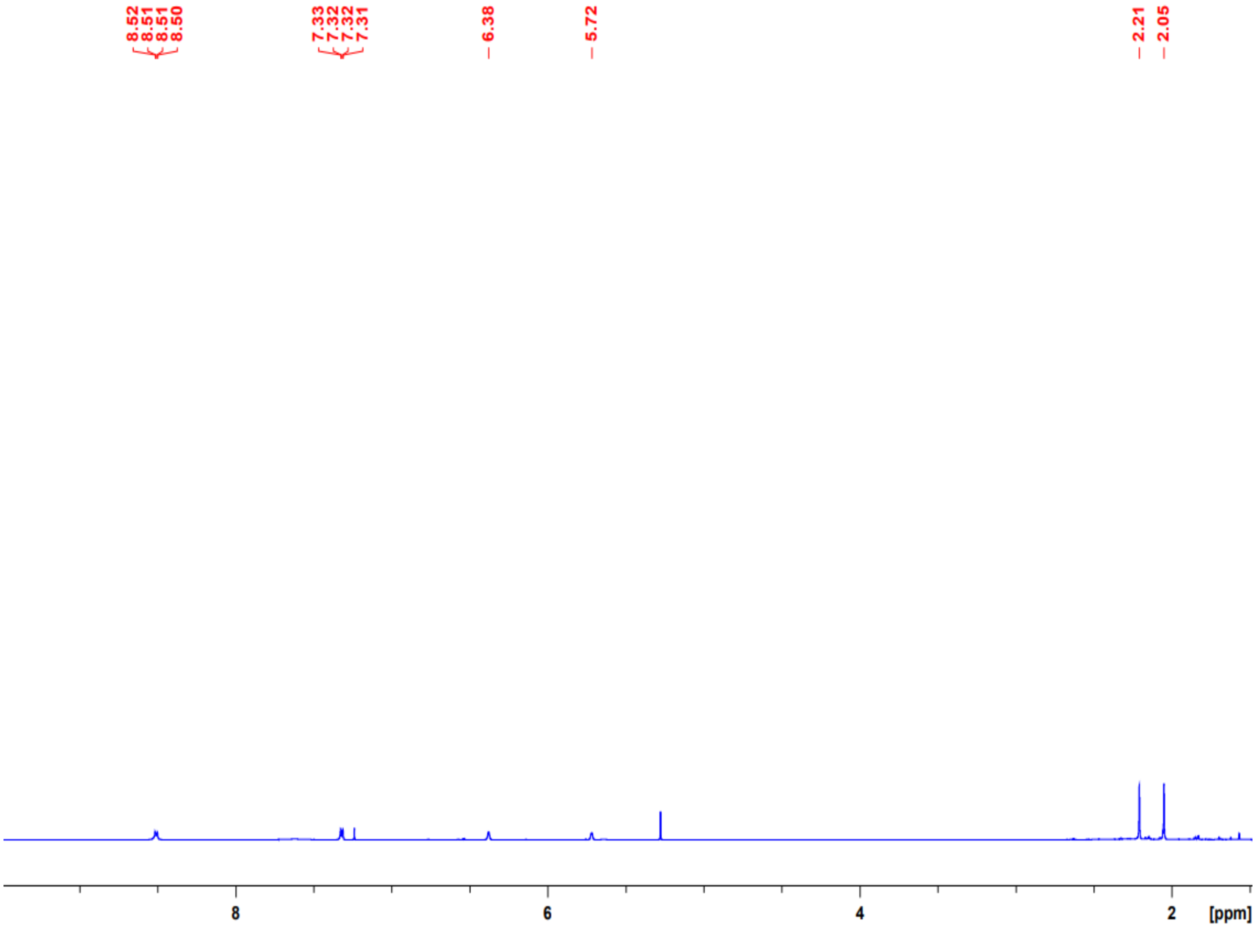

(B)

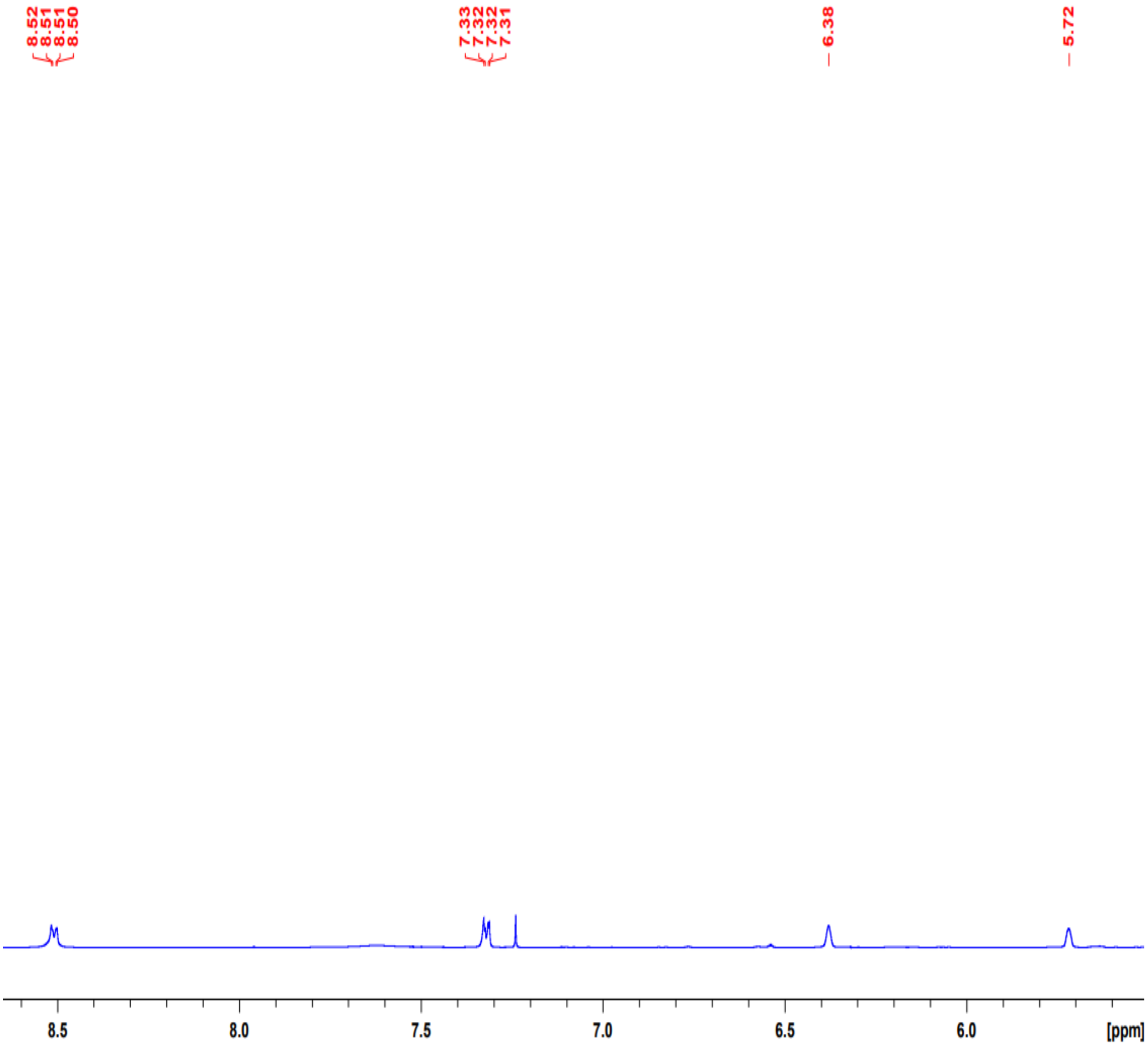

S19: ^{1}H NMR of 4-(2,4-dimethyl-pyrrol-1-yl)pyridine (120 mM) in $CDCl_3$. (A) Full spectrum (B) Aromatic region

(A)

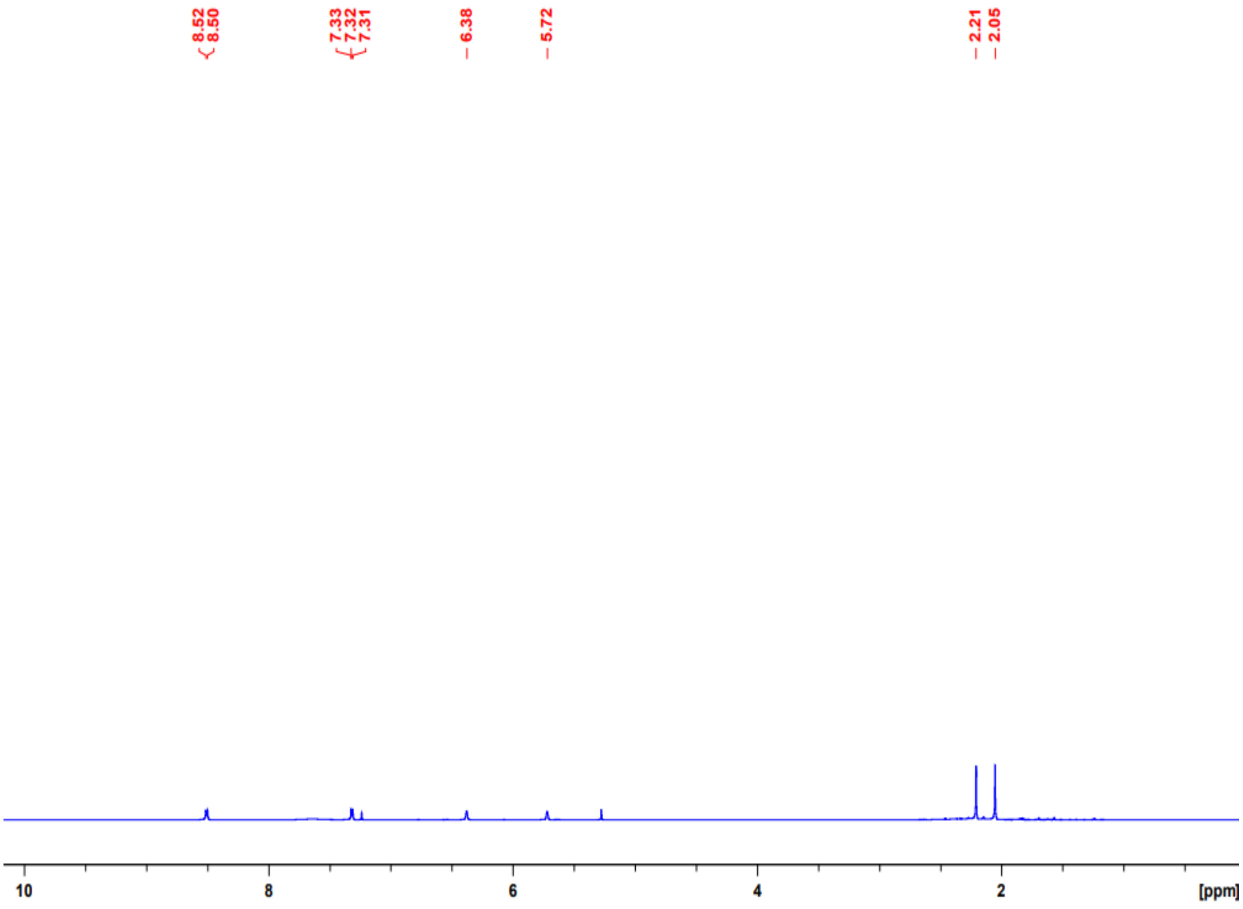

(B)

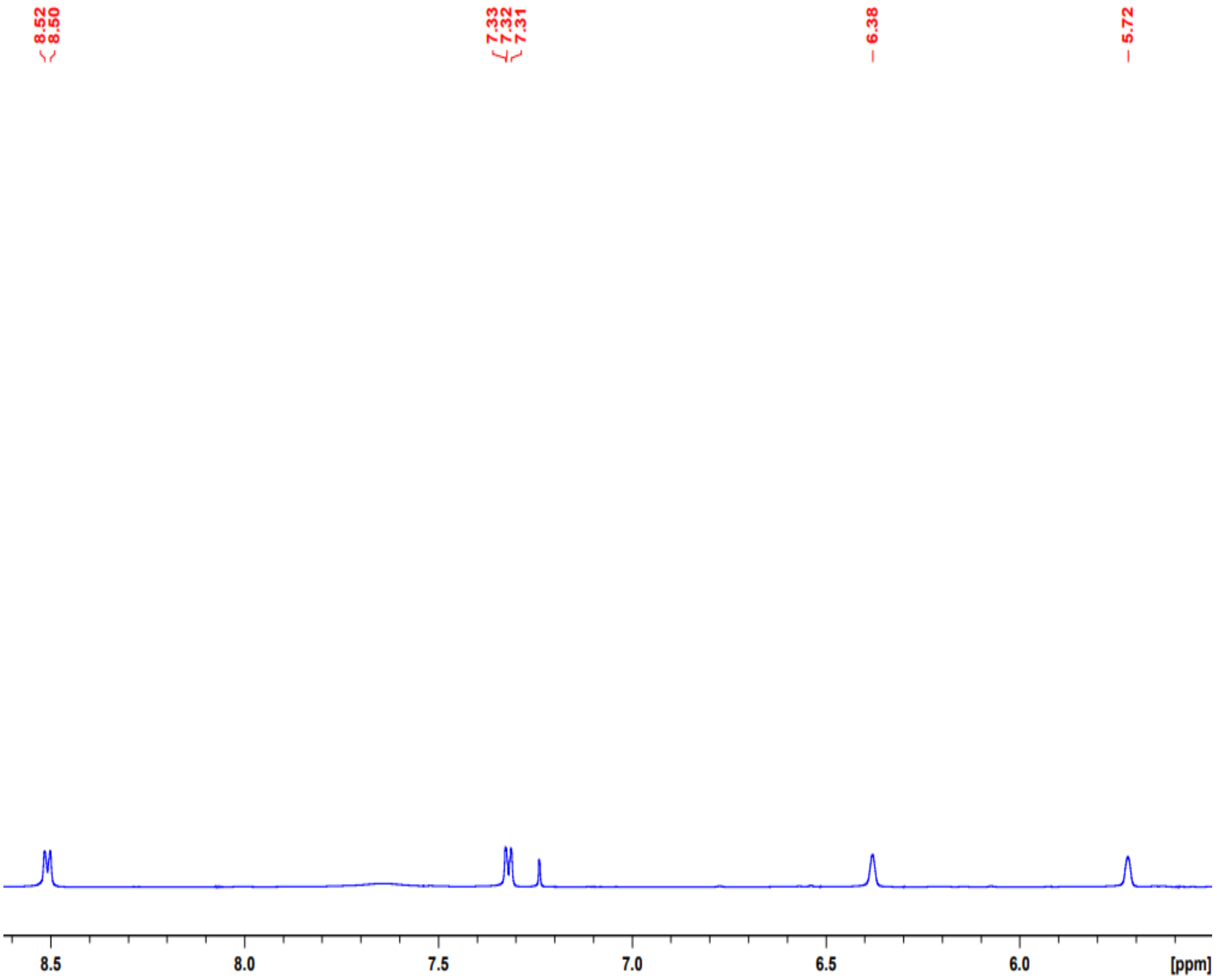

S20: Comparison of 30 mM (blue), 90 mM (red), and 120 mM (green) ^{1}H NMR spectra of 4-(2,4-dimethyl-pyrrol-1-yl)pyridine in $CDCl_3$.

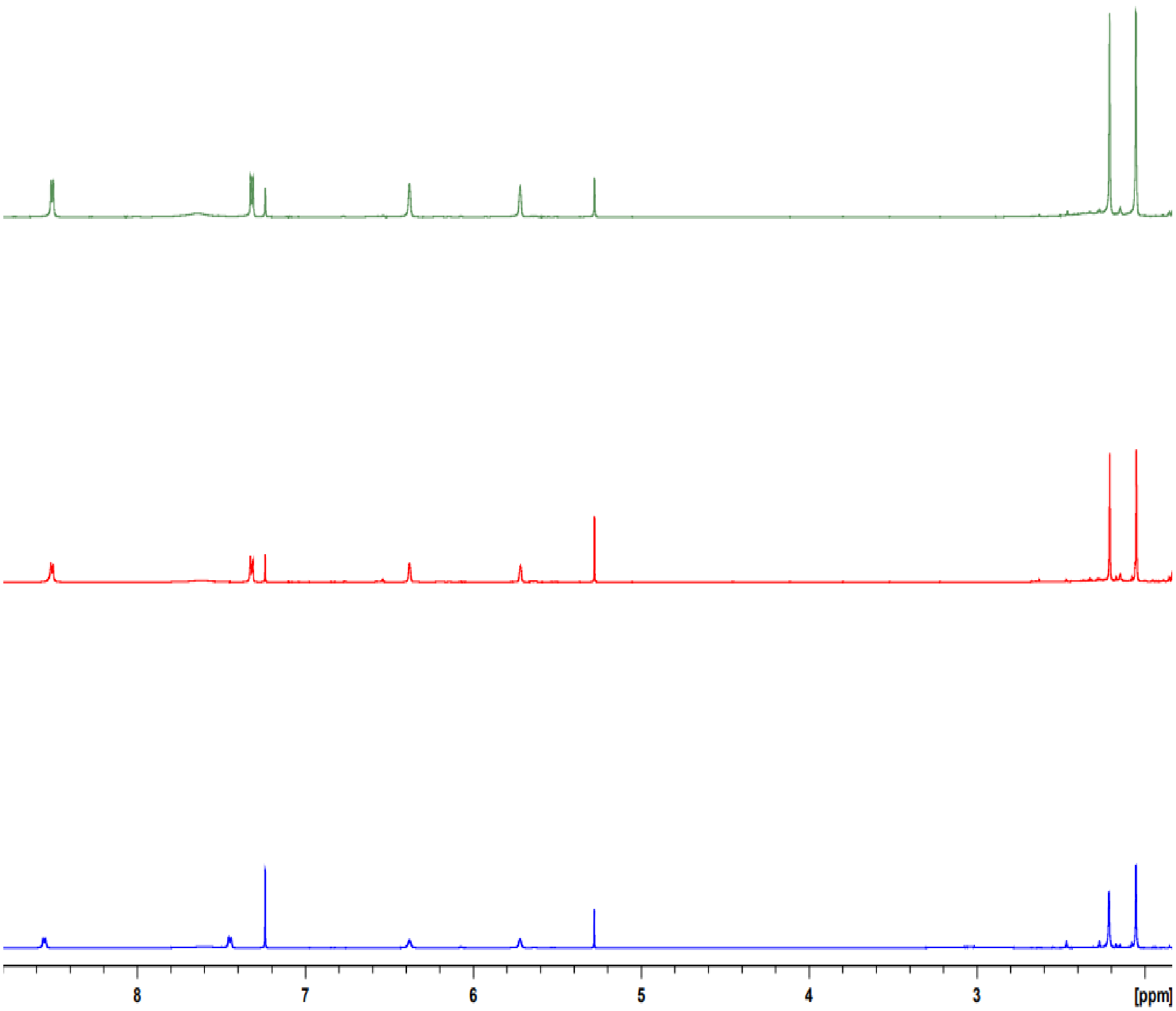

S21: ^{1}H NMR of 4-(2,4-dimethyl-pyrrol-1-yl)pyridine (30 mM) in d-DMSO. The peak at 5.75 ppm is indicative of leftover dichloromethane solvent, and 3.32 ppm is indicative of water. Both trace molecules do not affect the spectrum of the molecule (A) Full spectrum (B) Aromatic region

(A)

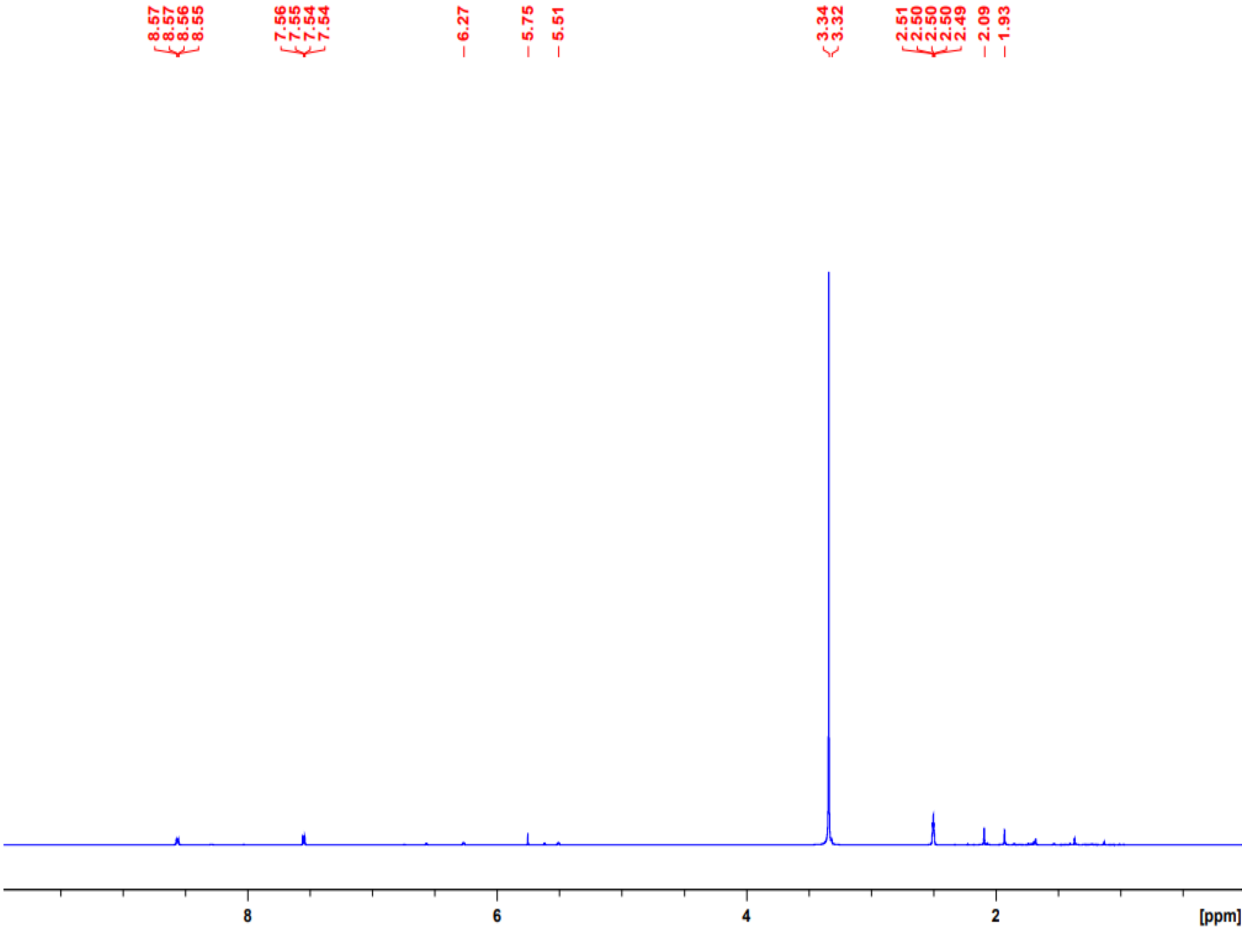

(B)

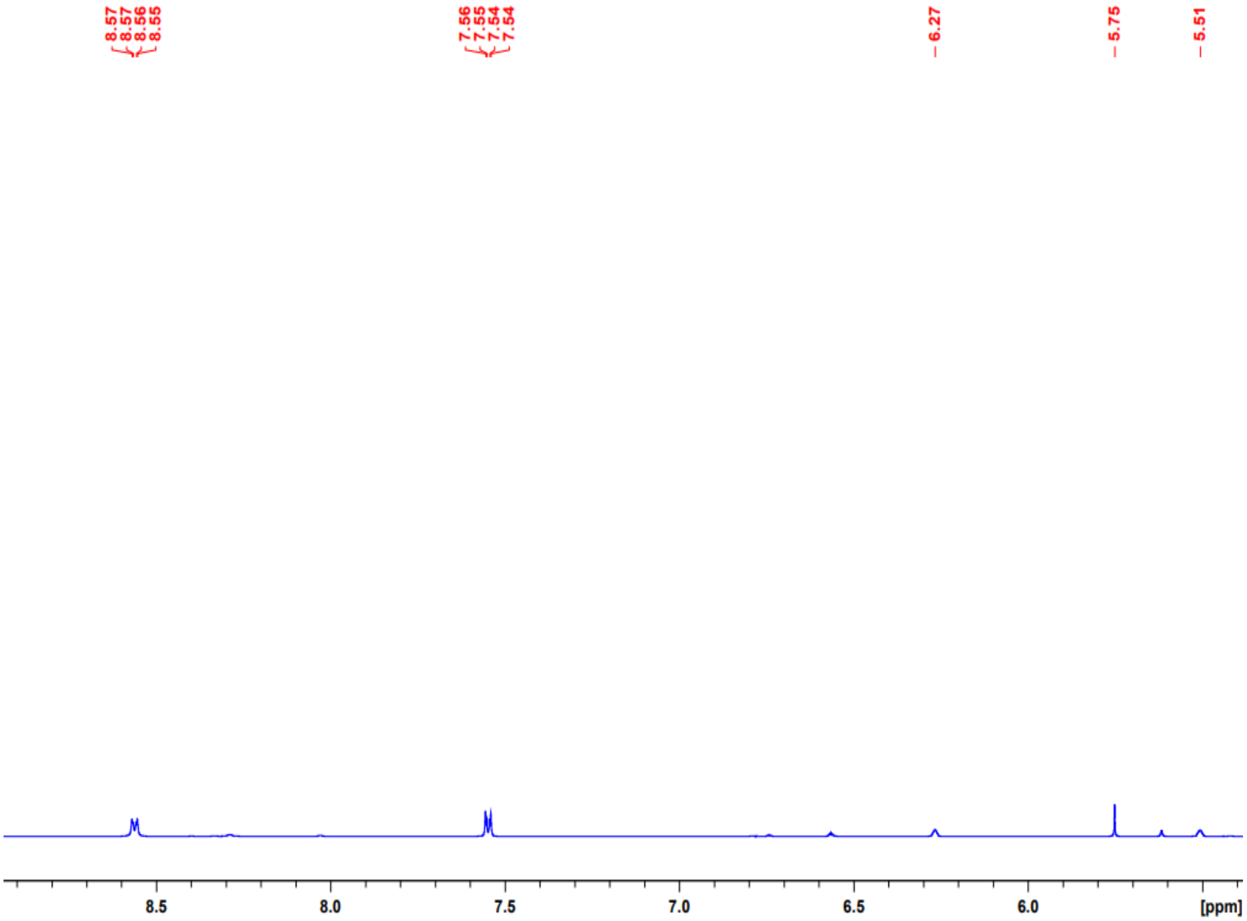

S22: ^{1}H NMR of 4-(2,4-dimethyl-pyrrol-1-yl)pyridine (90 mM) in d-DMSO. The peak at 5.75 ppm is indicative of leftover dichloromethane solvent and 3.32 ppm is indicative of water. Both trace molecules do not affect the spectrum of the molecule. (A) Full spectrum (B) Aromatic region

(A)

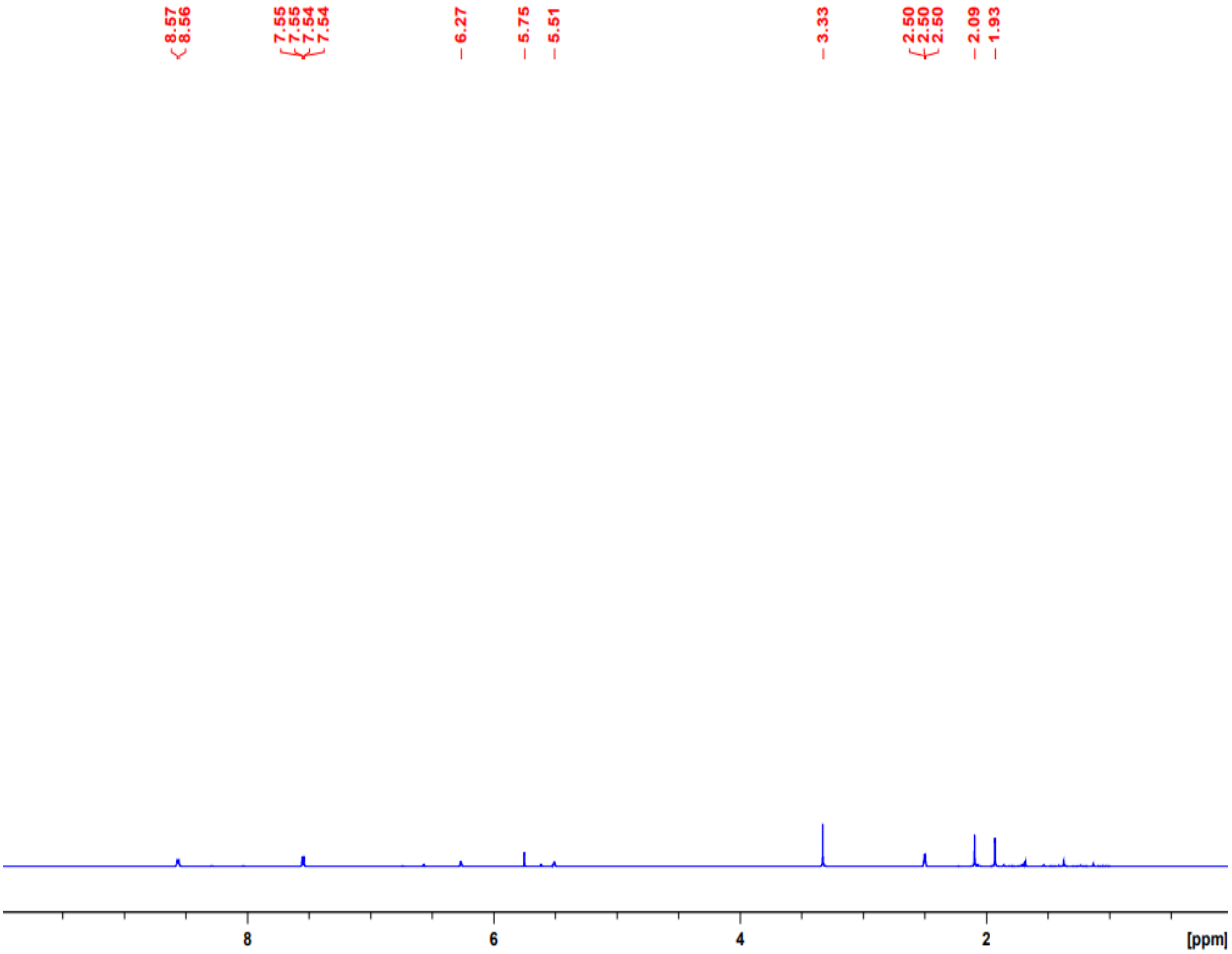

(B)

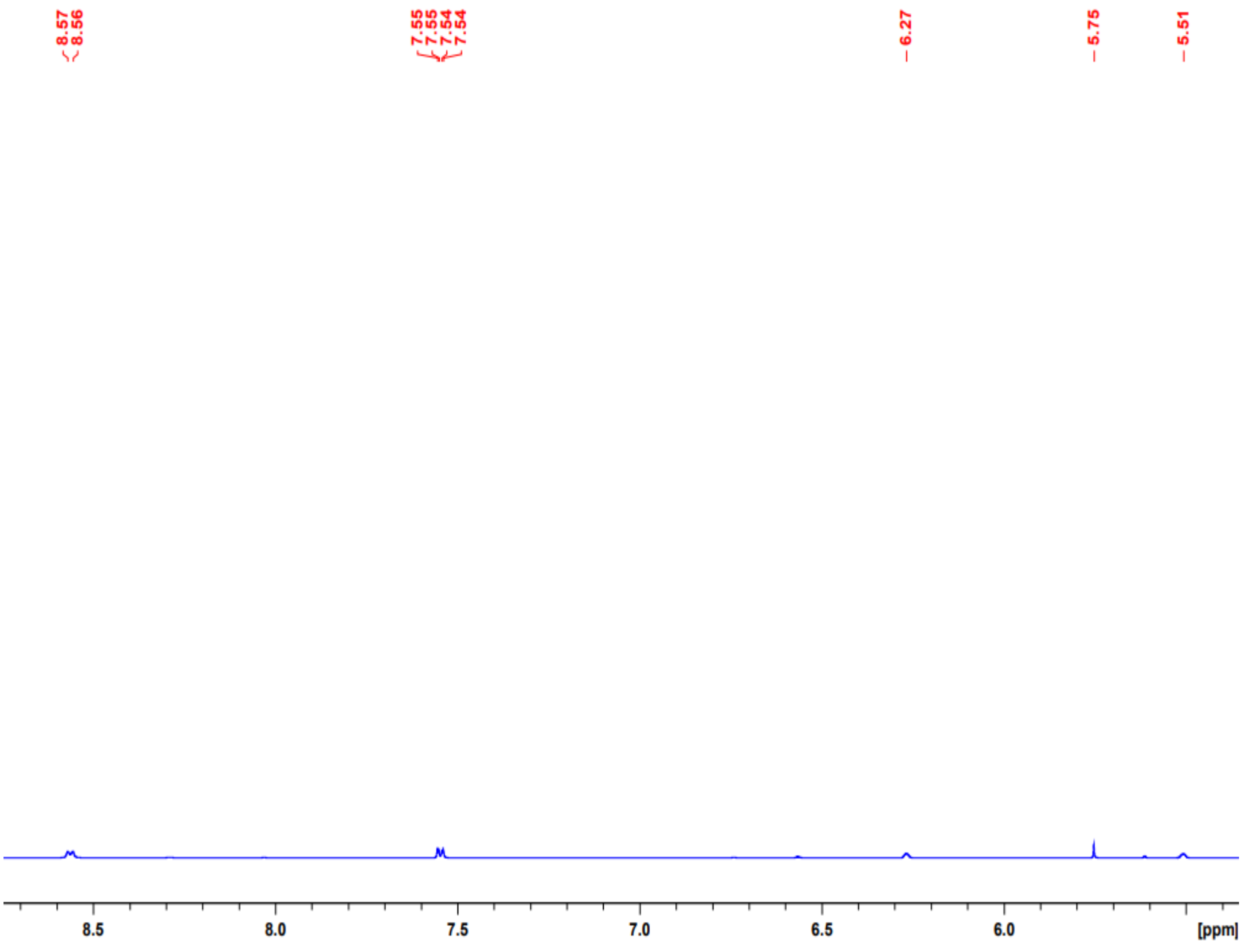

S23: ^{1}H NMR of 4-(2,4-dimethyl-pyrrol-1-yl)pyridine (120 mM) in d-DMSO. The peak at 5.75 ppm is indicative of leftover dichloromethane solvent and 3.32 ppm is indicative of water. Both trace molecules do not affect the spectrum of the molecule . (A) Full spectrum (B) Aromatic region

(A)

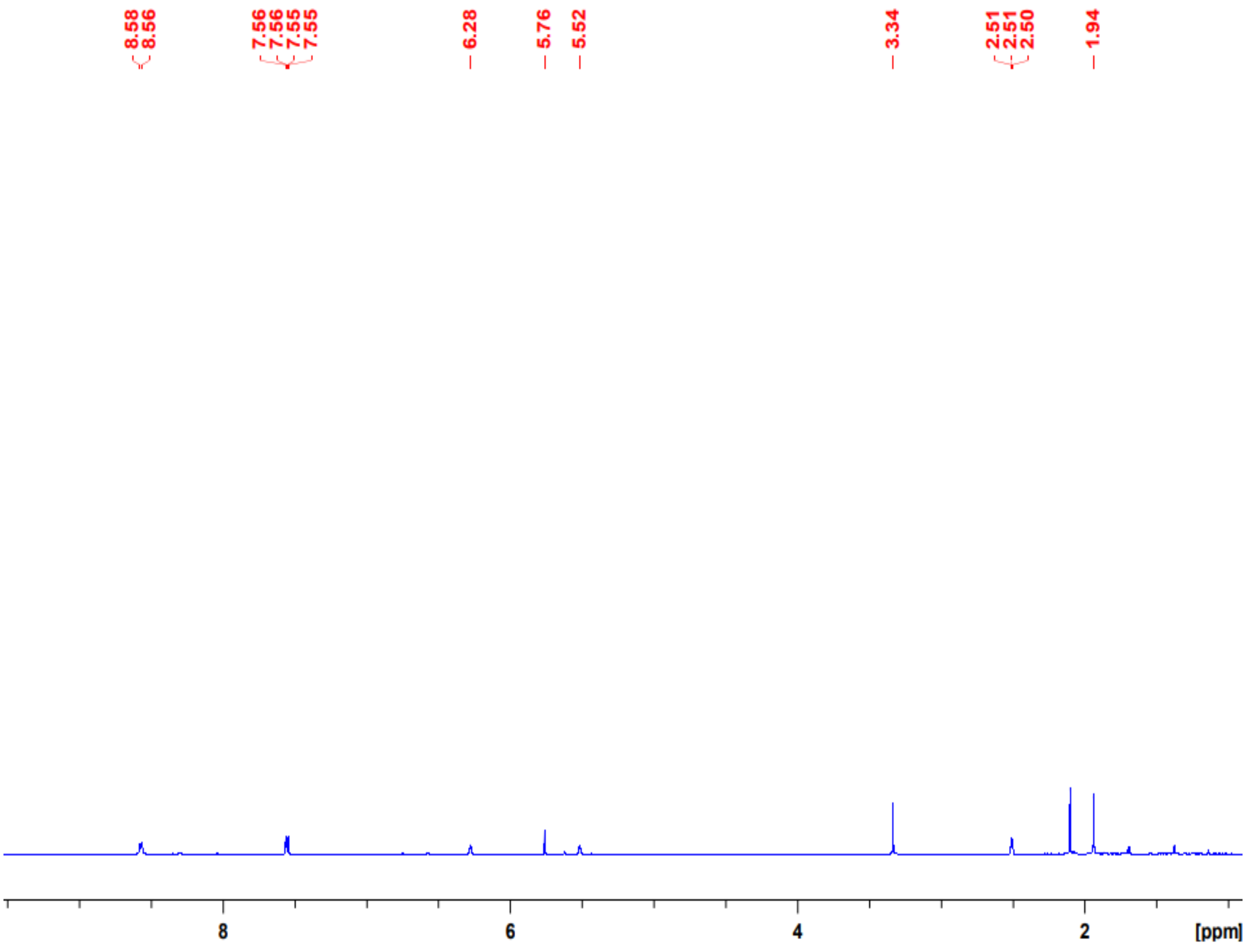

(B)

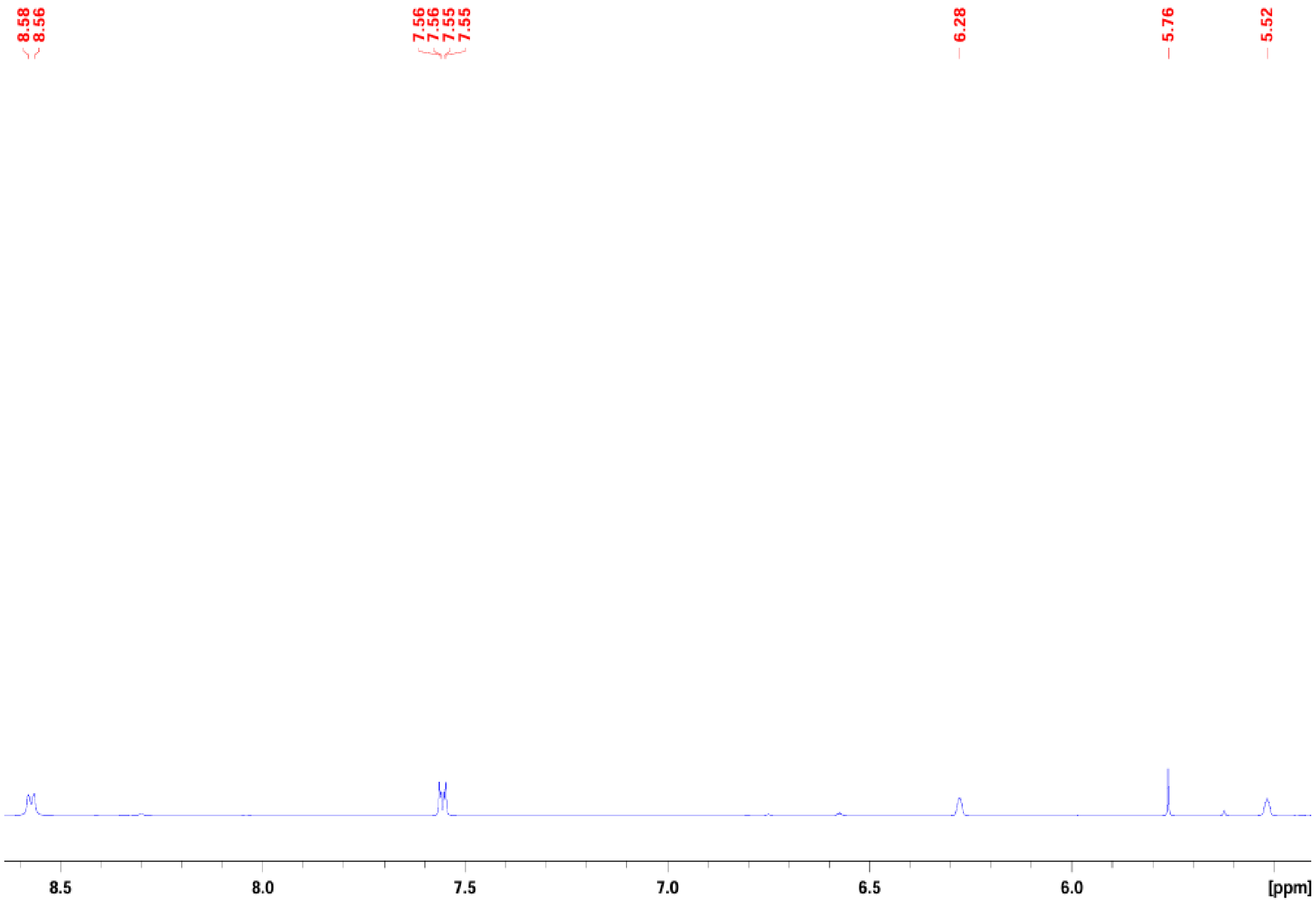

S24: Comparison of 30 mM 4-(2,4-dimethyl-pyrrol-1-yl)pyridine in $CDCl_3$ (blue) and 120 mM 4-(2,4-dimethyl-pyrrol-1-yl)pyridine (red) in d-DMSO in the aromatic region.

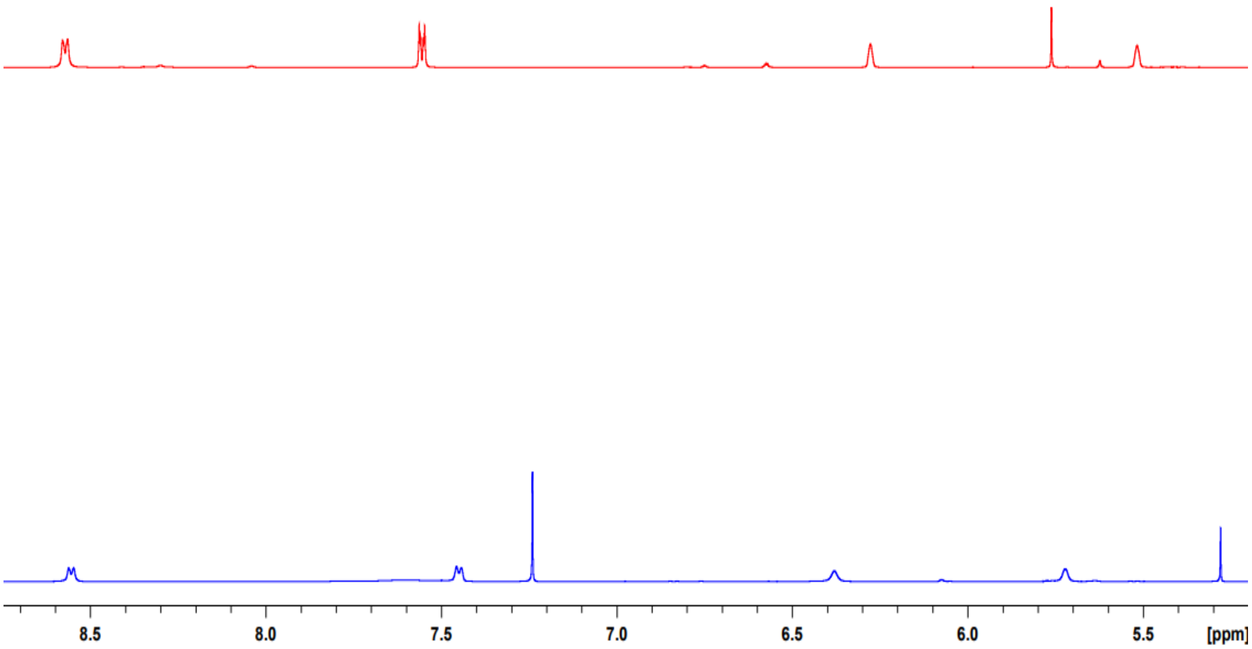

S25: ^{1}H NMR of 4-(2,4-dimethyl-pyrrol-1-yl)pyridine with sodium nitrite in d-DMSO. Peaks at 5.71 ppm and 3.32 ppm are indicative of dichloromethane and water respectively. Both peaks do not affect the spectrum. (A) Full spectrum (B) Aromatic region.

(A)

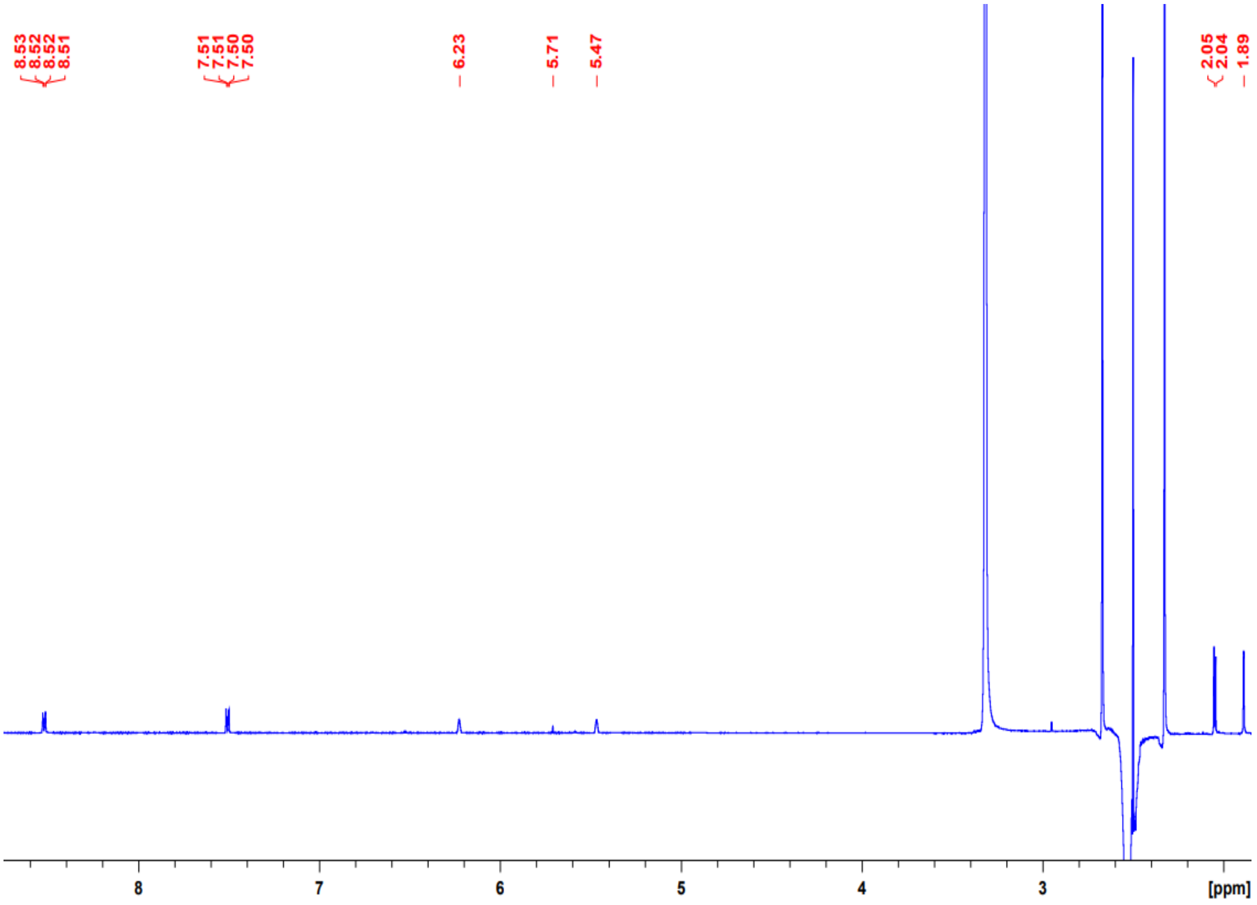

(B)

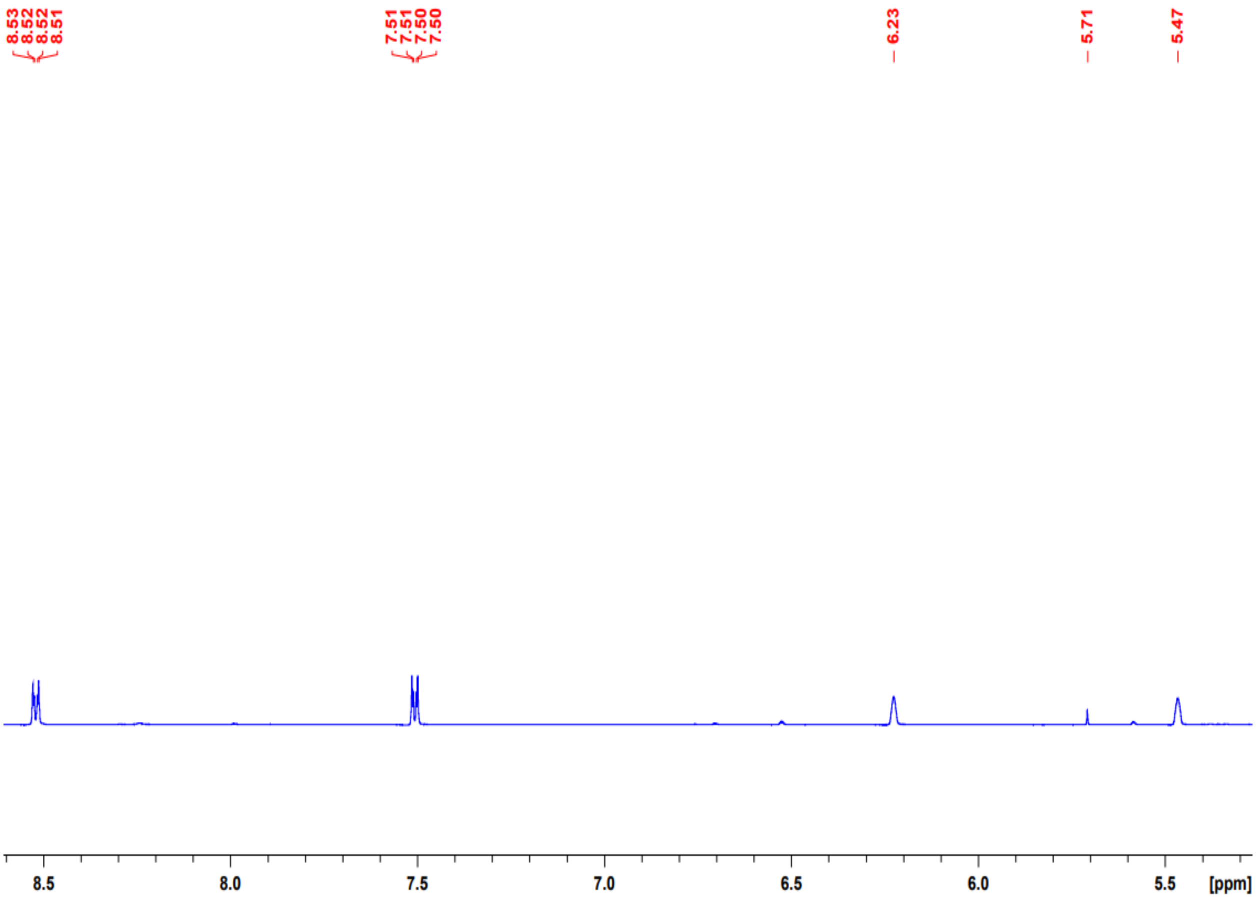
8.53
8.52
8.52
8.51
7.51
7.51
7.50
7.50
6.23
5.71
5.47
8.5
8.0
7.5
7.0
6.5
6.0
5.5
[ppm]

S26: COSY NMR of 4-(2,4-dimethyl-pyrrol-1-yl)pyridine with sodium nitrite in d-DMSO.

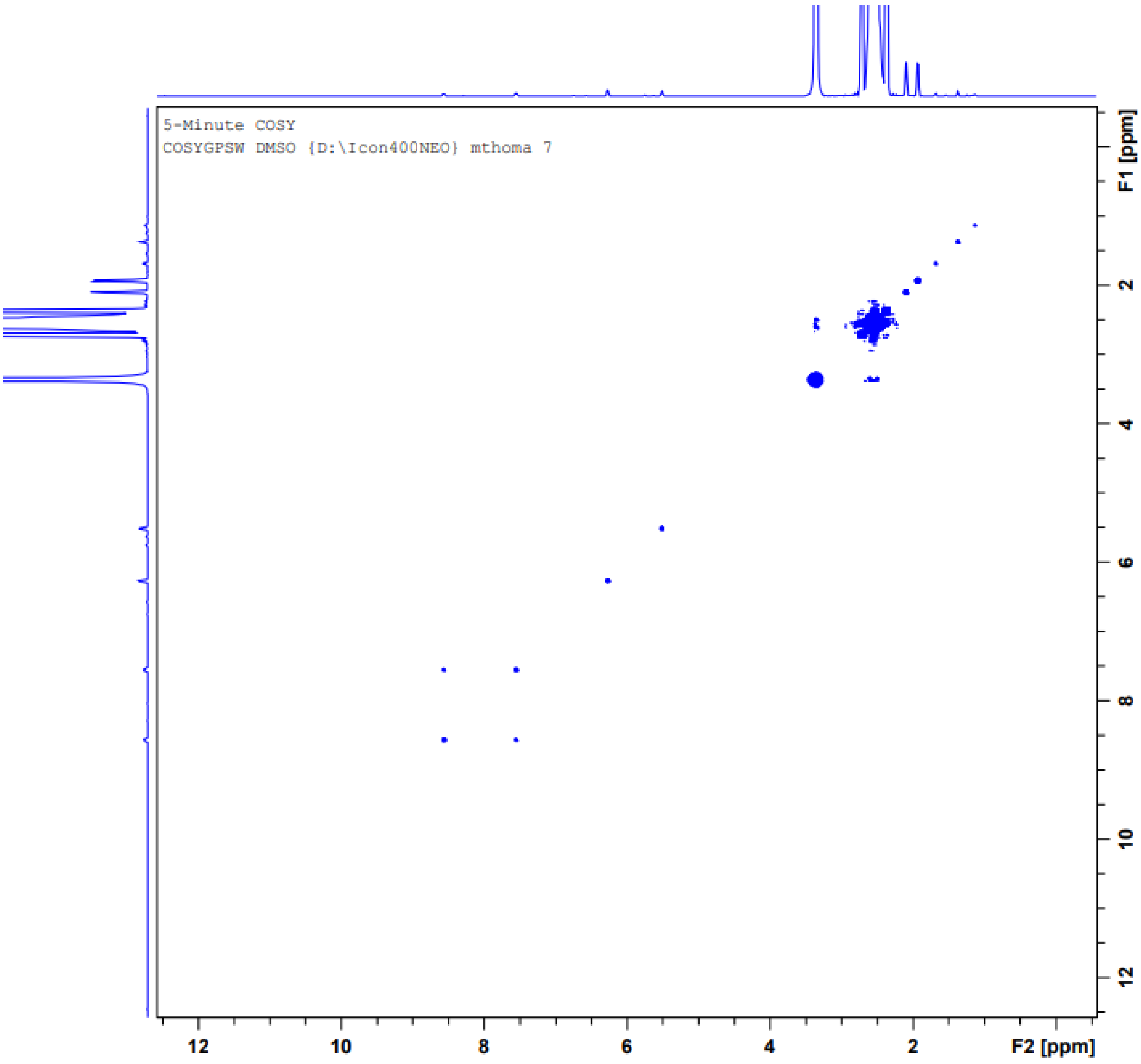

Supplement: Supplementary file 1 [file molecules-29-05692-s001.zip › molecules-3294024-supplementary.pdf]
